# Identification of modifiable factors associated with owner-reported equine laminitis in Britain using a web-based cohort study approach

**DOI:** 10.1186/s12917-019-1798-8

**Published:** 2019-02-12

**Authors:** D. Pollard, C. E. Wylie, K. L. P. Verheyen, J. R. Newton

**Affiliations:** 1 Epidemiology Department, Centre for Preventive Medicine, Animal Health Trust, Newmarket, Suffolk, UK; 2 Rossdales Equine Hospital, Exning, Newmarket, UK; 3 University of Sydney, School of Medical Sciences, Camperdown, Sydney, Australia; 4 Veterinary Epidemiology, Economics and Public Health Group, Pathobiology and Population Sciences, Royal Veterinary College, North Mymms, Hertfordshire, UK

**Keywords:** Horse, Pony, Incidence rate, Risk factor, Cox regression, Hazard rate

## Abstract

**Background:**

Equine laminitis is a complex disease that manifests as pain and lameness in the feet, often with debilitating consequences. There is a paucity of data that accounts for the multifactorial nature of laminitis and considers time-varying covariates that may be associated with disease development; particularly those that are modifiable and present potential interventions. A previous case-control study identified a number of novel, modifiable factors associated with laminitis which warranted further investigation and corroboration. The aim of this study was to identify factors associated with equine laminitis in horses/ponies in Great Britain (GB) using a prospective, web-based cohort study design, with particular interest in evaluating modifiable factors previously identified in the case-control study.

**Results:**

Self-selected horse/pony owners in GB submitted initial baseline and follow-up health and management questionnaires for 1070 horses/ponies between August 2014 and December 2016. The enrolled horses/ponies contributed 1068 horse-years at risk with a median of 38 days between questionnaire submissions. Owners reported 123 owner-recognised and/or veterinary-diagnosed episodes of active laminitis using a previously-validated laminitis reporting form. Multivariable Cox regression modelling identified 16 risk/protective factors associated with laminitis development. In keeping with the previous case-control study, a prior history of laminitis (particularly non-veterinary-diagnosed episodes), soreness after shoeing/trimming and weight gain were associated with higher rates of laminitis. There is now strong evidence that these risk factors should be used to guide future recommendations in disease prevention. Factors with some prior evidence of association included breed, steroidal anti-inflammatory administration, transport and worming. The modifiable factors amongst these should be the focus of future laminitis studies. The remainder of the identified factors relating to health, turnout and grazing management and feeding are novel, and require further investigation to explore their relationship with laminitis and their applicability as potential interventions.

**Conclusions:**

This study has demonstrated a temporal relationship between a number of horse- and management-level factors and laminitis, identifying potential interventions and important risk groups for which these interventions would be of particular importance. These results serve as a sound evidence-base towards the development of strategic recommendations for the horse/pony-owning population to reduce the rate of laminitis in GB.

**Electronic supplementary material:**

The online version of this article (10.1186/s12917-019-1798-8) contains supplementary material, which is available to authorized users.

## Background

Equine laminitis is a complex, multifactorial disease that manifests itself in the foot when a cascade of inflammatory, endocrine and/or vascular reactions cause disruption to the structure and function of the suspensory apparatus of the distal phalanx (SADP) [1]. Pain-inducing pathological changes within the foot are observed as changes in stance and gait and, if the damage is extensive enough, permanent changes to future hoof horn growth can occur with consequent alterations in foot anatomy [2, 3]. The clinical signs herald potentially irreversible damage within the lamellar region of the foot, often with debilitating consequences, making identification of high risk animals and prevention of incident episodes paramount in reducing the frequency of equine laminitis [2–5].

Few robust epidemiological studies have been conducted to ascertain the animal- and management-level factors that contribute to laminitis development [6]. A 2012 systematic review and quality appraisal of peer-reviewed evidence for laminitis risk factors concluded that there was weak and/or inconsistent evidence for associations between factors such as sex, breed, body weight, seasonality and laminitis [6]. Subsequent to the systematic review, a further two cohort studies in Great Britain (GB) [7, 8] and three case-control studies in GB, Denmark and the United States and Canada [9–11] have contributed to laminitis epidemiology. These studies have provided further evidence of an association between laminitis and metabolic factors [7], feeding [9, 11], grazing [9, 10], signalment [8–10], glucocorticoid administration [8, 11], co-morbidity [8, 9, 11] and obesity [9, 11]. Within GB a number of novel, modifiable management factors that could aid in laminitis prevention were identified. These included owner-perceived weight gain, new access to grass, box rest, lameness following shoeing/trimming, increasing time since last worming as risk factors, and transport and feeding of supplements as protective factors [9]. However, there remains a paucity of data that accounts for the multifactorial nature of laminitis and considers time-varying covariates that may be associated with disease development. A large-scale cohort study would establish the temporal relationship between factors under investigation and laminitis occurrence. Previous epidemiological studies of laminitis have largely relied on veterinary-diagnosed episodes, but with up to 50% of episodes reported not to be veterinary-diagnosed [12, 13], important associations may not yet have been identified.

The aim of the current study was to identify factors associated with owner-reported equine laminitis (i.e. including, but not exclusively, cases diagnosed by a veterinary surgeon) in horses/ponies in GB using a prospective cohort study design, with particular interest in evaluating modifiable factors previously identified in a case-control design [9].

## Methods

### Study design, study period and sample size estimation

A prospective, dynamic web-based cohort study was conducted in GB between August 2014 and December 2016 (29 months). Recruitment of participants was based on self-selection of individuals who owned or cared full-time for a horse/pony that resided in England, Scotland or Wales. Prior to, and for the duration of, the recruitment period (August 2014 to July 2016), the study was advertised nationally across a variety of equestrian and veterinary media platforms and/or organisations. Owners enrolling only some of their animals were instructed to enrol those whose names appeared first in the alphabet, to avoid bias. A dedicated website hosted the data collection tools and assisted with participant recruitment and retention.

Sample size estimates (OpenEpi v3.03)^1^ suggested a minimum of 1000 horse-years at risk (HYAR) were required based on: (i) owner-reported laminitis prevalence estimates of between 3 and 7%, derived from an anticipated higher prevalence compared to using solely veterinary-diagnosed cases [14], (ii) an exposure prevalence range between 5 and 30%, (iii) minimum detection of a 2-fold increase in risk across exposures with 95% confidence and 80% power and (iv) 20% losses-to-follow-up.

### Data collection

Participants were required to submit a baseline management and health questionnaire (hereafter referred to as ‘the questionnaire’), supplemented with regular consecutive follow-ups and the reporting of incident active equine laminitis episodes. Where internet access was limited, participation was facilitated by posted questionnaires and structured telephone/in-person follow-ups. Retention of participants was aided by automated monthly reminder e-mails and manual personalised e-mails in instances where no follow-ups had been obtained for more than 3 months. Study updates were e-mailed to all participants approximately every 2 months and regular incentives, mainly in the form of prize draws and competitions, were used to attract new participants and encourage follow-up submissions.

#### Questionnaire

The questionnaire (Additional file 1) consisted of eight sections regarding the animal’s (i) signalment, (ii) turnout and management of grazing, (iii) stabling and indoor environment, (iv) feeding, (v) exercise, (vi) transport, (vii) hoof care, (viii) health management and recent and current health, and was based on a previous owner questionnaire that collected data on risk factors for veterinary-diagnosed laminitis in GB [9]. An online weight tracker was developed to monitor temporal fluctuations in estimated body weight (BW) using anatomical measurements [15], explained in an accompanying instruction booklet (Additional file 2). Unless otherwise stated, participants were instructed to provide information as on the day of questionnaire completion. Follow-ups were invited every 1 to 3 months, and were provided by editing the saved baseline/previous entry. Submission of a baseline questionnaire signalled the entry of a horse/pony into the study and exit from the study was the day after their last follow-up submission.

#### Owner laminitis reporting form (LRF)

Participants were asked to complete a LRF for each new, active episode of laminitis their horse/pony developed during the study [13, 14, 16]. The LRF was a checklist of common clinical signs involving lameness, gait, stance and characteristics of the most severely affected foot/feet (Additional file 3). A new laminitis episode was defined as ‘*recognition of active laminitis for the first time or after the horse had returned to its previous level of soundness and activity following a previous episode, without receiving analgesics (such as phenylbutazone) for 14 days or more*’. An active episode was described as ‘*recognition of pain in one or more feet attributed to laminitis, with stance and/or gait abnormalities*’. A default laminitis ‘recovery period’ of 44 days was used for episodes where recovery time was missing, based on the median recovery time estimated from historical episodes in the study population that occurred prior to study enrolment [13].

### Data analyses

Web-based data were stored in a secure structured query language database, exported into Microsoft Excel (v.2010)^2^ for initial processing and imported into Stata (IC v.15.0)^3^ for coding and statistical analyses. A binary outcome variable specified whether laminitis did (1) or did not (0) occur during each submission period. Continuous variables were assessed for normality of distribution and summarised as means and standard deviation or medians and interquartile ranges (IQR) as appropriate. Categorical variables were summarised as proportions with 95% confidence intervals (CI).

Data were declared as survival-time data using the ‘*stset*’ Stata command [17]. This enables analysis of multiple-record-per-subject survival data by specifying the unique animal ID to identify submissions belonging to the same animal, with multiple submissions defining sequential fragments of time over which the animal was observed. ‘Failure’ was defined as presence of laminitis, with the date of failure being the date clinical signs were first recognised. A horse/pony was considered at risk of developing laminitis when they were first enrolled, and remained at risk each day they were enrolled, excluding days when they were recovering from an incident active laminitis episode. Entry into the study for animals which had laminitis at the time of enrolment was delayed until either an episode-specific or default recovery period had passed and their initial LRFs were excluded. Multiple LRFs per animal were allowed, provided the recovery periods for the episodes did not overlap, with temporary exit from the cohort being the date the first clinical signs of laminitis were observed, and re-entry to the cohort following either the reported episode-specific or default recovery period. Where recovery periods overlapped, this was considered as one episode and the start of the recovery period was set as the date of the last recurrence of that episode.

Time to event analysis was conducted using multivariable Cox regression methods [18], including variables associated with laminitis at a level of *P* < 0.25 in univariable analyses and a priori potential confounders [6]. *P*-values were not adjusted for multiple comparisons [19]. Where there was evidence against linearity (likelihood ratio statistic [LRS] *P*-value < 0.05), continuous variables were recoded into biologically plausible or quartile categories. A reference category for each categorical variable was based on the category with the lowest expected risk and sufficient sample size [20]. Missing data remained missing, unless non-time-varying data present at baseline were missing in subsequent follow-ups (e.g. breed, height, coat colour), or if owners indicated no changes since last follow-up, in which case data from previously-available records filled subsequent missing records.

Nine interim multivariable models were created, by grouping related variables together based on questionnaire sections with a separate interim model for variables related to a previous history of laminitis, using manual forward stepwise selection [9]. Where high correlation (Pearson correlation coefficients > 0.80) between variables occurred, those with the most likely biological link to laminitis and/or the most complete data were selected for inclusion [21]. Likelihood ratio tests (LRT) were used to compare nested models with variables retained in the model if LRS *P* < 0.05. Variables retaining significance in the interim models were similarly pooled to develop a final multivariable model using a similar forward stepwise approach. Variables significant in the interim models, but excluded from the final model, were forced back in to ensure that statistically significant or confounding variables were not inadvertently omitted. Potentially relevant, biologically plausible two-way interactions between variables in the final model were assessed using the LRT, retaining those where LRS *P* < 0.05. Clustering at the owner-level was assessed using the *vce (cluster owner_id)* Stata option which allowed for intragroup correlation within animals from the same owner whilst maintaining independency of observations across owners [22]. This clustering option produced robust standard errors by adjusting the variance estimates of each exposure variable for the intragroup correlation [23].

Post-fit diagnostic tests included evaluation of model fit using a cumulative hazard plot against partial Cox-Snell (CS) residuals which, for multiple record data, represent the additive contributions to an individual animal’s overall CS residual [22]. The presence of outliers was assessed using partial deviance residual plots while partial likelihood displacement values were used to identify influential observations [24].

## Results

**Fig. 1:**
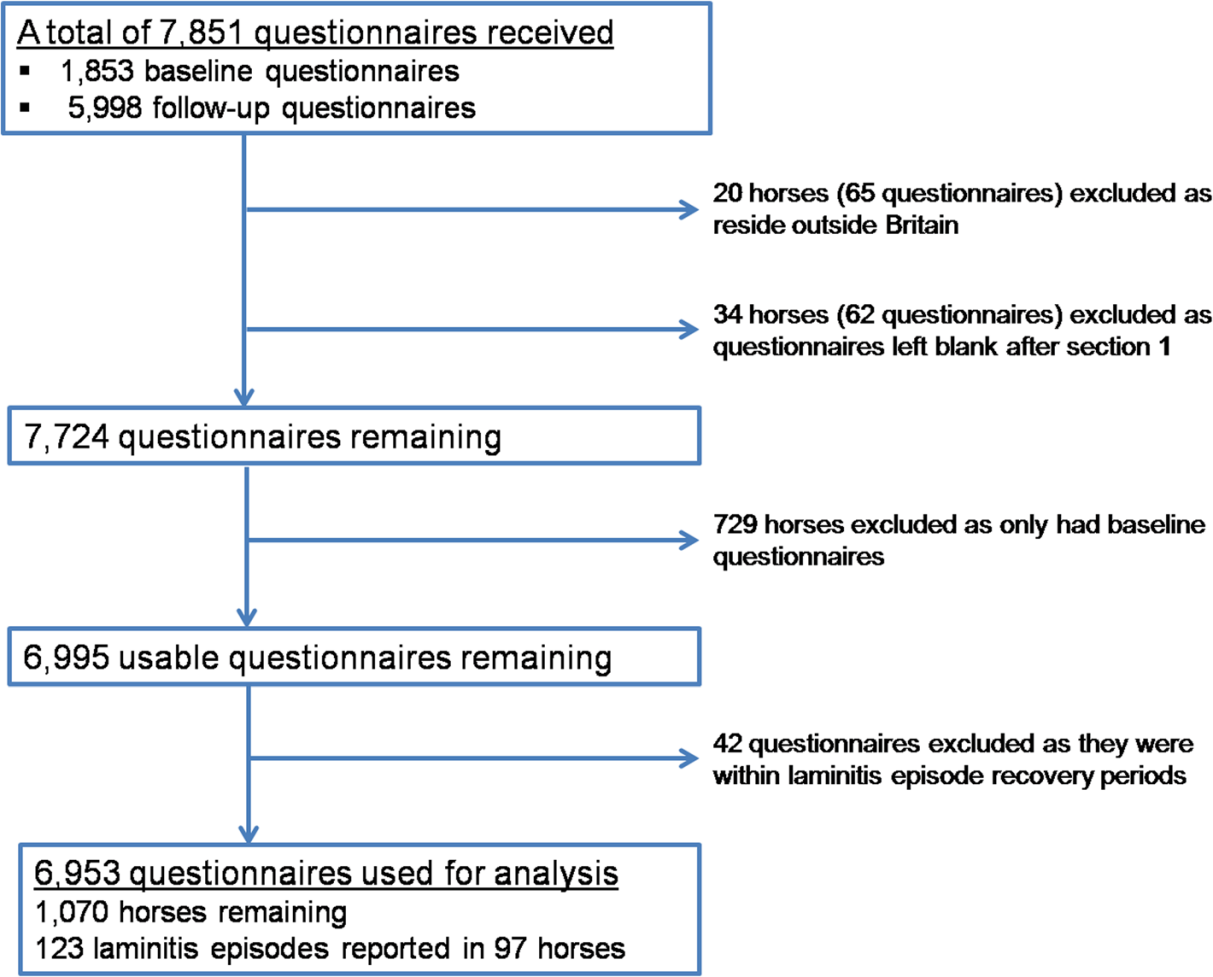
Flow diagram of questionnaire acquisition and exclusion during the laminitis cohort study. Horses represent both horses and ponies

**Fig. 2:**
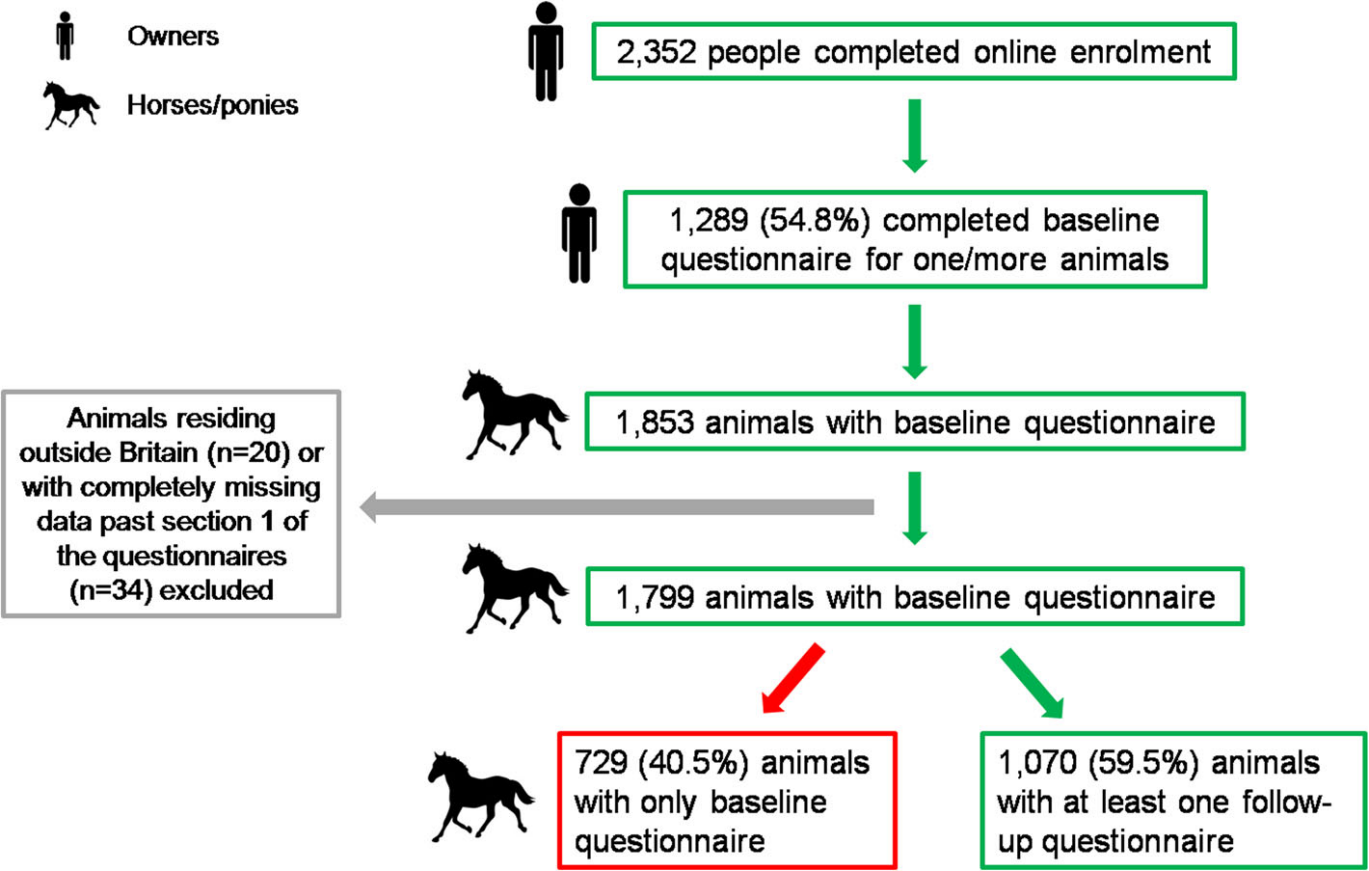
Flow diagram of recruitment and retention of owners and horses/ponies during the laminitis cohort study

A total of 6953 questionnaires, representing 1070 horses/ponies with an accrued time at risk of 1068 HYAR, were available for analysis (Fig. 1). In total, 123 laminitis episodes occurred in 97 horses/ponies, with multiple episodes reported in 19 animals (Fig. 2). The median number of submissions per horse/pony was 4 (IQR 2 to 9 submissions) with median time between submissions 38 days (IQR 30 to 67 days).

The demographics of the baseline study population are provided in Additional file 4. In brief, the median number of horses/ponies enrolled per owner was 2 (IQR 1 to 3 horses/ponies). The population consisted of mainly geldings (57.5%) and native ponies (40.7%) made up the largest breed category. Mean owner-reported age was 14.7 years (±6.9 years; range 1 to 38 years) and median height was 147.3 cm (IQR 135.9 to 157.5 cm). Most of the horses/ponies (59.0%) were reported to be in ideal body condition (3/5 body condition score [BCS]), while 33.6% were overweight (BCS > 3) [25]. Approximately half of the horses/ponies (49.5%) were reported to have an even distribution of fat along the crest, scoring 2/5 on the cresty neck score (CNS) scale [26], while 27.2% had enlarged crests with various degrees of thickening in the middle (CNS > 2). The median duration of ownership/care was 6.7 years (IQR 2.5 to 11.9 years) and owners reported that 35.9% (CI 33.0, 38.7%) of horses/ponies had laminitis previously whilst in their care. Of the 97 horses/ponies that developed laminitis during the study period, 75.3% (CI 66.7, 83.8%) had previous episodes of the disease.

All variables considered for inclusion in the final multivariable Cox regression model are presented in Additional file 5, with interim models presented in Additional file 6. No significant two-way interactions were identified between variables in the final model. Exclusion of highly influential observations had no considerable effect on model parameter estimates, thus all observations were retained in the final model. All variables retained significance following adjustment of standard errors, therefore there was no adjustment for clustering at the owner-level.

The final multivariable model contained 16 variables associated with laminitis development (Table 1). In brief, variables associated with higher rates of laminitis included **animal-level factors:** (i) weight gained during the study vs. lost or maintained weight, (ii) native pony breed vs. all other breeds combined; **laminitis history:** (iii) a previous history of laminitis, particularly when previous episodes were not veterinary-diagnosed, vs. none, (iv) longer recovery periods following the most recent laminitis episode vs. shortest; **health management and recent health history:** (v and vi) recent use of steroidal or non-steroidal anti-inflammatory drugs (NSAIDs) vs. none, (vii) lameness due to soft tissue injury vs. none, (viii) benzimidazoles used for the most recent worming vs. products containing macrocyclic lactones; **hoof care:** (ix) lameness or foot soreness following the most recent trimming or shoeing vs. none, (x) trimming or shoeing frequency greater than 8 weeks vs. more regular trimming; **turnout and grazing management:** (xi) short-term access to grass in the mornings only vs. accessing grass at other times of the day and/or night, (xii) use of grazing muzzles only part of the time vs. not used at all or used the whole time during grazing, (xiii) spending 12 h or less stabled vs. more than 12 h, (xiv) being transported in the previous year mainly due to moving yards vs. being transported for other reasons or not at all, and **supplementary feeding:** (xv) the feeding of ryegrass forage (hay or haylage) vs. forage of other grass types (e.g. meadow grass or timothy) or no forage. Animals whose turnout area bordered woodland had a reduced rate of laminitis compared to animals not turned out adjacent to woodland.

**Table 1:** Final multivariable Cox regression model for variables associated with active episodes of owner-reported laminitis (*n* = 100) in a cohort of British horses and ponies (*n* = 978 horses/ponies providing 6390 observations and a cumulative time of 974 HYAR after exclusions for missing data)

Variable	No. laminitis episodes	HYAR/100	Hazard ratio	95% CI	Wald P-value	LRS P-value
Animal-level factors						
*Weight change since last entry*						*0.003*
Weight lost/maintained	68	7.6	Ref.			
Weight gained	30	2.0	2.3	1.4, 3.8	< 0.001	
Not specified	2	0.1	1.8	0.4, 8.0	0.44	
*Breed category (including breed crosses)*						*0.007*
Native pony	65	3.8	1.9	1.2, 3.0	0.007	
Other breed	35	5.9	Ref.			
Laminitis history						
*Previous laminitis while with current owner*						*< 0.001*
No	23	6.4	Ref.			
Yes, veterinary-diagnosed	44	2.4	1.7	0.8, 3.8	0.15	
Yes, not veterinary-diagnosed	33	0.9	4.5	2.2, 9.1	< 0.001	
*Time taken to return to soundness following most recent episode*						*0.002*
0 to 2 weeks	38	7.4	Ref.			
More than 2 weeks to 2 months	32	1.3	2.0	1.0, 3.8	0.04	
More than 2 months	30	1.0	3.1	1.6, 6.2	0.001	
Health management and recent health history						
*Currently on steroidal anti-inflammatories*						*0.03*
No	96	9.6	Ref.			
Yes	4	0.1	4.0	1.4, 11.6	0.01	
*Currently on non-steroidal anti-inflammatories*						*< 0.001*
No	77	8.9	Ref.			
Yes	23	0.8	2.9	1.7, 4.8	< 0.001	
*Current lameness*						*0.02*
Other lameness/no lameness	95	9.6	Ref.			
Soft tissue injury (e.g. tendon/ligament)	5	0.2	4.1	1.5, 10.8	0.005	
*Active wormer ingredients in last wormer given*						*0.003*
Macrocyclic lactones	19	2.4	Ref.			
Benzimidazoles	15	0.5	3.8	1.8, 8.1	< 0.001	
Other/no wormer	66	6.8	1.3	0.7, 2.2	0.39	
Hoof care						
*Lameness/soreness following routine hoof care*						*0.004*
Not lame/sore after shoeing/trimming	83	9.3	Ref.			
Lame/sore after shoeing/trimming	17	0.4	2.5	1.4, 4.4	0.002	
*Trimming/shoeing frequency*						*0.03*
Up to 8 weeks	80	8.3	Ref.			
More than 8 weeks	20	1.4	1.9	1.1, 3.2	0.02	
Turnout and grazing management						
*Time of day access to grass currently available*						*0.007*
Morning only	9	0.4	4.3	1.8, 10.8	0.002	
Day and night	28	4.7	Ref.			
Other/grass not available	63	4.7	1.4	0.7, 2.8	0.28	
*Grazing muzzle use*						*0.001*
Whole time while grazing/not used	89	9.5	Ref.			
Part of the time while grazing	11	0.3	3.6	1.8, 7.2	< 0.001	
*Woodland bordering turnout area*						*0.007*
No/entirely stabled	82	7.1	Ref.			
Yes	18	2.6	0.5	0.3, 0.9	0.01	
*Main reason for transport in the previous year*						*0.001*
Other reason/not transported	82	8.7	Ref.			
Moving yards	18	1.0	2.6	1.5, 4.5	< 0.001	
*Average time currently spent stabled*						*< 0.001*
Up to 12 h	46	2.7	3.2	1.7, 5.9	< 0.001	
More than 12 h	19	2.1	Ref.			
Free access/not stabled	35	4.9	1.7	0.8, 3.7	0.15	
Supplementary feeding						
*Ryegrass forage fed*						*< 0.001*
Other/no forage fed	91	9.3	Ref.			
Yes	9	0.5	4.7	2.2, 10.0	< 0.001	

*HYAR* Horse-years at risk, *LRS* Likelihood ratio statistic, *Ref*. Referent group in which the Hazard Ratio = 1.0; 95% CI – 95% Confidence Interval

## Discussion

This is the first web-based cohort study to obtain time to event, multiple-record data regarding horses and ponies, allowing quantification of the relationship between time-varying exposure variables and owner-reported laminitis development. The findings contribute to the improved knowledge of laminitis epidemiology in GB, demonstrating temporal associations between a number of animal- and management-level factors and owner-reported laminitis which included a combination of veterinary-diagnosed and/or owner-recognise episodes.

### Factors with consistent evidence

This study has corroborated the association between laminitis and three risk factors identified in a previous GB-based case-control study of veterinary diagnosed disease [9]: (i) a previous history of laminitis, (ii) lameness or soreness following routine shoeing/trimming and (iii) recent weight gain - demonstrating that each of these were present prior to laminitis development. These factors should be used to guide future recommendations to prevent laminitis.

A history of laminitis has previously been associated with a higher susceptibility to future episodes [9, 13], and although not a modifiable factor it serves as an important risk group identifier. Earlier recognition of laminitis, alongside ensuring animals receive adequate and prompt veterinary attention, farriery support and diagnostic testing of underlying disorders, should promote quicker recovery times and in turn reduce the likelihood of future episodes [27]. The time to return to soundness following the most recent episode, as reported by the owner, was also strongly associated with future laminitis development. The duration and severity of lameness and the degree of rotation of the distal phalanx in chronic episodes [28] have previously been associated with poor clinical outcome and increased risk of euthanasia [29]. Relatively little work has been published regarding laminitis treatment, rehabilitation and prognosis. A better understanding of commonly-used veterinary, farriery and owner interventions, the associations between recovery times and future health, and the effect of these on animal welfare would be of value.

Presence of endocrine disease (including equine metabolic syndrome [EMS] and/or pituitary *pars intermedia* dysfunction [PPID]) has previously been associated with higher laminitis risk [8, 9, 11]. In the current study presence of EMS, but not PPID alone, was strongly associated with a higher rate of laminitis in the interim health model, remaining significant after adjustment for weight gain, but not when a previous history of laminitis was adjusted for. However, the limitations of owner-reported data in the present study and of animals not clinically examined, nor blood sampled, are acknowledged. The occurrence of multiple laminitis episodes is often the first overt indicator of underlying insulin dysregulation (ID) [30]. Knowledge of a previous laminitis history, therefore, likely precedes diagnosis of EMS, and as such may be more predictive of future laminitis development in cohort studies. Encouraging owners to discuss laminitis, particularly when selling or loaning their animals, will ensure that new owners/carers can take adequate precautions to reduce the risk of future disease development. Incorporating diagnostic endocrine testing and radiographic screening into pre-purchase examinations or post-purchase health checks will help new owners and their veterinary surgeons better understand the clinical history of the animal, identifying those that may be at high risk and allowing prioritisation of health care and management.

Lameness or soreness after shoeing/trimming has previously been associated with laminitis [9] and presents another valuable risk group identifier. There is now evidence that discomfort following routine hoof care preceded laminitis development. This may be reflective of animals with existing sub-clinical lamellar alterations, which then develop into acute-phase episodes following altered foot load bearing [31–33]. However, the effect of poor trimming or inadequate shoeing techniques on laminitis development cannot currently be discounted [34, 35]. Trimming or shoeing intervals greater than 8 weeks were additionally associated with higher rates of laminitis. A similar association was identified at the intermediate multivariable modelling stage of farriery-related risk factors and laminitis by Wylie et al. [9] but was not retained in the final multivariable model. The majority of the animals in the current study were trimmed by farriers and there is scope to involve farriers to a greater extent in laminitis research [36, 37]. Active involvement of farriers in future epidemiological studies would further quantify the relationship between hoof care and laminitis, as well as contribute to knowledge of foot characteristics associated with previous laminitis episodes.

This study also corroborates the association between recent owner-reported weight gain and laminitis found by Wylie et al. [9], identifying that animals that gained weight had higher rates of laminitis compared to those that lost or maintained weight within the same time frame. Weight gain, particularly in already obese or metabolically-compromised animals, may result in hyperinsulinaemia and prolonged periods of ID, potentially leading to insulin resistance (IR) and a higher likelihood of subsequent lamellar pathology [11, 38, 39]. Obesity and weight gain must first be recognised as such before intervention strategies can be implemented. In the current study weight gain was likely occurring unintentionally; 95.9% of weight gain observations belonged to animals being fed with the aim of either weight maintenance (67.1%) or reduction (28.8%). Adequate diet and exercise play a vital role in weight management; whether the goal is to maintain or reduce weight [40–43]. Owner compliance is necessary to implement any weight management interventions [42] and owners should be encouraged to follow training programmes and set exercise goals (including structured non-ridden exercise) for their animals, along with implementing dietary changes if animals are obese or have IR. Earlier recognition of weight gain and subsequent initiation of weight loss to counteract it, or ultimately prevention of unintentional weight gain, provides tangible features upon which to focus laminitis preventive efforts. Approximately 46% of the study population regularly used the weight tracker tool, which graphically presented weight change over time and provided a relatively robust measure of weight change. This tool has potential for wider application and has since been made freely available to horse/pony owners.

### Factors with some prior evidence

This study further identified variables with some prior evidence of association, including breed, steroidal anti-inflammatory administration, transport and worming. The modifiable factors amongst these should be the focus of future laminitis studies.

Pony breeds have previously been reported to be at an increased risk of laminitis compared to Quarter Horses and Thoroughbreds [44, 45], and Arab, Cob, native, pony and Welsh breeds and their crosses had higher rates of incident laminitis episodes compared to other/unknown breeds [8, 13]. Previous case-control studies have reported the odds of laminitis to decrease with increasing height [9] and animals < 149 cm being more likely to have laminitis compared to taller animals [10]. In the current study, a breed association was strengthened when native pony breeds (predominantly Welsh, Connemara, New Forest and Shetland ponies and their crosses) were grouped together and compared to all other pony and horse breeds. This indicates that a combination of height and breed is more predictive of laminitis development than either variable on its own, which may explain the inconsistencies in previous studies [6]. It is advisable to target laminitis prevention towards pony breeds via breed associations and pony clubs, although taking care not to suggest that laminitis prevention is unnecessary for non-pony breeds.

The ability of glucocorticoids to contribute to laminitis development has long been debated [11, 46]. A ‘knowledge summary’ of the existing evidence for glucocorticoid-induced laminitis concluded there was little evidence to indicate that therapeutic use of glucocorticoids increased laminitis risk, unless there was existing underlying endocrinopathy or severe systemic disease present [47]. Welsh et al. [8] found that animals receiving prednisolone had significantly higher rates of laminitis in subsequently recorded laminitis episodes, but not initial ones, supporting the theory that horses with multiple laminitis episodes are at higher risk of glucocorticoid-associated laminitis because they are also more likely to have underlying endocrinopathy. Although a relatively small proportion of horses/ponies were reported to receive steroidal anti-inflammatories in the current study, a significant association with laminitis was maintained in the multivariable model, and did not appear to be confounded by the presence of endocrinopathy. The current data were, however, owner-reported and presence of endocrine disorders may have been under-reported. Veterinary surgeons and owners should consider the presence of underlying endocrinopathy before initiating treatment with steroidal anti-inflammatories.

In the current study animals that moved yards within the previous year had higher rates of laminitis development compared to animals transported for other reasons or not at all. So although a transport-related association was identified, it was different to the finding by Wylie et al. [9], where a protective association between transportation in the previous week and laminitis was identified. The negative physical and psychological impact of travel has previously been documented in horses [48, 49], with recent transport being associated with higher odds of colic [50] and respiratory disease such as pleuropneumonia [51]. Animals moving yards were predominantly native ponies (36.6%), younger than 11 years (39.8%) and were rarely transported; with over 90% being transported only a few times per year. It may be that these younger and less travel-experienced animals were more affected by physiological changes associated with travel stress. However, it is currently unclear whether this variable is associated with a change in environment or the act of transportation itself. Further insight is necessary into owners’ reasons for moving yards, and how the temporary changes surrounding transportation, followed by permanent changes in environment, impact laminitis development.

Worming was a previously identified novel factor, and whilst this study did not replicate the finding that horses/ponies wormed less frequently were more likely to have laminitis [9], the use of benzimidazole products at the most recent worming was associated with an increased rate of laminitis. Benzimidazoles are broad spectrum wormers used to control large roundworm (ascarid) infestations and are effective against encysted and adult redworms (cyathostomes/strongyles), large redworms and pinworms [52]. It remains unclear whether the active ingredients of benzimidazoles play a role in laminitis pathophysiology or whether unidentified confounders place animals receiving benzimidazoles at a higher risk of future laminitis development. For example, gastrointestinal disease-induced endotoxaemia has previously been associated with laminitis [53]. The association between worming practices and laminitis should form the basis of future targeted research.

### Novel factors

The remainder of the identified factors are novel, and require further work to explore their relationship with laminitis and their applicability as potential interventions.

The use of non-steroidal anti-inflammatories was associated with higher rates of laminitis. The majority of animals (74.7%) receiving NSAIDs were older than 17 years, with owners reporting that many were on long-term maintenance doses of phenylbutazone to control arthritis-related pain and promote joint mobility. The effect of chronic NSAID usage, and its contribution to laminitis development, should be considered given the ability of NSAIDs to alter faecal microbiota and contribute to gastrointestinal injury in both horses and people [54–56]. Alternatively, animals on maintenance doses of NSAIDs may be less likely to show overt laminitic clinical signs until they have progressed to a certain level of severity, resulting in potential worsening of lamellar damage and susceptibility to future laminitis episodes. Finally, NSAID use may have been a better measure of the compounded effect of increasing age, PPID and the masking of initial clinical signs than these variables on their own.

Non-laminitis-related soft tissue injury was associated with laminitis development. Where details were provided (21 cases), 42.9% were described as ligament and 19.0% as tendon injuries. These animals were commonly on restricted turnout or field rest, with only 19.8% on box rest (defined as confinement to stable/shelter for 24 h per day with no exercise) – a previously identified risk factor for laminitis [9]. Soft tissue injuries were most frequently reported in animals aged between 11 and 15 years (37.8%) and in Thoroughbred types (21.6%). As these types of injuries are mainly linked with activity [57, 58], it may indicate that highly active horses in their early teens may be most affected by management changes associated with rehabilitation and recovery, and represent potential high risk groups. Prolonged periods of inactivity during rehabilitation and lack of dietary adjustment could initiate metabolic and/or circulatory changes which may contribute to higher rates of laminitis in these animals [59, 60].

An association between laminitis and new access, or abrupt changes in access, to grass [9, 10] was not found; however, a number of potential grazing-related interventions were identified. Animals spending relatively short periods of time on grass during morning hours, those spending less time stabled and use of grazing muzzles only part of the time while grazing (compared to when muzzles were used the whole time during grazing or not at all) were associated with higher rates of laminitis. While these grazing-associated variables may be proxy measures for an increased perceived risk due to breed or body condition, animals routinely anticipating short-term, potentially unrestricted access to grass may actively increase their grass intake, taking in substantial amounts of non-structural carbohydrates (NSCs) within a short time. Compensatory eating has previously been demonstrated in ponies anticipating restricted grass access, indicating that reducing the time spent on grass alone became increasingly inefficient at reducing grass intake [61]. Identifying ways to manage grass intake, without compromising the physical and mental well-being of the animal, is important to consider when seeking to prevent laminitis development. However, the relationship between grass intake and laminitis is evidently complex and likely dependent on multiple contributing factors, including the volume, quality and type of grass available.

Multiple environmental factors influence accumulation and storage of NSCs in grass, the rate and storage of which also depend on grass type [62]. Thus the type of grass grazed or fed as hay/haylage has the potential to contribute to laminitis. The feeding of ryegrass hay/haylage was associated with higher rates of laminitis when compared to forage of other types (e.g. meadow grass or timothy) or none. Previous studies have shown that perennial ryegrass contains more rapidly-fermentable NSCs than timothy and ryegrass had the highest percentage of NSC per dry matter relative to 23 other grass species examined, indicating it may not be suitable for animals at risk of laminitis [63, 64].

The only protective factor identified related to animals whose turnout bordered woodland. This association is potentially a proxy measure for another exposure, such as geographical location or pasture characteristics [64], although no associations were found between different pasture and soil types, nor pasture fertilisation, in the current study. Owner-reporting of grass type present on grazing and in some forage being fed (e.g. straw) were generally poorly answered. Better assessment of pasture and forage types, and their relationship with laminitis, could be obtained in future by site visits or submission of soil and grass/forage samples before any further recommendations regarding these factors are developed.

**Limitations**

The main challenges with field-based prospective cohort studies are recruiting and retaining a large and representative cohort [65–67]. The proportions of ponies and animals with a previous history of laminitis appeared greater than those previously reported in equine populations in GB [9, 68–70]. The currently identified risk factors may therefore be more applicable to, and have a higher impact on, pony breeds with a previous history of laminitis. Reliance on owners to recognise laminitis may have led to some misclassification of non-veterinary-attended episodes. However, several precautions such as use of standardised and previously-validated data collection tools [9, 14] that were individually screened, a clear case definition and the longitudinal nature of the study contributed to minimising misclassification bias. The level of detail required for some of the variables (e.g. the types of grass available on grazing or the specific brand and amount of feed fed) were often not well known or difficult to categorise and/or standardise from the information provided, potentially leading to associations between laminitis and dietary factors not being identified in the current study. Better ways of quantifying and categorising dietary variables, rather than relying on owner observations alone, is recommended for future laminitis studies. Although this study has demonstrated a temporal sequence of association for all factors, with exposure preceding outcome, not all participants submitted data as regularly as requested. Some animals, therefore, had longer intervals between records during which it was assumed that exposures remained the same until the subsequent follow-up. This could have affected the temporal association in instances where data from these longer intervals were omitted. Although a number of factors identified here have shown consistency with anecdotal opinions and previous robust epidemiological studies in different populations, it is not straightforward to explain the biological plausibility of all. Some may be proxy measures for other factors not identified by the present study or may reflect our current as-yet incomplete understanding of the complex interaction between management-level factors, an individual’s inherent predisposition and laminitis development.

## Conclusions

This study has contributed to improved knowledge of laminitis epidemiology in GB and has demonstrated a temporal association between a number of animal- and management-level factors and owner-reported laminitis. It has corroborated the association between previously-identified risk factors and laminitis and identified potential management-related interventions and risk group identifiers which should be targeted for future detailed study. There is now consistent evidence that weight gain, a previous history of laminitis, and soreness following routine hoof care are associated with laminitis development, as the same factors were found to be associated with laminitis in a previous case-control study [9]. Priority targeting of the more common exposures that are also most-modifiable, such as weight gain, will have the highest impact on disease incidence. These results serve as a sound evidence-base towards the development of strategic recommendations for the horse/pony-owning population. The creation of evidence-based recommendations, and translation to practical interventions, will contribute to a reduced laminitis rate in GB, while risk groups identifiers will distinguish animals for which the interventions would be of particular relevance.

## Additional files

Additional file 1: Online health and management questionnaire used by participants in the cohort study of equine laminitis in Great Britain. (PDF 1040 kb)

Additional file 2: The monitoring of weight and condition booklet provided to participants in the cohort study of equine laminitis in Great Britain. (PDF 2645 kb)

Additional file 3: Online owner laminitis reporting form used by participants in the cohort study of equine laminitis in Great Britain. (PDF 331 kb)

Additional file 4: A table showing the demographics of the cohort population of horses and ponies at baseline presented in descending order of frequency, unless the variable was ordinal in nature. (DOCX 23 kb)

Additional file 5: A table showing the univariable Cox regression results of variables associated with laminitis (*P* < 0.25) in a cohort study of horses and ponies in Great Britain. (DOCX 108 kb)

Additional file 6: A table showing the multivariable Cox regression interim models of variables associated with laminitis (*n* = 123 episodes) in a cohort study of horses and ponies in Great Britain. (DOCX 37 kb)

## Abbreviations

BCS: Body condition score
BW: Body weight
CI: 95% confidence interval
CNS: Cresty neck score
CS: Cox-Snell
EMS: Equine metabolic syndrome
GB: Great Britain
HYAR: Horse-years at risk
ID: Insulin dysregulation
IQR: Interquartile range
IR: Insulin resistance
LRF: Laminitis reporting form
LRS: Likelihood ratio statistic
LRT: Likelihood ratio test
NSAID: Non-steroidal anti-inflammatory drugs
NSC: Non-structural carbohydrates
PPID: Pituitary *pars intermedia* dysfunction
SADP: Suspensory apparatus of the distal phalanx

## Footnotes

1 Dean, A.G., Sullivan, K.M., Soe, M.M. OpenEpi: Open Source Epidemiologic Statistics for Public Health, Version. www.OpenEpi.com, updated 2013/04/06.

2 Microsoft Corporation, Redmond, Washington, USA.

3 StataCorp. 2017. Stata Statistical Software: Release 15. College Station, TX: StataCorp LLC.
