# Supplementary material for: Identification of modifiable factors associated with owner-reported equine laminitis in Britain using a web-based cohort study approach
Source: BMC Vet Res. 2019 Feb 12;15:59. doi: 10.1186/s12917-019-1798-8 (PMC6373032; doi:10.1186/s12917-019-1798-8)
Supplement: Supplementary file 1 — Online health and management questionnaire used by participants in the cohort study of equine laminitis in Great Britain. (PDF 1040 kb) [file 12917_2019_1798_MOESM1_ESM.pdf]

Baseline Questionnaire

This questionnaire is made up of 8 sections:

Section 1. General information about your horse/pony

Section 2. Turnout and management of grazing

Section 3. Stabling and indoor environment

Section 4. Feeding

Section 5. Exercise

Section 6. Transport

Section 7. Hoof care

Section 8. Health management and recent health history

About completing the questionnaire:

- You are welcome to enrol more than one horse/pony in the study but please complete a separate questionnaire for each animal. If you decide to only enrol one or some but not all of your animals, please choose the one(s) whose name(s) come(s) first in the alphabet to ensure that our data are unbiased.
- For purposes of this questionnaire the term 'horse' refers to both horses and ponies.
- Please complete the details to the best of your knowledge and ability and select the most appropriate response(s) to each and every question.
- Unless asked otherwise, please provide the most CURRENT information (as on the day of completing the questionnaire) when replying to the questions.
- Where required, please include as much relevant detail as possible.
- This questionnaire should take between 15 - 45 minutes to complete.
- All instructions and clarifications are printed in *italics.*

I agree to the use of the collected data for research purposes. I understand that my details will remain confidential and will not be shared with third parties. I give consent for my veterinary practice to be contacted directly with regards to my animal's health. I understand that I can withdraw from the study at any time, but the data submitted up until that point may still be used for research purposes.

Please tick this box if you agree to the above. ☐

The data collected from this questionnaire will be used to help improve the health and welfare of horses and ponies through research. The Animal Health Trust and the Royal Veterinary College will hold any personal information purely for the purposes of research and in accordance with the Data Protection Act 1998. We will not pass this information to any third party.

Section 1: General information about your horse

1.1 What is your horse's name? [ ]

1.2 Do you:

☐ Own the horse
☐ Have the horse on full loan
☐ Other (*please specify*): [ ]

1.3 When did you obtain the horse? *If you are unsure please give the nearest month and/or year you know.* DD / MM / YYYY

1.4 What gender is your horse?

☐ Mare/filly ☐ Gelding ☐ Stallion/colt

1.5 What breed is your horse?

☐ Cross-breed (*please specify if known*): [ ]
☐ Pure breed (*please specify if known*): [ ]
☐ Don't know

1.6 What is your horse's age OR their date of birth (DOB)?
If you are unsure please give approximate age/date.

[ ][ ] years [ ][ ] months **OR** DOB: DD / MM / YYYY

1.7 What height is your horse? *If you are unsure please give the approximate height.*

[ ][ ] hands [ ][ ] inches **OR** [ ][ ][ ] . [ ] cm

1.8 What colour is your horse?

☐ Bay ☐ Black
☐ Chestnut ☐ Coloured
☐ Grey
☐ Other (*please specify*): [ ]

1.9 How much does your horse weigh? *If unsure please give the approximate/estimated weight.*

[ ][ ][ ][ ] kg **OR** [ ][ ][ ][ ] lb

1.10 How did you obtain the weight above?

☐ Estimated by eye
☐ Used a weight tape (a tape on which the weight is already marked)
☐ Used an ordinary tape measure and calculated weight with a formula
☐ Weighed on a weigh bridge in the past moth
☐ Weighed on a weigh bridge more than a month ago
☐ Other (*please specify*): [ ]

1.11 Which of the images below is most representative of your horse’s current shape?

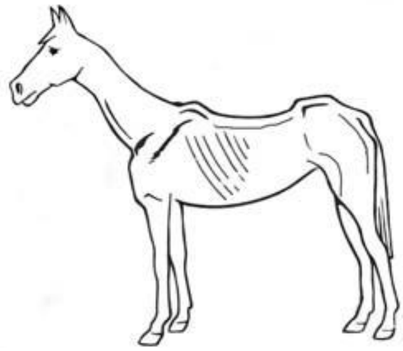

0 ☐

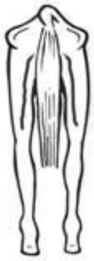

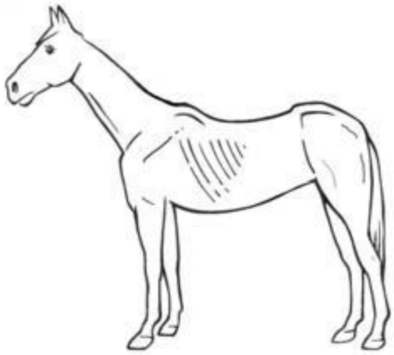

1 ☐

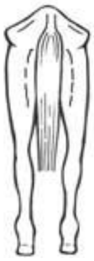

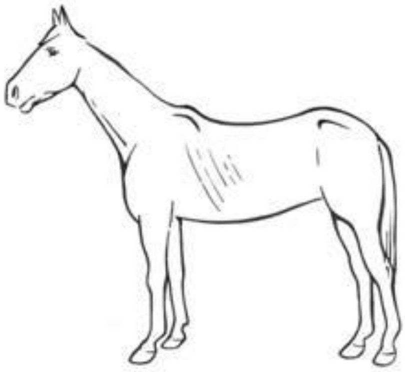

2 ☐

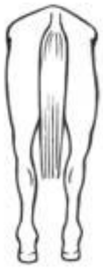

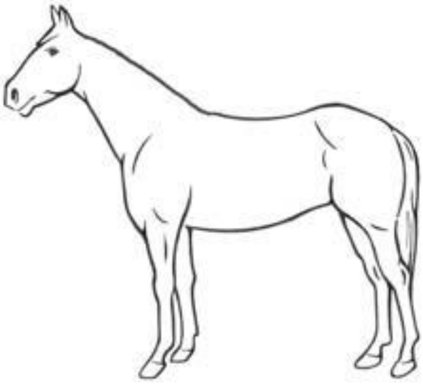

3 ☐

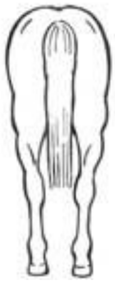

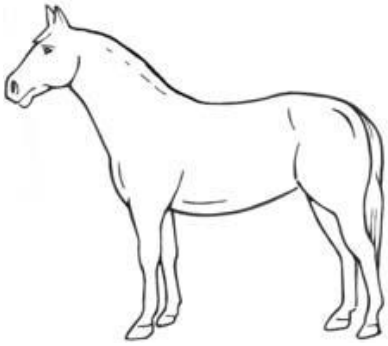

4 ☐

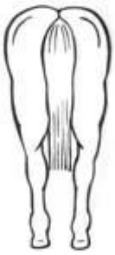

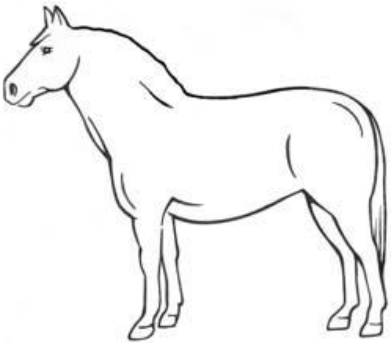

5 ☐

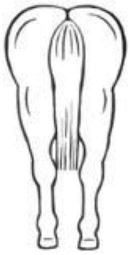

Source: National Equine Welfare Council

1.12 Which image and description below is most representative of your horse's current neck shape?

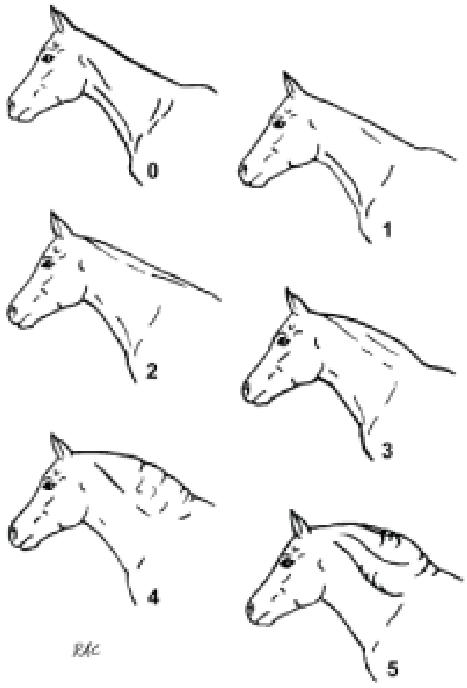

Score	Description
0	No palpable crest.
1	No visual appearance of a crest, but slight filling felt with palpation.
2	Noticeable appearance of a crest, but fat deposited fairly evenly from poll to withers. Crest easily cupped in one hand and bent from side to side.
3	Crest enlarged and thickened, so fat is deposited more heavily in middle of the neck than toward poll and withers, giving a mounded appearance. Crest fills cupped hand and begins losing side to side flexibility.
4	Crest grossly enlarged and thickened, and can no longer be cupped in one hand or easily bent from side to side. Crest may have wrinkles or creases perpendicular to the topline.
5	Crest is so large it permanently droops to one side.

Source: Carter *et al.*, 2009

☐ 0 ☐ 1 ☐ 2 ☐ 3 ☐ 4 ☐ 5

Section 2: Turnout and management of grazing

Please note that turnout refers to any free access to space outside the stable including grass paddock, bare/dirt paddock, surfaced area, etc.

2.1 How is your horse currently kept when not being ridden?

☐ Entirely outdoors
☐ Partly outdoors/partly stabled
☐ Entirely stabled

If your horse does NOT have access to turnout go to Section 3: Stabling and indoor environment.
If your horse does have access to turnout complete the following questions:

2.2 What size turnout does your horse currently have access to? *If you are unsure please give an approximate answer.*

☐☐☐☐ acres **OR** ☐☐☐☐ m^2

2.3 Is your horse turned out individually or with other horses in the same turnout area?
If turned out with others, please pick the most appropriate option below.

☐ Individually
☐ With one other horse
☐ With between 2 - 5 other horses
☐ With more than 5 other horses

2.4 What type of turnout does your horse currently have access to for all or part of the day?
Please indicate all that apply.

☐ Grass paddock
☐ Bare/dirt paddock
☐ Soft surfaced area (e.g. sand school or surfaced ménage)
☐ Hard surfaced area (e.g. courtyard)
☐ Other (*please specify*): ______

2.5 Where does your horse currently spend **MOST** of its turn out time? *Please tick the most appropriate option below.*

☐ Grass paddock
☐ Bare/dirt paddock
☐ Soft surfaced area (e.g. sand school or surfaced ménage)
☐ Hard surfaced area (e.g. courtyard)
☐ Other (*please specify*): ______

2.6 Are droppings removed from your turnout area(s)? *Please indicate the most appropriate option below.*

☐ Yes, at least once per day
☐ Yes, not every day but more often than once a week
☐ Yes, once a week
☐ Yes, once a month or less
☐ No

2.6.1 If yes, how is this done?

☐ Poo picking by hand
☐ Mechanical removal
☐ Other (*please specify*): ______

2.7 What type of land borders with your turnout area? *Please indicate all that apply.*

☐ Grazing pasture
☐ Agricultural crop
☐ Organic agricultural crop
☐ Organic livestock farm
☐ Industrial
☐ Urban/rural housing and roads
☐ Woodland
☐ Other (*please specify*): ______

2.8 Are the following trees or shrubs (or their fruits) found **within reach** of your horse when it is turned out? *If your tree or shrub is not listed, please specify another tree in the option below.*

	Yes	No	Don't know
Oak/acorns 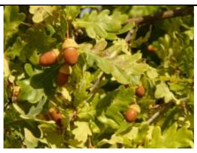	☐	☐	☐
Box Elder/Box Maple 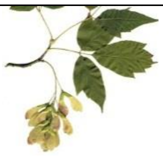	☐	☐	☐
Sycamore 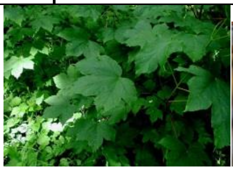 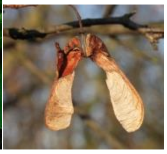	☐	☐	☐
Ivy 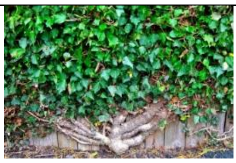	☐	☐	☐
Blackthorn/Sloe berries 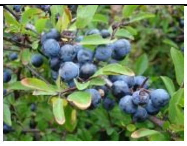	☐	☐	☐
Hawthorn 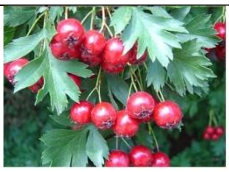	☐	☐	☐
Roses/Rosehips 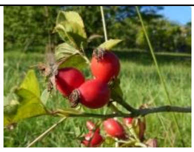	☐	☐	☐
Willow 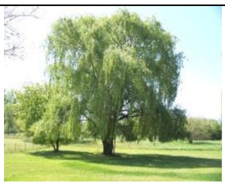	☐	☐	☐
Elm 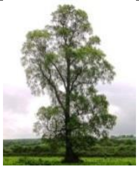	☐	☐	☐
Other, please specify:			

2.9 Does your horse currently have access to **ANY** grass while turned out?

☐ Yes ☐ No

If your horse does <u>NOT</u> have access to any grass while turned out go to Section 3: Stabling and indoor environment.

If your horse does have access to grass while turned out complete the following questions:

2.10 Has your horse been re-introduced to grass within the past month, after previously not having access to grass? ☐ Yes ☐ No

2.10.1 If yes, what was the reason for the horse not having previous access to grass?

☐ On box rest due to illness/injury
☐ Bare winter pasture
☐ Weight-control measure
☐ No turnout available
☐ Other (*please specify*): [ ]

2.10.2 How long was your horse kept completely off grass for?

☐ Up to 1 week
☐ More than 1 and up to 2 weeks
☐ More than 2 and up to 3 weeks
☐ More than 3 and up to 4 weeks
☐ More than 4 weeks
☐ Other (*please specify*): [ ]

2.10.3 How long, out of a 24 hour period, was your horse allowed to access the grass for the first time after being kept off it? *Please select an option to the nearest hour.*

☐ Up to 1 hour
☐ More than 1 and up to 3 hours
☐ More than 3 and up to 6 hours
☐ More than 6 and up to 12 hours
☐ More than 12 and up to 23 hours
☐ 24 hours

2.11 How long, on average, does your horse currently spend at grass every day?

☐ Up to 1 hour
☐ More than 1 and up to 3 hours
☐ More than 3 and up to 6 hours
☐ More than 6 and up to 12 hours
☐ More than 12 and up to 23 hours
☐ 24 hours

2.12 When is your horse currently most likely able to access grass?

☐ Morning only
☐ Afternoon only
☐ Daylight hours only
☐ Night-time hours only
☐ Day and night (24 hours)
☐ Other (*please specify*): [ ]

2.13 Are you currently using any methods to restrict grass intake? *Please indicate all that apply.*

☐ No
☐ Use a grazing muzzle

☐ Strip grazing
☐ Restricted turnout
☐ Other (*please specify*): [ ]

2.13.1 If your horse grazes with a grazing muzzle, please indicate if this is worn:
☐ All the time while gazing
☐ Part of the time while grazing

2.14 Which **ONE** of the following best describes the grazing your horse is currently on?
☐ Meadow pasture (grass and other non-woody plants)
☐ Mature grass paddock (seeded more than 5 years ago)
☐ New grass paddock (seeded within the last 5 years)
☐ Other (*please specify*): [ ]
☐ Don't know

2.15 Please indicate if you know the grass seed type that your horse's grazing mainly consists of? *Please indicate all that apply.*

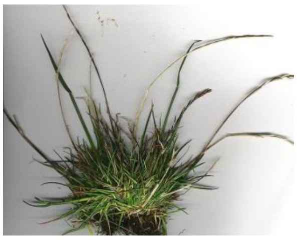
☐Ryegrass

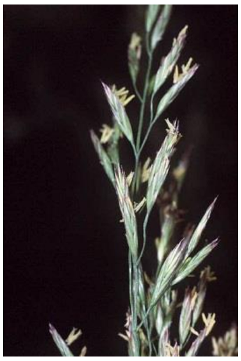
☐Fescue

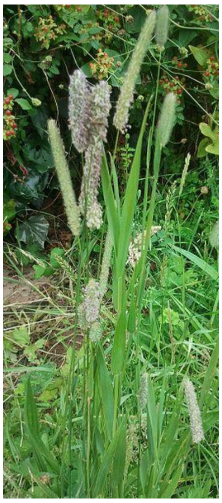
☐Timothy

☐ Other (*please specify*): [ ]
☐ Don't know

2.16 Please estimate the approximate length of the pasture your horse is currently grazing:
☐ Less than 5 cm / less than 2 inches
☐ Between 5 and 15 cm / between 2 and 6 inches
☐ More than 15 and up to 30 cm / more than 6 and up to 12 inches
☐ More than 30 cm / more than 12 inches

2.17 How would you describe the grass cover of the pasture your horse is currently grazing?
☐ Sparse (there are large amounts of bare earth between areas of grass)
☐ Patchy (there are some areas of bare earth between areas of grass)
☐ Adequate (examination at a distance looks good but close inspection reveals some areas of poor cover)
☐ Good (close examination of the paddock confirms good grass cover)

2.18 Are any of these herbaceous plant species currently present in the pasture your horse grazes? *If your herbaceous plant is not listed, please specify another in the option below.*

	Yes	No	Don’t know
Clover 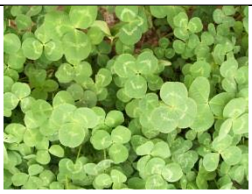	☐	☐	☐
Ragwort 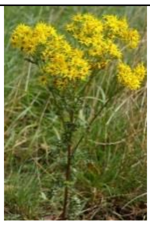	☐	☐	☐
Buttercups 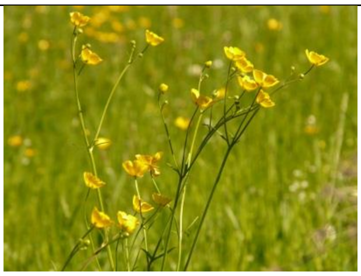	☐	☐	☐
Thistles 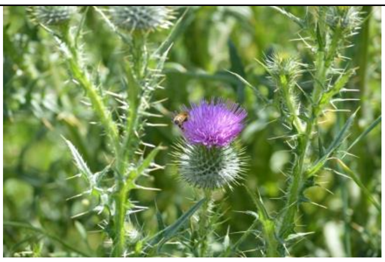	☐	☐	☐
Nettles 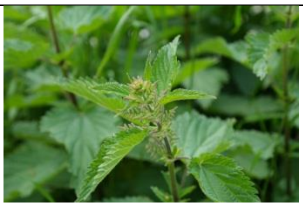	☐	☐	☐
Dandelions 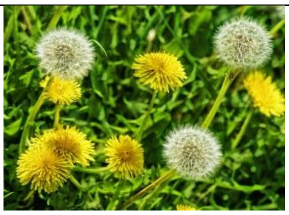	☐	☐	☐
Other, please specify:			

2.19 When was the pasture last fertilised?

- ☐ Never
- ☐ Less than 1 month ago
- ☐ Between 1 and 4 months ago
- ☐ More than 4 months ago
- ☐ Don’t know

2.19.1 If the pasture was fertilised, what type of fertiliser was used?

☐ Nitrogen based
☐ Farmyard manure
☐ Potash based
☐ Poultry manure
☐ Horse manure
☐ Other (*please specify*): ______
☐ Don't know

2.20 How would you describe the type of soil your pasture contains?

☐ Sandy (drains quickly in wet weather but prone to scorching in dry weather)
☐ Loamy (rich dark soil that is generally fertile)
☐ Clay (retains water in very wet weather and prone to becoming boggy)
☐ Other (*please specify*): ______
☐ Don't know

2.21 When was the pasture last cut?

☐ Never
☐ Less than 1 month ago
☐ Between 1 and 6 months ago
☐ More than 6 months ago
☐ Don't know

2.21.1 If the field was cut, what was the reason?

☐ Hay
☐ Topping only
☐ Silage
☐ Other (*please specify*): ______

2.22 Are other domestic animals present on the pasture where your horse grazes?

☐ Yes ☐ No

2.22.1 If yes, please indicate which domestic animals share the grazing space with your horse. *Please indicate all that apply.*

☐ Cattle ☐ Goats
☐ Sheep ☐ Domestic birds
☐ Donkeys ☐ Other (*please specify*): ______

Section 3: Stabling and indoor environment

3.1 Is your horse currently stabled for either all or part of the time?

☐ Yes ☐ No

If your horse is NOT currently stabled at all, go to Section 4: Feeding.
If your horse is stabled for all or part of the time complete the following questions:

3.2 What is your reason for currently stabling your horse for all or part of the time? *Please indicate all that apply.*

☐ Adverse weather
☐ To prevent injury
☐ Reduce grass intake
☐ Personal preference
☐ Preserve/rest grazing land
☐ Yard rules
☐ Other (*please specify*): ________

3.3 How long, on average, does your horse currently spend stabled every day?

☐ Up to 1 hour
☐ More than 1 and up to 3 hours
☐ More than 3 and up to 6 hours
☐ More than 6 and up to 12 hours
☐ More than 12 and up to 23 hours
☐ 24 hours
☐ I am not sure, my horse is able to freely access or leave the stable

3.4 When is your horse currently most likely to be stabled?

☐ Morning only
☐ Afternoon only
☐ Daylight hours only
☐ Night-time hours only
☐ Day and night (24 hours)
☐ Other (*please specify*): ________

3.5 What is the size of the stable your horse is currently housed in?
If unsure, please provide an approximate size.

☐☐ by ☐☐ feet **OR** ☐☐ by ☐☐ metres

3.6 How is your horse currently stabled?

☐ Field shelter
☐ Individual open fronted loosebox/stable (NOT in a barn)
☐ Individual open fronted loosebox/stable (WITHIN a barn)
☐ Communal indoor space/barn without individual stables
☐ Other (*please specify*): ________

3.7 What is the base surface of the stable your horse is currently housed in?

☐ Concrete
☐ Brick
☐ Earth
☐ Don't know
☐ Other (*please specify*): ________

3.8 Do you use rubber matting on the base of your stable?

☐ Yes ☐ No

3.9 Please indicate what type(s) of bedding you currently use. *Please indicate all that apply.*

☐ No bedding
☐ Straw
☐ Shavings
☐ Paper
☐ Pellets
☐ Other (*please specify*): ________

Section 4: Feeding

4.1 What type of forage **(other than grass**, if the horse has access to grazing) does your horse currently receive and when? *Please indicate all that apply. If you know the grass type used to make the forage, please select one of the available options; if you are unsure please select the "Don't know" options.*

Type of forage	Freely available (ad lib)	Once a day	Twice a day	More than twice a day
None ☐				
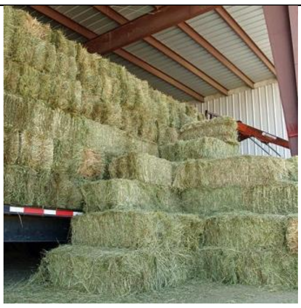 Grass Hay ☐				
a) Meadow grass	☐	☐	☐	☐
b) Ryegrass	☐	☐	☐	☐
c) Timothy	☐	☐	☐	☐
d) Other *(please specify)* ______	☐	☐	☐	☐
e) Don't know ☐	☐	☐	☐	☐
Legume Hay (e.g. alfalfa/Lucerne) ☐ 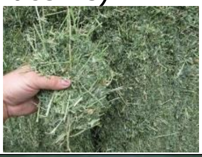	☐	☐	☐	☐
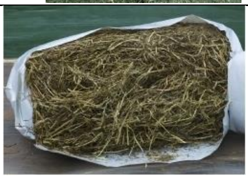 Haylage ☐				
a) Meadow grass	☐	☐	☐	☐
b) Ryegrass	☐	☐	☐	☐
c) Timothy	☐	☐	☐	☐
d) Other *(please specify)* ______	☐	☐	☐	☐
e) Don't know ☐	☐	☐	☐	☐
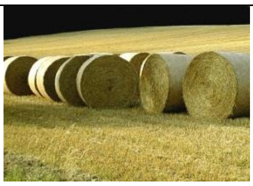 Straw ☐				
a) Barley straw	☐	☐	☐	☐
b) Oat straw	☐	☐	☐	☐
c) Wheat straw	☐	☐	☐	☐
d) Other *(please specify)* ______	☐	☐	☐	☐
e) Don't know ☐	☐	☐	☐	☐
Other, please specify:				

4.1.1 How much forage is currently fed to your horse per day? Please include weight if known (e.g. 5 kg hay if weighed) or approximate amount (e.g. two large haylage nets/ 4 hay slices from a conventional bale). *Please indicate all forages that apply.*

[ ]

4.1.2 If hay is fed, is it currently soaked?

☐ Yes ☐ No ☐ Not applicable, I don't feed hay

4.1.2.1 If yes, what are the reasons for soaking? *Please indicate all that apply.*

☐ Remove soluble sugars
☐ Reduce spores and dust
☐ Horse preference
☐ Reduce calorie intake
☐ Respiratory disease control
☐ Other (*please specify*): [ ]

4.1.2.2 If yes, approximately how long is hay soaked for?

☐ Less than 1 hour
☐ More than 1 and up to 6 hours
☐ More than 6 and up to 9 hours
☐ More than 9 and up to 12 hours
☐ More than 12 hours

4.1.3 How is forage (other than grass) usually offered? *Please indicate all that apply.*

☐ Free from ground
☐ Straight from bale
☐ Regular holed haynet
☐ Small holed haynet/halyage net
☐ Manger (including haybars and crates)
☐ Any type of slowfeeder other than haynet
☐ Other (*please specify*): [ ]

4.2 How frequently does your horse currently get hard feed/concentrates (not forage)?

☐ Never
☐ Once per day
☐ 2 times per day
☐ 3 times per day
☐ More than 3 times per day

4.2.1 Give details of the hard feed/concentrates currently being fed **per meal**. *If your feed is not listed, click on "Add hard feed/concentrate". Please also include any succulents (apples, carrots, parsnips, etc.) here if fed on a daily basis.*

	Brand and type	Approximate amount **per meal** *(weight if known/ type or size of scoop used)*
**e.g. Chaff** ☑<br>**a) Molassed** ☑	**Dengie Alfa A**	**1 Stubbs scoop/meal (approx. 300g)**
Mix ☐		

	Brand and type	Approximate amount **per meal** *(weight if known/ type or size of scoop used)*
a) Low energy ☐		
b) High energy ☐		
Cubes/nuts ☐		
a) Low energy ☐		
b) High energy ☐		
Sugar beet ☐		
a) Molassed ☐		
b) Unmolassed ☐		
Chaff ☐		
a) Molassed ☐		
b) Unmolassed ☐		
Oats ☐		
Barley ☐		
Bran ☐		
Other:		

4.2.2 Was there any over-feeding of hard food in the previous week? *(e.g. accidental access to feed store/unintentional concentrate overload)*

☐ Yes ☐ No ☐ Don't know

4.2.2.1 If yes, please give further details:

4.3 Has there been a change to the quantity of your horse's feed in the past month?

	Not fed/not applicable	No change	Increased amount	Decreased amount
Hard food	☐	☐	☐	☐
Forage	☐	☐	☐	☐

4.4 Which of the following BEST describes your horse in terms of nutritional needs?

☐ Easy keeper/good doer
☐ Hard keeper/poor doer
☐ Somewhere between the two

4.5 Which of the following options would best describe the way that you are currently feeding your horse?

☐ To increase their weight
☐ To maintain their current weight
☐ To decrease their weight

4.5.1 If you are trying to decrease your horse's weight, have you found weight-loss has been:

☐ Very easy to achieve ☐ Moderately easy to achieve
☐ Hard to achieve ☐ Impossible to achieve

4.5.2 Which of the following have you been using to help with the weight-loss?
Please indicate all that apply.

☐ Reduced amount of feed ☐ Switched to lower calorie feed
☐ Increased exercise ☐ Other (*please specify*):

4.6 Is your horse currently fed any supplements (in addition to the hard feeds/concentrates and forage mentioned previously)?

☐ Yes ☐ No

4.6.1 If yes, which general area of supplements do you currently feed? *Please indicate all that apply.*

☐ General Vitamins and Minerals ☐ Salts/Electrolytes
☐ Calmers ☐ Bone and Joint
☐ Muscle ☐ Hoof
☐ Skin ☐ Anti-ulcer
☐ Hormone (e.g. Equine Cushing's/hormonal mares)
☐ Respiratory ☐ Gut balancer (including pre- and/or probiotics)
☐ Feed balancer ☐ Anti-Laminitic
☐ Other (*please specify*): ☐

4.6.2 Give details of the supplements you are currently feeding? *If unsure how long you have been feeding the supplement, please give the approximate time.*

Product name	Times fed/day	Time of day fed *(indicate all that apply)*	Are you feeding "according to the manufacturer's instructions" per day?	How long have you been using this product?
**e.g. NAF Biotin**	**☐ less than once**<br>**☐ once**<br>**☑ twice**<br>**☐ three times**<br>**☐ More than three times**	**☑ morning**<br>**☐ afternoon**<br>**☑evening**	**☐yes**<br>**☐less**<br>**☑more**	**☐☐weeks**<br>**0 2months**<br>**☐☐years**
☐	☐ less than once<br>☐ once<br>☐ twice<br>☐ three times<br>☐ More than three times	☐ morning<br>☐ afternoon<br>☐ evening	☐ yes<br>☐ less<br>☐more	☐☐weeks<br>☐☐months<br>☐☐years
☐	☐ less than once<br>☐ once<br>☐ twice<br>☐ three times<br>☐ More than three times	☐ morning<br>☐ afternoon<br>☐ evening	☐ yes<br>☐ less<br>☐more	☐☐weeks<br>☐☐months<br>☐☐years

Section 5: Exercise

5.1 Which of the following options best describes your horse's **usual use**? *Please indicate all that apply.*

☐ Hacking ☐ Schooling
☐ Mainly unaffiliated competitions ☐ Mainly affiliated competitions
☐ Professional sport competing (training and competing in racing, eventing, endurance, showjumping, dressage, etc.)
☐ Professional showing ☐ Not ridden
☐ Breeding/stud ☐ Other (*please specify*): ☐

If your horse is NOT ridden, answer the following questions and then proceed to Section 6: Transport

5.1.1 If your horse is currently NOT ridden, what is the reason for this? *Please indicate all that apply.*

☐ Healthy but lack of rider or time
☐ Healthy but not yet backed
☐ Retired due to age
☐ Retired due to recurrent injury/illness
☐ Other (*please specify*):

5.1.2 If your horse is NOT ridden, do you try to actively encourage movement? *Please indicate all that apply.*

☐ No
☐ Yes, by placing food and water far apart to encourage walking
☐ Yes, by in-hand exercise (e.g. lungeing, long-reining, walking in-hand)
☐ Yes, by exercise on a horse-walker
☐ Yes, other (*please specify*):

If your horse is RIDDEN, please answer these questions:

5.2 Please indicate which of these activities your horse participates in while **NOT COMPETING**. *Please indicate all that apply.*

Discipline	Days per week	Days per month	Hours per day
Cross-country jump			
Dressage or flatwork schooling			
Endurance training			
Hacking			
Hunting			
In-hand schooling or long-reining			
Lungeing			
Race training: flat			
Race training: jumps			
Showjumping training			
Other:			
Other:			

5.3 Please indicate which of these activities your horse participates in **COMPETITIVELY**, also including the general level of competitions you participate in. *Please indicate all that apply.*

Discipline	Days per week	Days per month	Level
N/A, my horse doesn't compete ☐			
**e.g. Dressage**		**1**	**Novice**
Cross-country jumping			
Dressage			
Endurance			
Eventing			
Racing: flat			
Racing: jumps			
Showjumping			
Showing (in-hand)			
Showing (ridden)			
Other:			
Other:			

5.4 How would you currently rate your horse's level of fitness?

☐ Very unfit ☐ Unfit ☐ Fit ☐ Very fit

5.5 Considering your horse's general workload, is your horse currently working:

☐ More than usual ☐ About the same as usual
☐ Less than usual ☐ Off for a period of ☐☐☐ days

5.6 Which **ONE** of the following surfaces do you mainly school/train your horse on?

☐ Grass ☐ Woodchip
☐ Rubber ☐ Sand
☐ Sand mixed with fibre and/or rubber
☐ Other (*please specify*): ________
☐ Not applicable, I don't school/train my horse

5.7 Which **ONE** of the following surfaces do you mainly hack on?

☐ Off-road tracks (including grass) ☐ Roads
☐ An approximately equal combination of off-road hacking (including grass) and roads
☐ Other (*please specify*): ________
☐ Not applicable, I don't hack

Section 6: Transport

6.1 Has your horse been transported in the past year?

☐ Yes ☐ No

If NO go to Section 7: Hoof care.
If YES complete the following questions:

6.1.1. On average, how often do you transport your horse?

☐ More than once a week
☐ Once a week
☐ A few times a month
☐ Once a month
☐ A few times a year

6.1.2 What are the main reasons for transporting your horse? *Please indicate all that apply.*

☐ Competitions
☐ Lessons/clinics
☐ Hacking somewhere other than yard surroundings
☐ Long distance/pleasure rides
☐ Veterinary treatment
☐ Moving yards
☐ Other (*please specify*): ______

6.1.3 When was your horse last transported?

D D / M M / Y Y Y Y

6.1.4 How was your horse transported?

☐ Trailer (single)
☐ Trailer (double)
☐ Horsebox (up to 2 horse capacity)
☐ Lorry (more than 2 horse capacity)
☐ Other (*please specify*): ______

6.1.5 What was the duration of your last journey (one way)?
If you are unsure please give an approximate time.

☐☐ hours ☐☐ minutes

6.1.6 What was the distance of your last journey (one way)? *If you are unsure please give an approximate distance.*

☐☐ miles **OR** ☐☐ km

6.1.7 What direction was your horse facing during your last journey?

☐ Forwards ↑

☐ Backwards ↓

☐ Sideways 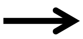

☐ Herringbone facing forwards 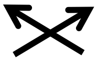

☐ Herringbone facing backwards 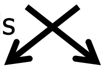

6.1.8 What was the reason for your last journey with your horse?
- ☐ Competition
- ☐ Lesson/clinic
- ☐ Hacking somewhere other than yard surroundings
- ☐ Long distance/pleasure ride
- ☐ Veterinary treatment
- ☐ Recent purchase
- ☐ Moving yards
- ☐ Other (*please specify*): ____________________

6.1.9 How would you best describe your last journey with your horse?
- ☐ Mostly along straight A roads and/or motorways
- ☐ Mostly along windy country lanes and/or roads with many roundabouts
- ☐ An approximately equal mix of the two options above
- ☐ Other (*please specify*): ____________________

6.1.10 How did you feel your horse travelled on your last journey?
- ☐ Well (travelled quietly and was calm upon arrival)
- ☐ Poorly (noisy traveller, sweaty and nervous upon arrival)
- ☐ Other (*please specify*): ____________________

Section 7: Hoof care

7.1 How would you rate the general quality of your horse’s hooves?

☐ Poor ☐ Average ☐ Good

7.2 Which of these diagrams would **BEST DESCRIBE** the current **shape and angles** of each of your horse’s hooves?

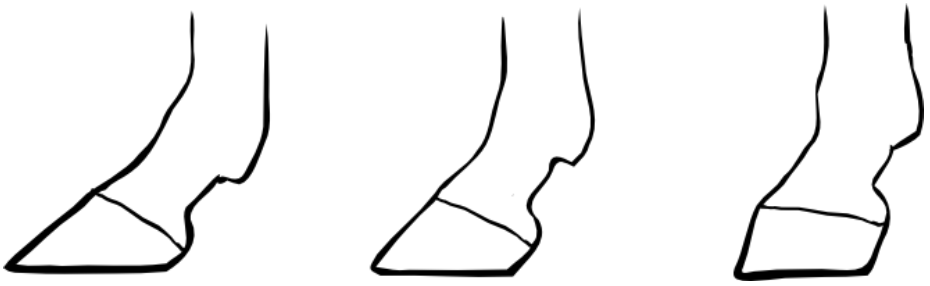

Right front	☐	☐	☐
Left front	☐	☐	☐
Right hind	☐	☐	☐
Left hind	☐	☐	☐

7.3 Does your horse currently have any of the hoof conditions listed in the table below?
Hover over the names to see picture examples of each hoof condition.

Hoof condition	Yes	No
Toe cracks 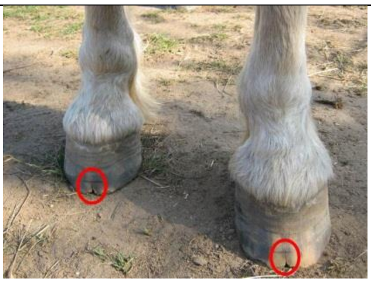	☐	☐
Heel cracks 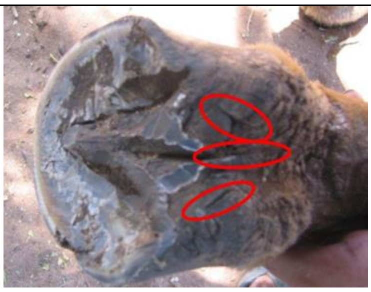 *Image courtesy of Dr J. Ireland*	☐	☐
White line disease / Seedy toe 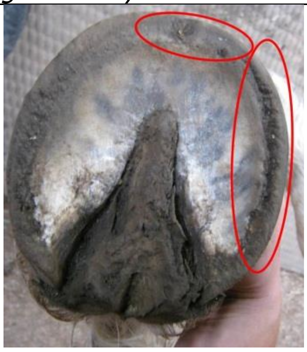	☐	☐
Thrush 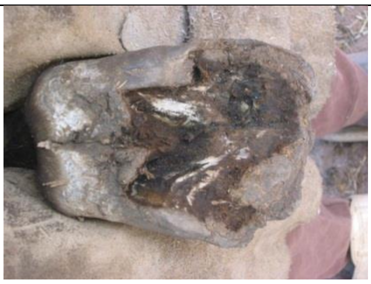 *Image courtesy of Dr J. Ireland*	☐	☐
Soft, crumbling hooves 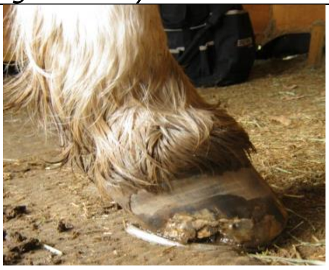	☐	☐

7.4 Do you currently use any topical hoof care products? (e.g. hoof oil/ointment)
☐ Yes ☐ No

7.4.1 If yes, what type of topical hoof care products do you currently use? *Please indicate all that apply.*

☐ General hoof oil ☐ General hoof ointment
☐ Hoof hardener ☐ Hoof moisturiser
☐ Other (*please specify*): [ ]

7.4.2 Please indicate the name and brand of the topical hoof care product(s) above and how often you use it/apply it. *If you do not know how long you have been using the product(s), please give an approximate time.*

Product name and brand	Approximately how often is the product applied?	How long have you been using this product?
**e.g. Kevin Bacon's hoof dressing**	☐ **more than once a day**<br>☐ **once a day**<br>☑ **a few times a week**<br>☐ **a few times a month**	[ ][ ] **weeks**<br>[0][6] **months**<br>[ ][ ] **years**
[ ]	☐ more than once a day<br>☐ once a day<br>☐ a few times a week<br>☐ a few times a month	[ ][ ] weeks<br>[ ][ ] months<br>[ ][ ] years
[ ]	☐ more than once a day<br>☐ once a day<br>☐ a few times a week<br>☐ a few times a month	[ ][ ] weeks<br>[ ][ ] months<br>[ ][ ] years

7.4.3 Why do you use the topical hoof care product(s) stated above?

[ ]

7.5 Do you currently use hoof boots?

☐ Yes ☐ No

7.5.1 If yes, why? [ ]

7.5.2 If yes, do you use them on:

☐ Front hooves only ☐ Back hooves only ☐ Both front and back hooves

7.5.3 If yes, when do you use hoof boots?

☐ Only when I ride my horse
☐ When I ride and turn my horse out
☐ Only when I turn my horse out
☐ Other (*please specify*): [ ]

7.5.4 What type of hoof boots do you usually use? *Please enter name below*

[ ]

7.5.5 Do you currently use studs with your hoof boots?

☐ Yes ☐ No

7.6 Is your horse currently shod?

☐ Yes ☐ No

If your horse is currently <u>NOT</u> shod, proceed to question 7.7
If your horse is currently shod, answer the questions below:

7.6.1 How is your horse currently shod?

☐ Shod on all four feet ☐ Shod front feet only ☐ Shod back feet only

7.6.2 What type of shoeing do you usually have carried out?

☐ Hot shoeing ☐ Cold shoeing ☐ Don't know

7.6.3 How often, on average, is the horse reshod?

Every ☐☐ weeks

7.6.4 When was your horse last shod? *If unsure please give approximate date.*

D D / M M / Y Y Y Y

7.6.5 Was your horse lame or foot-sore after the most recent episode of shoeing?

☐ Yes ☐ No

7.6.5.1 If yes, did your regular farrier perform the shoeing during this most recent episode?

☐ Yes ☐ No

7.6.5.2 Did the farrier perform a different trim to normal during this most recent episode?

☐ Yes ☐ No ☐ Don't know

7.6.5.2.1 If yes, was this was due to:

☐ Hoof damage after losing shoe
☐ Poor condition of hoof
☐ No growth since last shoeing
☐ Other (*please specify*):

7.6.5.3 Did your farrier think that this lameness or foot-soreness was associated with the development of laminitis?

☐ Yes ☐ No ☐ Don't know

7.6.5.4 Has your horse previously been lame or foot-sore following shoeing?

☐ Yes ☐ No

7.6.6 What type(s) of shoes is your horse currently wearing?

	Normal/regular shoes	Glue-on shoes	Egg bar shoes	Heart bar shoes	None	Other(*please specify*)
Front feet	☐	☐	☐	☐	☐	
Hind feet	☐	☐	☐	☐	☐	

7.6.7 Do you currently use studs with your shoes?

☐ Yes ☐ No

7.6.8 Did your farrier change the type of shoes since the previous shoeing?

☐ Yes ☐ No

7.6.8.1 If yes, what was the reason for changing the type of shoes?

[ ]

7.6.9 Do you feel that your horse generally retains shoes:

☐ Very well ☐ Well
☐ Poorly ☐ Very poorly

If your horse is currently shod, proceed to Section 8.
If your horse is currently NOT shod, answer the questions below:

7.7 Who currently trims your horse's hooves? *From now on this person will be referred to as "the trimmer".*

☐ Farrier ☐ Barefoot trimmer
☐ Veterinary surgeon ☐ No-one
☐ Other (*please specify*): [ ]

7.8 How often, on average, are your horse's hooves trimmed?

Every [ ][ ] weeks

7.9 When were your horse's hooves last trimmed?

D D / M M / Y Y Y Y

7.10 Was your horse lame or foot-sore after the most recent episode of trimming?

☐ Yes ☐ No

7.10.1 If yes, did your regular trimmer perform the most recent trim?

☐ Yes ☐ No

7.10.2 Did the trimmer perform a different trim to normal during this episode?

☐ Yes ☐ No ☐ Don't know

7.10.2.1 If yes, was this due to:

☐ Hoof damage
☐ Poor condition of hoof
☐ No growth since last trim
☐ Other (*please specify*): [ ]

7.10.3 Has your horse previously been lame or foot-sore following trimming?

☐ Yes ☐ No

7.10.4 Did the trimmer think that this lameness or foot-soreness was associated with the development of laminitis?

☐ Yes ☐ No ☐ Don't know

7.11 How long has your horse been barefoot?

☐☐ days ☐☐ weeks ☐☐ years

Section 8: Health management and recent health history

Behaviour and general health

8.1 Does your horse exhibit any stereotypical behaviours?

☐ Yes ☐ No ☐ Don't know

8.1.1 If yes, which one(s)? *Please indicate all that apply.*

☐ Box walking ☐ Cribbing
☐ Wind sucking ☐ Weaving
☐ Other (*please specify*): ☐

8.2 Please indicate if your horse is currently vaccinated against: *Please indicate all that apply.*

Vaccination		Date of last vaccination
Not applicable/not vaccinated	☐	
Influenza/'flu'	☐	DD / MM / YYYY
Tetanus	☐	DD / MM / YYYY
Equine Herpes Virus (EHV)	☐	DD / MM / YYYY
Equine Viral Arteritis (EVA)	☐	DD / MM / YYYY
Strangles	☐	DD / MM / YYYY
Other(*please specify*): ☐	☐	DD / MM / YYYY

8.3 How often do you have your horse's teeth checked?

☐ Every 6 months ☐ Once a year
☐ Less than once a year ☐ Only when I think there is a problem
☐ Other (*please specify*): ☐
☐ Never

8.4 Which **ONE** of the following best describes how your horse is wormed?

☐ My horse is never wormed
☐ Wormed according to a set yard schedule
☐ Wormed according to Faecal Egg Count (FEC) results
☐ I set my own worming schedule according to the time of year
☐ Wormed only when I think my horse has worms
☐ Other (*please specify*): ☐

If your horse is <u>NOT</u> wormed and a <u>MARE</u>, proceed to question 8.5 'Foaling/Breeding history'
If your horse is <u>NOT</u> wormed and a <u>GELDING</u>, <u>STALLION</u> or <u>COLT</u>, proceed to question 8.8 'Laminitis history'
If your horse is wormed, answer the questions below:

8.4.1 When was the last time your horse was wormed?
If unsure please give the nearest month and/or year you know.
DD/MM/YYYY

8.4.2 What was the horse wormed with on this occasion?

- ☐ Equest
- ☐ Equest Pramox
- ☐ Equitape
- ☐ Eqvalan
- ☐ Eqvalan Duo
- ☐ Equimax
- ☐ Eraquell
- ☐ Noromectin
- ☐ Panacur
- ☐ Panacur 5 Day Guard
- ☐ Pyratape-P Paste
- ☐ Strongid-P
- ☐ Telmin
- ☐ Vectin Gel
- ☐ Multiworm
- ☐ Verm-X
- ☐ Other (*please specify*): ______

8.4.3 Do you use a Faecal Egg Count (FEC) to help you decide when to worm?
☐ Yes ☐ No

If yes, proceed to question 8.4.3.1a and skip question 8.4.3.1b. If no, proceed to question 8.4.3.1b.

8.4.3.1a If yes, how often do you use a FEC?
- ☐ Once a year
- ☐ Twice a year
- ☐ Three times a year
- ☐ Four times a year
- ☐ Five times a year
- ☐ More than five times a year

8.4.3.2a When was the last time you had the FEC done?
If unsure please give the nearest month and/or year you know.
DD/MM/YYYY

8.4.3.3a What were the results of your last FEC?
- ☐ Low
- ☐ Moderate **OR** ______ eggs per gram *(if known)*
- ☐ High

8.4.3.1b If no, how would you best describe the details of your annual worming programme (when and why you worm)?

______

8.4.4 Do you check for tapeworms using a tapeworm antibody blood test/ELISA?

☐ Yes ☐ No

8.4.4.1 If yes, how often do you use a tapeworm blood test?

☐ Once a year
☐ Twice a year
☐ More than twice a year

8.4.4.2 If yes, when was the last time you had the tapeworm blood test done? *If unsure please give the nearest date you know.*

DD/ MM/ YYYY

8.4.4.3 What were the results of your last tapeworm blood test?

☐ Low
☐ Moderate **OR** ______ *(if known)*
☐ High

8.4.5 Under your current worming programme, how often on average do you worm per year?

☐ Once a year
☐ Twice a year
☐ Three times a year
☐ Four times a year
☐ Five times a year
☐ More than five times a year

8.4.6 Do you make an effort to alternate wormers – for example using wormers with different chemicals to worm for tapeworm each time rather than using the same wormer?

☐ Yes, I make an effort to use different wormers
☐ Sometimes
☐ No, I usually use the same ones

If your horse is a <u>GELDING</u>, <u>STALLION</u> or <u>COLT</u>, go to the next section on 'Laminitis history'
If your horse is a <u>MARE</u>, complete the following questions:

Foaling/breeding history

8.5 Has your mare ever been in foal?

☐ Yes ☐ No ☐ Don't know ☐ Not applicable, my horse is not a mare

8.5.1 If yes, how many foals are you aware she has had?

☐☐ foals

8.5.2 If yes, please indicate when the most recent foaling was: *Please give the nearest month and/or year or leave blank if you do not know.*

DD/ MM/ YYYY

8.6 Is your mare currently in foal?

☐ Yes ☐ No ☐ Don't know

8.6.1 If yes, please indicate when the foal is due. *If you are unsure please give the nearest month and/or year you know.*

MM / YYYY

8.7 To your knowledge, has your mare ever had any reproductive problems?

☐ Yes ☐ No

8.7.1 If yes, please indicate which reproductive problems your mare has had. *Please indicate all that apply.*

☐ Retained placenta
☐ Endometritis
☐ Abortion
☐ Other (*please specify*): ______
☐ Don't know

8.7.1.1 If your mare has previously aborted, what was the cause of the abortion?

☐ Twisted umbilical cord
☐ Equine Herpes Virus (EHV)-1
☐ Placentitis
☐ Twins
☐ Other (*please specify*): ______
☐ Don't know

Laminitis history

When answering the questions in this section please note that 'previous laminitic episodes' referred to below do NOT include a potentially current episode.

8.8 Are you aware of any previous history of laminitis in your horse, BEFORE you took over their care?

☐ Yes ☐ No

8.8.1 If yes, how did you obtain the information about their previous history of laminitis?

☐ I knew the horse before I took over their care
☐ From the previous owner
☐ From veterinary records
☐ From the farrier/trimmer
☐ Other (*please specify*): ______

8.9 Since you have OWNED/TAKEN OVER CARE for your horse, have they had any previous episodes of laminitis?

☐ Yes ☐ No

8.9.1 If yes, do you know the date the last episode started/was diagnosed?

DD / MM / YYYY

8.9.2 Please indicate approximately how many previous episodes your horse had since you have OWNED/TAKEN OVER CARE for them:

☐☐ episodes

8.9.3 How many of these previous episodes of laminitis were diagnosed by a vet?

☐☐ episodes

8.9.4 Who diagnosed this last episode of laminitis? *Please indicate all that apply.*

☐ Vet ☐ Farrier
☐ Barefoot trimmer ☐ Me
☐ Other (*please specify*): ☐
☐ Don't know

8.9.5 Did your horse become fully sound after the last episode of laminitis?

☐ Yes ☐ No, my horse is still not fully sound

8.9.5.1 If yes, approximately how long did it take your horse to become fully sound?

☐☐ weeks ☐☐ months

8.9.6 If your horse was in work prior to the episode of laminitis, did they return to the same level of work after the last episode of laminitis?

☐ Yes
☐ No, my horse has not returned to the same level of work
☐ Not applicable, the horse was not in work before

8.9.6.1 If yes, approximately how long did it take your horse to return to the pre-laminitis level of work?

☐☐ weeks ☐☐ months

8.7 Does your horse currently have laminitis?

☐ Yes ☐ No ☐ Don't know

If yes, please complete and submit the laminitis reporting form if you have not already done so.

General lameness (excluding laminitis)

This section is for any other lameness, excluding that associated with laminitis and covered by the Laminitis Reporting Form.

8.8 Is your horse currently lame?

☐ Yes ☐ No

If your horse is NOT lame, go to the section on 'Disease management' below.
If your horse is lame complete the following questions.

8.8.1 What is the diagnosis/cause of the lameness?

Cause of lameness (*Please indicate all that apply)*	Diagnosed by vet?
☐ Arthritis/osteoarthritis	☐ Yes ☐ No
☐ Bruised sole	☐ Yes ☐ No
☐ Foot abscess	☐ Yes ☐ No
☐ Navicular disease	☐ Yes ☐ No
☐ Soft tissue injury (tendon/ligament)	☐ Yes ☐ No

☐ Fracture/break<br>☐ Lameness of undetermined cause<br>☐ Other (*please specify*): ____<br>☐ Don't know	☐ Yes ☐ No<br>☐ Yes ☐ No<br>☐ Yes ☐ No

8.8.2 Which legs are affected? *Please indicate all that apply.*

☐ Left fore ☐ Right fore
☐ Left hind ☐ Right hind
☐ Don't know

8.8.3 Is your horse able to bear weight on the affected limb(s)?

☐ Yes ☐ No

Disease management

You may require your vet's help in order to complete some of the questions in this section.

8.9 Has your horse had surgery in the past month?

☐ Yes ☐ No

8.9.1 If yes, please indicate the reason for the surgery:

☐ Colic surgery ☐ Castration
☐ Teeth extraction ☐ Sarcoid removal
☐ Other (*please specify*): ____

8.10 Has your horse received general anaesthesia in the past month?

☐ Yes ☐ No

8.11 Is your horse currently being confined to his stable/shelter for 24 hours per day with no exercise (referred to from now as box rest)?

☐ Yes ☐ No

8.11.1 If yes, how long has your horse been on box rest?

☐☐ days **OR** ☐☐ months

8.11.2 How long is your horse required to be on box rest in total?

☐☐ days **OR** ☐☐ months

8.11.3 Please indicate the reason for the box rest:

☐ Illness ☐ Lameness as described above
☐ Suspected laminitis ☐ Confirmed laminitis
☐ Other (*please specify*): ____

8.11.4 Have you noticed a change in your horse's behaviour while on box rest?

☐ Yes ☐ No

8.11.4.1 If yes, how has your horse's behaviour changed? *Please give details below.*

[ ]

8.11.5 Does your horse seem stressed while on box rest?

☐ Yes ☐ No

8.11.6 Is your horse able to see/interact with other horses while on box rest?

☐ No
☐ Yes, but only some of the time
☐ Yes, most of the time

Current health

8.12 Does your horse have any of the following conditions? *Please indicate all that apply.*

Condition	Diagnosed by vet?
☐ Strangles	☐ Yes ☐ No
☐ Heaves/COPD/RAO*	☐ Yes ☐ No
☐ Colic	☐ Yes ☐ No
☐ Equine Gastric Ulcer	☐ Yes ☐ No
☐ Diarrhoea	☐ Yes ☐ No
☐ Equine Grass Sickness	☐ Yes ☐ No
☐ Abnormal oestrous cycles	☐ Yes ☐ No
☐ Testicular problems	☐ Yes ☐ No
☐ Equine Cushing's Disease/PPID*	☐ Yes ☐ No
☐ Equine Metabolic Syndrome/Insulin resistance	☐ Yes ☐ No
☐ Sarcoids	☐ Yes ☐ No
☐ Sweet itch	☐ Yes ☐ No
☐ Mud fever	☐ Yes ☐ No
☐ Tying up/azoturia	☐ Yes ☐ No

**COPD = Chronic Obstructive Pulmonary Disorder, RAO = Recurrent Airway Obstruction*
PPID = Pituitary Pars Intermedia Dysfunction

8.13 To your knowledge, has your horse ever been blood tested for Equine Cushing's Disease/Pituitary *Pars Intermedia* Dysfunction (PPID)?

☐ Yes ☐ No ☐ Don't know

8.13.1 If yes, please indicate when: *If you are unsure please leave blank.*

D D / M M / Y Y Y Y

8.13.2 What type of blood test was used? *Please indicate all that apply.*

☐ Resting Adrenocorticotropic Hormone (ACTH) level
☐ Thyrotropin stimulating hormone (TRH) stimulation test
☐ Dexamethasone suppression test
☐ Other (*please specify*): [ ]
☐ Don't know

8.13.3 Who was the test carried out and interpreted by?

☐ My own vet/vet practice
☐ Other vet/vet practice (e.g. emergency or referral vet)

☐ Other (*please specify*): ______
☐ Don't know

8.13.4 If you remember the results, please indicate them below:

______

8.13.5 What was the reason for suspecting your horse had Equine Cushing's Disease? *Please indicate all that apply.*

☐ Recurrent laminitis
☐ Increasing age
☐ Long curly coat even in summer
☐ Excessive drinking and urination
☐ Sudden change in metabolism (from being a good-doer to poor-doer)
☐ Fatty deposits in certain areas like above the eyes, on the crest and rump
☐ Lethargy
☐ Excessive sweating
☐ Muscle wastage
☐ Other (*please specify*): ______

8.14 To your knowledge, has your horse ever been blood tested for Equine Metabolic Syndrome (EMS)/Insulin Resistance (IR)?

☐ Yes ☐ No ☐ Don't know

8.14.1 If yes, please indicate when: *If you are unsure please leave blank.*

DD / MM / YYYY

8.14.2 What type of blood test was used? *Please indicate all that apply.*

☐ Resting insulin levels
☐ Resting glucose levels
☐ Combined glucose-insulin test (CGIT)
☐ Oral glucose tolerance post-feeding insulin test
☐ Other (*please specify*): ______
☐ Don't know

8.14.3 Who was the test carried out and interpreted by?

☐ My own vet/vet practice
☐ Other vet/vet practice (e.g. emergency or referral vet)
☐ Other (*please specify*): ______
☐ Don't know

8.14.4 If you remember the results, please indicate them below:

______

8.14.5 What was the reason for suspecting your horse had EMS/IR?
Please indicate all that apply.

☐ Breed type
☐ Recurrent laminitis
☐ General obesity or history of obesity
☐ Fatty deposits in certain areas like above the eyes, on the crest and rump
☐ Other (*please specify*): ____________

Medications

8.15 Has your horse received sedation in the past month?

☐ Yes ☐ No

8.15.1 If yes, what was the reason for sedation?

☐ For routine hoof care
☐ For routine teeth care
☐ For clipping
☐ Other (*please specify*): ____________

8.15.2 If known, please indicate what sedative was used:

____

8.16 Is your horse currently receiving any medication prescribed by the vet (excluding wormers, sedatives and vaccinations)?

☐ Yes ☐ No

If your horse is NOT currently receiving any medications go to the 'Comments' section.
If your horse is currently receiving medication(s) complete the following questions:

8.17 Which of the following type(s) of medication is your horse currently receiving? *Please indicate all that apply.*

☐ Oral anti-inflammatory/painkiller
☐ Oral antibiotic
☐ Oral respiratory medication
☐ Oral hormone regulators
☐ Injected anti-inflammatory/painkiller
☐ Injected antibiotic
☐ Topical cream or shampoo

8.18 Give details of the medication that you have mentioned above.

Name/type of medication if known (*Please enter one drug per row*)	When did you start using the medication?	How long should the medication be given for in total?	If feeding orally, approximately how many tablets or sachets/day?
**Example:**<br>**Bute (Phenylbutazone)**	**20/12/2013**	**5 days**	**1 sachet/day**
	DD / MM / YYYY	days	
	DD / MM / YYYY	days	

Comments

This space is for you to make any additional comments that you may have on the questionnaire, this study or specific issues you feel are important. Your input is welcomed.

Thank you for taking the time to fill in this questionnaire!
Your help is greatly appreciated.
