# Supplementary material for: Identification of modifiable factors associated with owner-reported equine laminitis in Britain using a web-based cohort study approach
Source: BMC Vet Res. 2019 Feb 12;15:59. doi: 10.1186/s12917-019-1798-8 (PMC6373032; doi:10.1186/s12917-019-1798-8)
Supplement: Supplementary file 2 — The monitoring of weight and condition booklet provided to participants in the cohort study of equine laminitis in Great Britain. (PDF 2645 kb) [file 12917_2019_1798_MOESM2_ESM.pdf]

Monitoring the weight and condition of your horse/pony

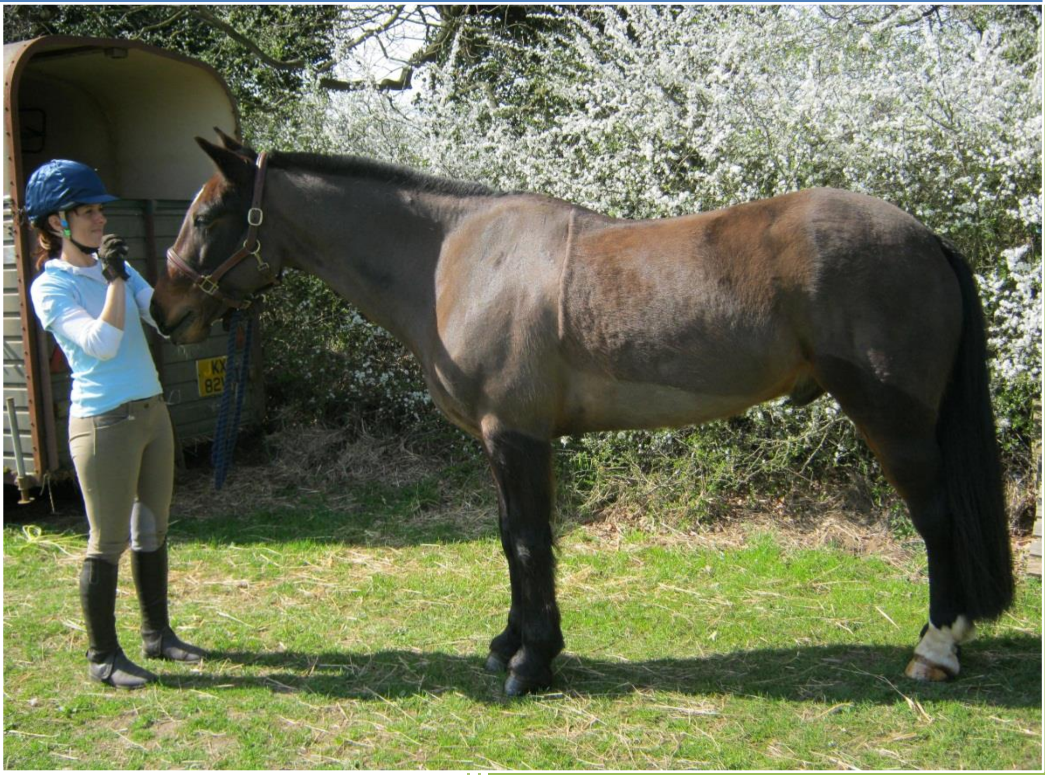

CARE about Laminitis project

www.careaboutlaminitis.org.uk

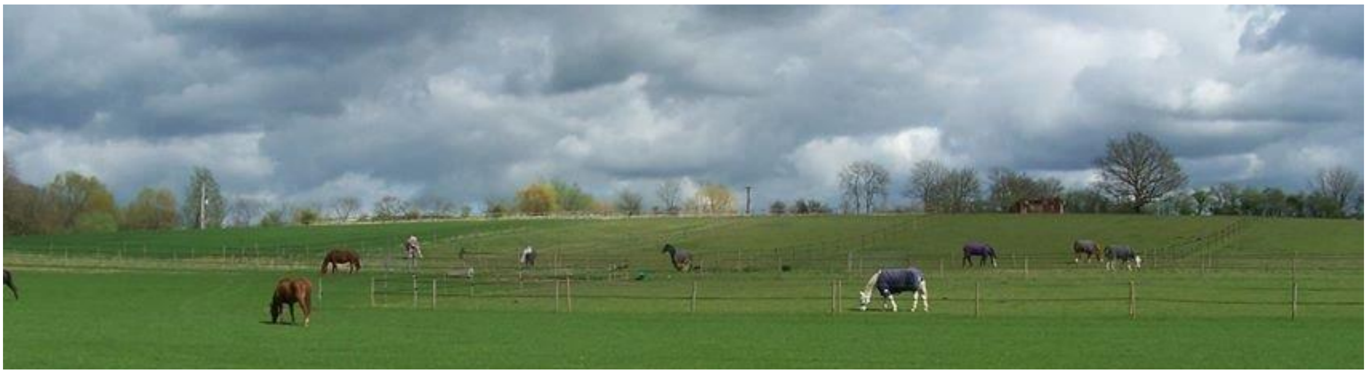

We would like to thank World Horse Welfare for funding the CARE about Laminitis project and for helping put together these guidelines. If you are interested in the work it does to help improve equine welfare worldwide, please visit the website at www.worldhorsewelfare.org

World Horse Welfare
Anne Colvin House
Snetterton
Norfolk
NR16 2LR
Registered charity no: 206658 **and** SC038384

CARE about Laminitis is a collaborative project between the Animal Health Trust (AHT) and the Royal Veterinary College (RVC), with ongoing support from Rossdales Equine Hospital.

If you would like more information on the work the AHT undertakes to fight disease and injury in animals, please visit the website at www.aht.org.uk

Animal Health Trust
Lanwades Park
Kentford
Newmarket
Suffolk CB8 7UU
Registered Charity No: 209642

If you are interested in the work that the RVC does or the study options offered, please visit the website at www.rvc.ac.uk

The Royal Veterinary College
Royal College Street
London
NW1 0TU

If you would like to see the services that Rossdales Equine Hospital offers, please visit the website at www.rossdales.com

Rossdales Equine Hospital
Cotton End Road
Exning
Newmarket
Suffolk
CB8 7NN

Contents

General instructions……………………………………………………………………Pg 3

Body condition scoring and cresty neck scoring…………………………………..Pg 4

How to measure your horse/pony to estimate their weight………………………..Pg 8

How to take your horse/pony's neck measurements……………………………….Pg 12

Recording chart……………………………………………………………………...Pg 14

All these instructions are also available online at www.careaboutlaminitis.org.uk

While you can use the recording chart on page 14 to keep temporary records, please don't forget to input all final values online on the Weight Tracker page of the website.

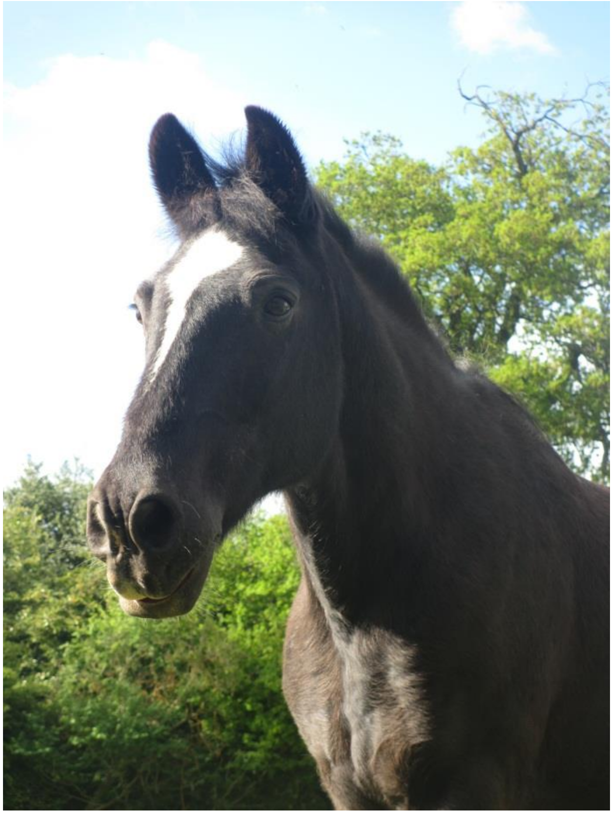

General Instructions

There are some important rules that should be followed when taking measurements and scoring your horse/pony.

Decide on a set time (both time of day and in relation to exercise & feeding) – ideally when your horse/pony is relaxed and comfortable – and make each subsequent recording at approximately the same time. Their weight can fluctuate significantly over a 24 hour period and ensuring you take the measurements at a similar time will mean you are comparing like for like.

You should always take these steps when preparing to measure/score:

Step 1 – Find a level, firm surface for your horse/pony to stand on. Concrete is preferable, but level dirt or short grass would be fine if concrete is not at hand.

Step 2 – Position your horse/pony so that they are standing square (with their front and hind feet together) and in a straight line. Also ensure they are bearing weight on all four feet (a resting back leg will affect the measurements!).

Follow these basic rules:

- same person taking measurements/doing scoring
- same method of measuring/scoring used
- same place
- same time

Consistency in measurements/scoring will ensure the accuracy of weight monitoring.

Taking body and neck measurements

You will have received a CARE tape measure in your welcome pack. It will have centimetres (cm) printed on one side and inches on the other. Please decide whether you will take your measurements in cm or inches and stick to the same unit of measurement for all future recordings.

What you will need to take measurements

- CARE tape measure
- Pen (the one in your welcome pack might come in handy!)
- Recording chart (fancy that, you got one of these too, look at the back of this booklet!)
- A helper to hold your horse/pony and hold one end of the tape measure (sorry, we couldn't provide this!)

Make sure your horse/pony is OK with the tape measure being placed on them. Start by letting them sniff it and if they do appear unsure, take time to get them used to it.

Royal Veterinary College University of London | World Horse Welfare | AHT Animal Health Trust

Body Condition Scoring and Cresty Neck Scoring

In this study, we will be using the Carroll and Huntingdon (1988) 5-point scale to estimate a Body Condition Score (BCS) and a 5-point scale developed by Carter *et al*. (2009) to estimate a Cresty Neck Score (CNS).

While your horse/pony's actual weight gives us an objective method of assessing the "condition" of the horse, weight alone does not discriminate between muscle and fat. As in humans, a heavily muscled athlete would be considered "overweight" or "obese" on a regular Body Mass Index (BMI) scale – in the same category as a person/horse that was truly overweight. That is why assessing a condition score, along with a weight estimation measurement, will provide us with a better idea of the actual shape your horse or pony is in. Body Condition Scoring is also sometimes referred to as fat scoring. We team up with World Horse Welfare and find out how to assess our horses/ponies using the 5-point BCS scale.

We are going to use an averaging system designed by Dr Teresa Hollands to estimate the BCS. You can't effectively estimate a BCS just by looking – you need to feel for the fat cover in certain areas.

Steps 1 & 2 - follow the steps from **pg 3**. Also review the **general instructions** and rules.

Step 3 - Picture an imaginary line dividing your horse/pony into three sections:

> **Section 1: Neck and shoulder**
> *The cut-off point between sections 1 and 2 would be the withers*
>
> **Section 2: Middle** (Back and belly)
> *The cut-off point between sections 2 and 3 would be the last rib*
>
> **Section 3: Bottom** (Pelvis to tail)

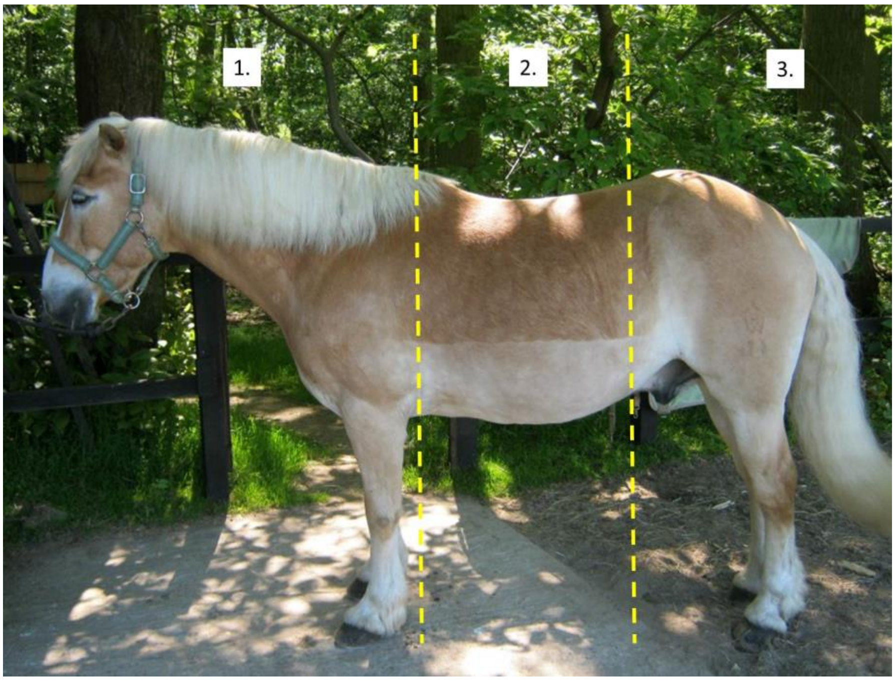

Referring to the images and descriptions in the Body Condition Score guide on page 6, you will need to give your horse/pony one score out of five for their **neck and shoulder (section 1)**, one for their **middle (section 2)** and one for their **bottom (section 3)**, then take an average (add all three scores together and divide by 3). Horses store their weight in different areas, so an average of these scores will produce a more accurate assessment. In the BCS guide, the text that refers to each section has been colour-coded appropriately.

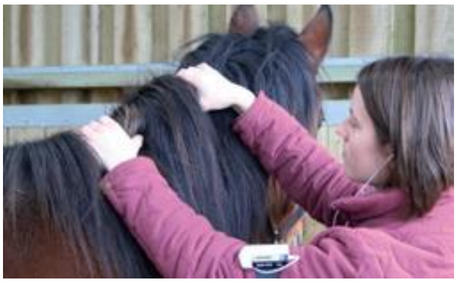

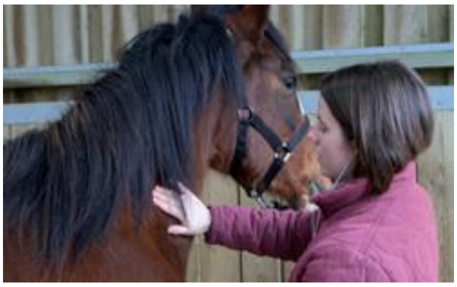

Section 1: Neck and shoulder

- Feel along the top of your horse/pony's neck – can you wobble the top of it (fat), or is it firm (muscle)? Also feel if it is significantly thicker as you move down from the poll towards the withers.
- Run your hand down your horse/pony's neck and onto its shoulder. If fat has built up in front of his shoulder blades, your hand will run from the neck to the shoulder without the shoulder blade 'stopping' your hand. Native breeds often store excess fat in 'pads' behind their shoulders, so feel for this. Look at the BCS guide on the next page and score them out of five for their neck and shoulder.

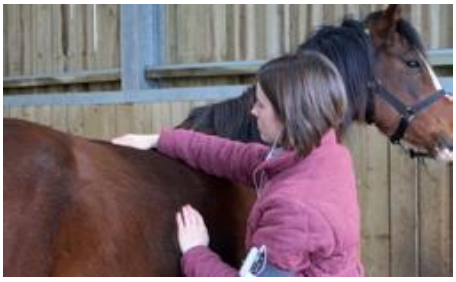

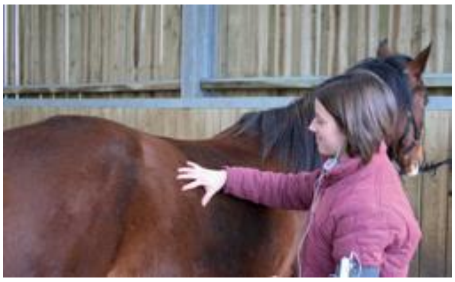

Section 2: Middle (Back and belly)

- Lay your hand across their back. Ideally, your hand should arch over the spine. When horses/ponies put weight on in this area, the fat builds up on either side of the spine, giving you a flat hand.
- Run your hand along their side. You should be able to feel their ribs fairly easily. Horses don't store much fat on their underbellies, so don't use this area to assess their weight. Again, give them a score out of five for their back and middle by referring to the BCS guide on the next page.

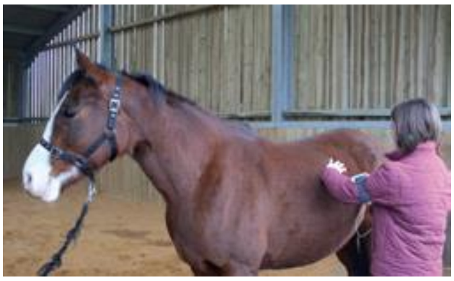

Section 3: Bottom (Pelvis to tail)

Look at your horse/pony from behind – safely. Their bottom should have a rounded curve. Feel for fat pads at the base of the tail. Give them a score out of five for their bottom.

Once you have all three scores for each section, take an average to get the overall BCS score. So for example – if you score 4 for **section 1**, 3 for **section 2** and 4 for **section 3**, then your final average score would be (4 + 3 + 4)/ 3 = 3.6. Use the chart at the back of this booklet (pg 14) to keep a record.

Body Condition Score Guide

0

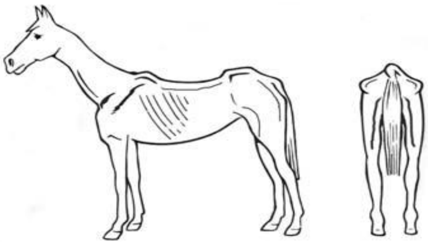

Marked 'ewe' neck, narrow and slack at base. Skin tight over the ribs, which are clearly visible. Spinous processes sharp and easily seen. Angular pelvis, skin tight, very sunken rump. Deep cavity under tail and either side of croup.

1

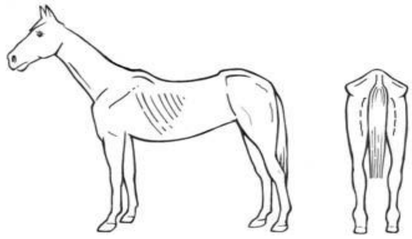

'Ewe' neck, narrow and slack at base. Ribs clearly visible. Skin clearly shrunken either side of spine. Spinous processes well defined. Rump sunken but skin supple, pelvis and croup well defined, cavity under tail.

2

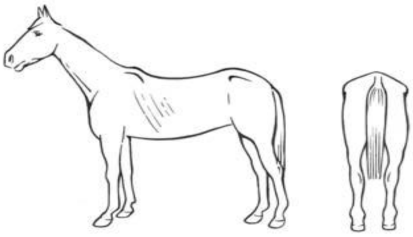

Neck narrow but firm, shoulder blade clearly defined. Ribs just visible. Spine well covered. Spinous processes felt but not seen. Rump flat either side of spine, croup well defined, some fat, slight cavity under tail.

3

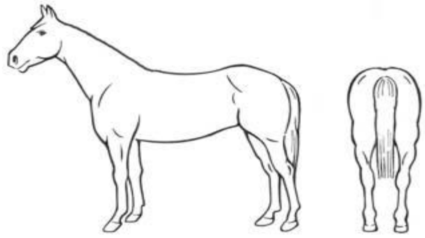

Firm neck, no crest (except stallions), shoulder blades defined. Ribs just covered, easily felt. No gutter along back. Spinous processes covered, but can be felt. Pelvis covered by fat and rounded, no gutter, pelvis easily felt.

4

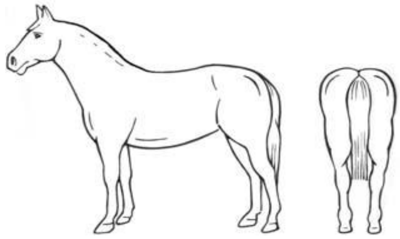

Slight crest on neck, wide and firm. Ribs well covered. Gutter along spine to root of tail. Fat stored either side of the spine to form slight 'apple bottom', with a gutter down the middle. Pelvis covered, felt only with firm pressure.

5

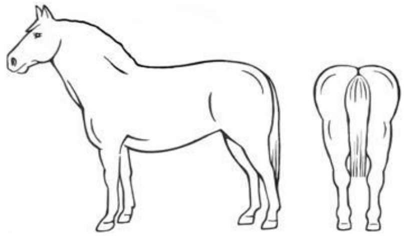

Marked crest, very wide and firm, creases of fat. Shoulder blade buried and difficult to feel. Ribs buried, cannot be felt. Deep gutter along spine, back broad and flat. Deep gutter to root of tail, producing marked 'apple bottom', skin distended. Pelvis buried, cannot be felt.

Images obtained with kind permission from the National Equine Welfare Council.

In a similar way in which we assessed the score for the neck and shoulder in **section 1** of the BCS, we will also give our horse/pony a Cresty Neck Score (CNS) based on the images and descriptions below:

Score	Description
**0**	No palpable crest.
**1**	No visual appearance of a crest, but slight filling felt with palpation.
**2**	Noticeable appearance of a crest, but fat deposited fairly evenly from poll to withers. Crest easily cupped in one hand and bent from side to side.
**3**	Crest enlarged and thickened, so fat is deposited more heavily in middle of the neck than towards poll and withers, giving a mounded appearance. Crest fills cupped hand and begins losing side to side flexibility.
**4**	Crest grossly enlarged and thickened, and can no longer be cupped in one hand or easily bent from side to side. Crest may have wrinkles or creases perpendicular to the topline.
**5**	Crest is so large it permanently droops to one side.

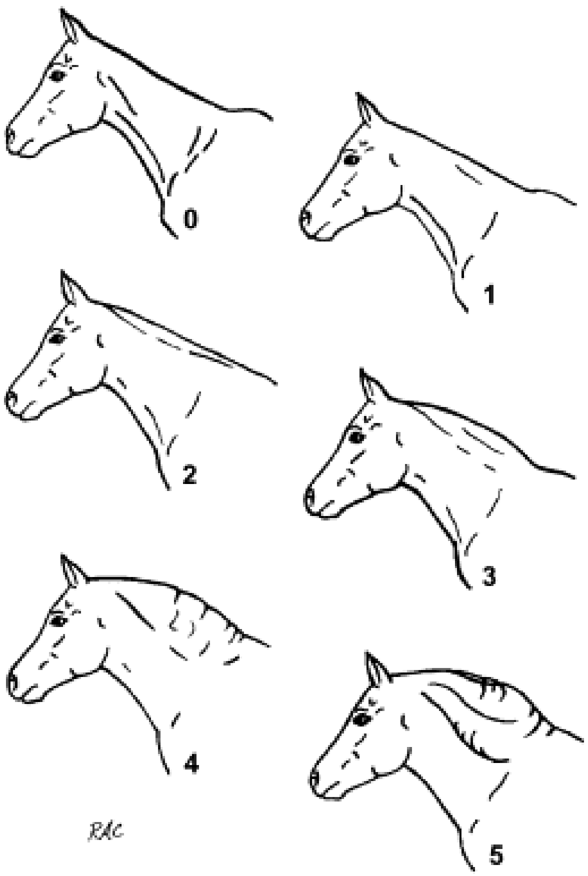

Source: Carter *et al*., 2009

Use the chart at the back of this booklet (pg 14) to keep a record. You will need both the BCS and the CNS to complete your monthly questionnaire follow-ups which can be found online at www.careaboutlaminitis.org.uk.

References

Carroll, C. L. and Huntington, P. J. 1988. Body condition scoring and weight estimation of horses. *Equine Veterinary Journal*, **20**: 41-45.

Carter, R. A., Geor, R. J., Burton Staniar, W., Cubitt, T. A. and Harris, P. A. 2009. Apparent adiposity assessed by standardised scoring systems and morphometric measurements in horses and ponies. *The Veterinary Journal*, **179**: 204 – 210.

How to measure your horse/pony to estimate their weight

We will be using a method of heart girth and body length measurements to estimate our animal's weight. This is because this method of estimating weight, in absence of a weighbridge to give us an exact weight, has been found to be more accurate when compared to using a weight tape method alone (Wagner & Tyler, 2011).

Steps 1 & 2 - Follow the steps on **pg 3**. Also review the **general instructions** and rules.

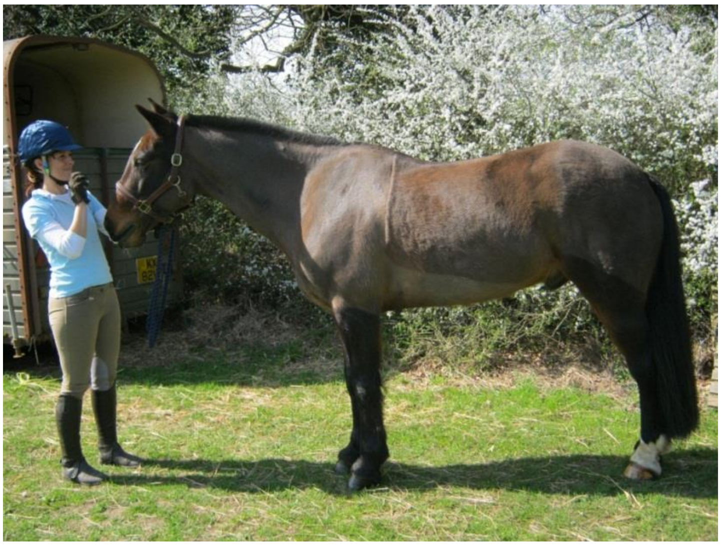

Left: *Our model, Murphy, demonstrates the correct body position when measurements are being taken.*

Step 3 - To take the body length measurement, measure from the **point of shoulder** to the **point of buttock**. To find the point of shoulder, place the palm of your hand at the top of the shoulder (starting nearest the neck) and run your hand down the shoulder blade until you reach a 'bony point'. This is the point of the shoulder. If you are having trouble locating the point of shoulder, decide on a point in this general area and always take measurements from this point.

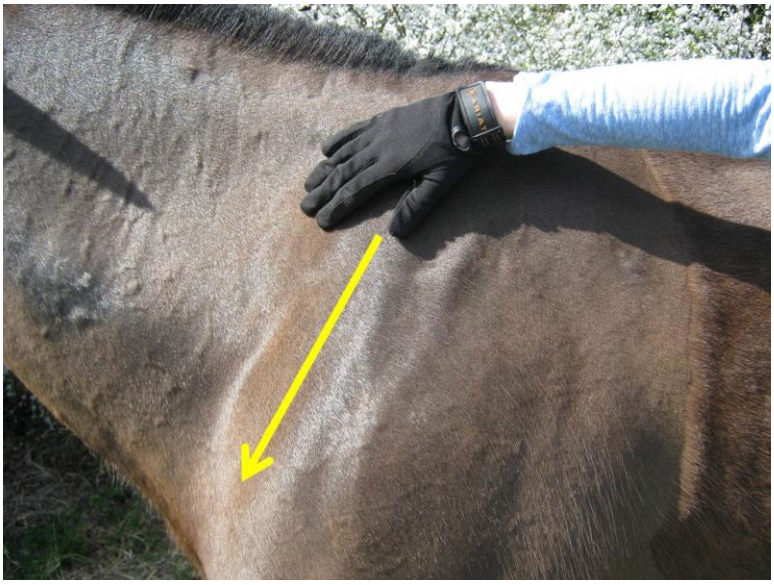

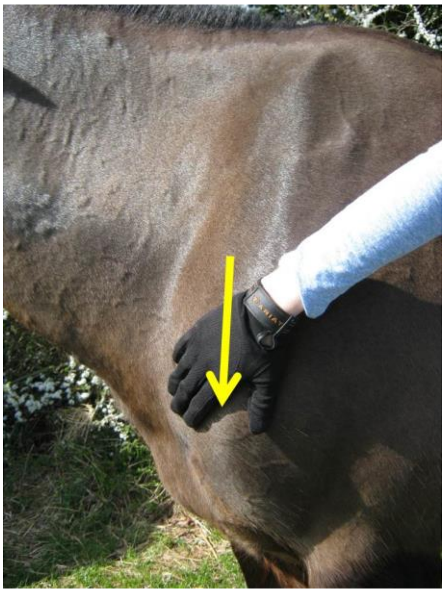

Above left: *Follow the line of the shoulder blade with the flat of your hand.*

Above right: *Run the palm of your hand down the shoulder blade until you reach a "point" after which the shoulder slopes downward.*

Step 4 - The **point of buttock** can be found a few centimetres away from the tail (when you are looking at your horse/pony from behind) and can be described as the “most pointy” part of the buttock (make sure you follow all safety precautions when handling your horse/pony’s rear end). It may be a little tricky to find! If you are having trouble locating the point of buttock, decide on a point in this general area and always take measurements from this point.

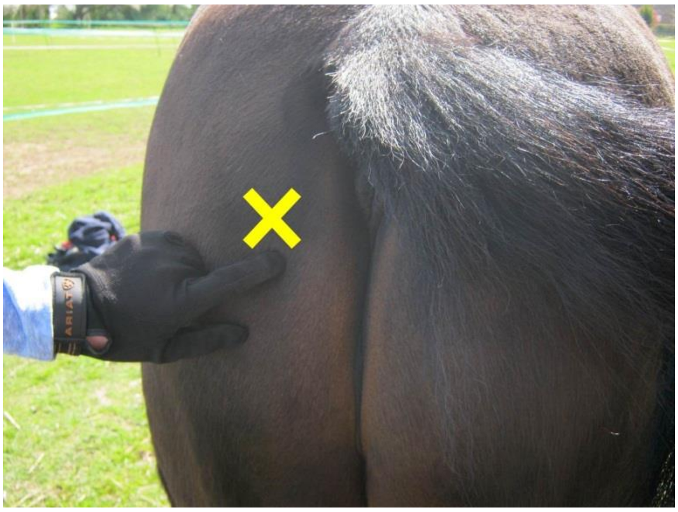

***Left:** The point of buttock should be in this area.*

Step 5 - Ask your helper to hold your horse/pony and to secure one end of the tape at the point of shoulder. Extend the tape around to the point of buttock, following the curve of the bottom. Make sure the tape is kept taut and is not twisted. Record your measurement.

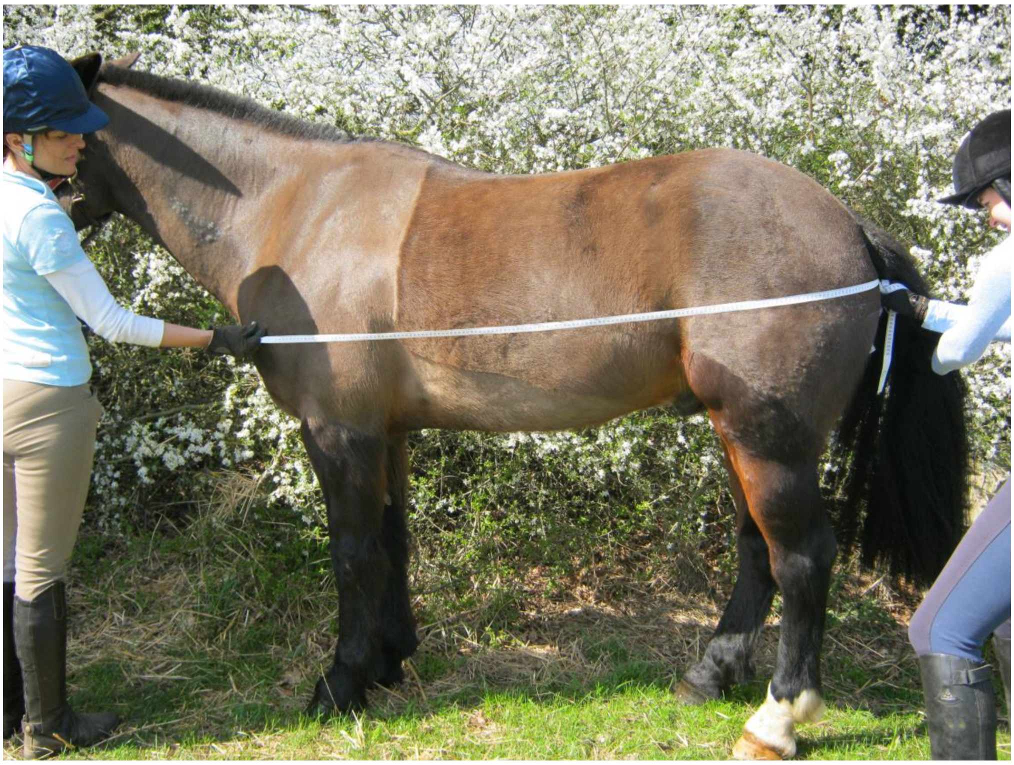

***Above:** Harriett has the tape measure at the point of buttock, with Lyndsay holding it secure at the point of shoulder. When you take the measurement, your tape measure should be at a slight angle, from the point of shoulder up to the point of buttock.*

Step 6 **-** To take the **heart girth measurement**, place your tape measure around the girth of the horse. Ensure the tape measure is not twisted. You want to position the tape measure at the lowest point of the withers (just before they slope into the back) and as close behind the elbow as you can (**NOT** as you would place a girth).

Your tape measure should be on a slight diagonal angle. Once again, make sure this is the case on both sides. Pull the tape measure snugly (especially if you have a furry mammoth!) but not so tight that it indents the skin. Line the tape measure up, ensuring your hand is not placed underneath it, wait for the horse/pony to breathe out (exhale) and record the measurement.

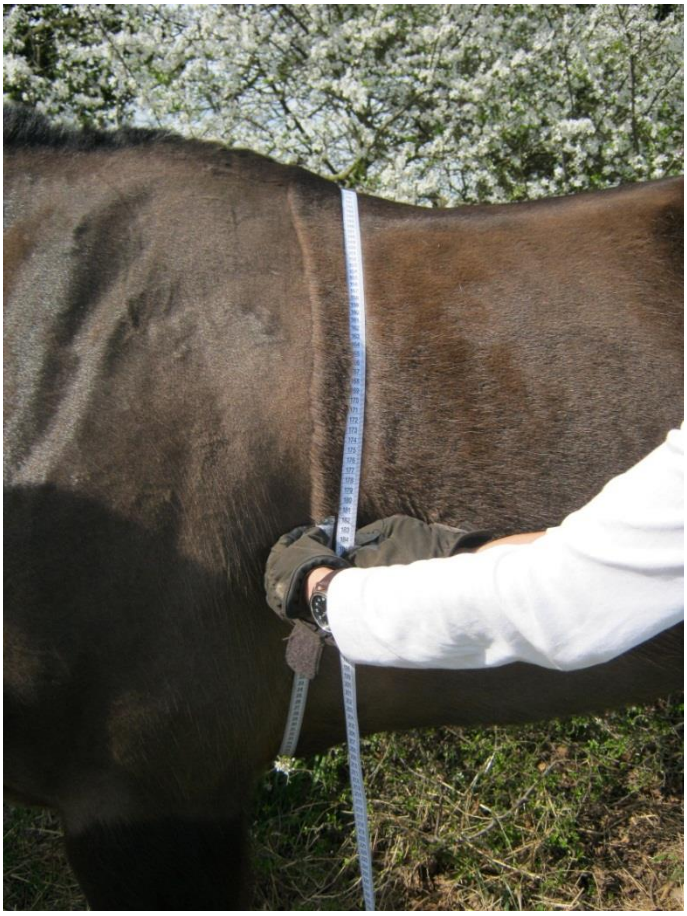

Left: *Pull the tape measure snuggly and line it up to take your reading. Be aware of not placing your hand under the tape measure when you are taking the reading!*

The most important thing to remember is to **be consistent**! Same person, taking the same measurements, at the same times, in the same place! Use the chart at the back of this booklet (pg 14) to keep a record.

Below: *Just to recap, and put the point of shoulder, point of buttock and heart girth into perspective.*

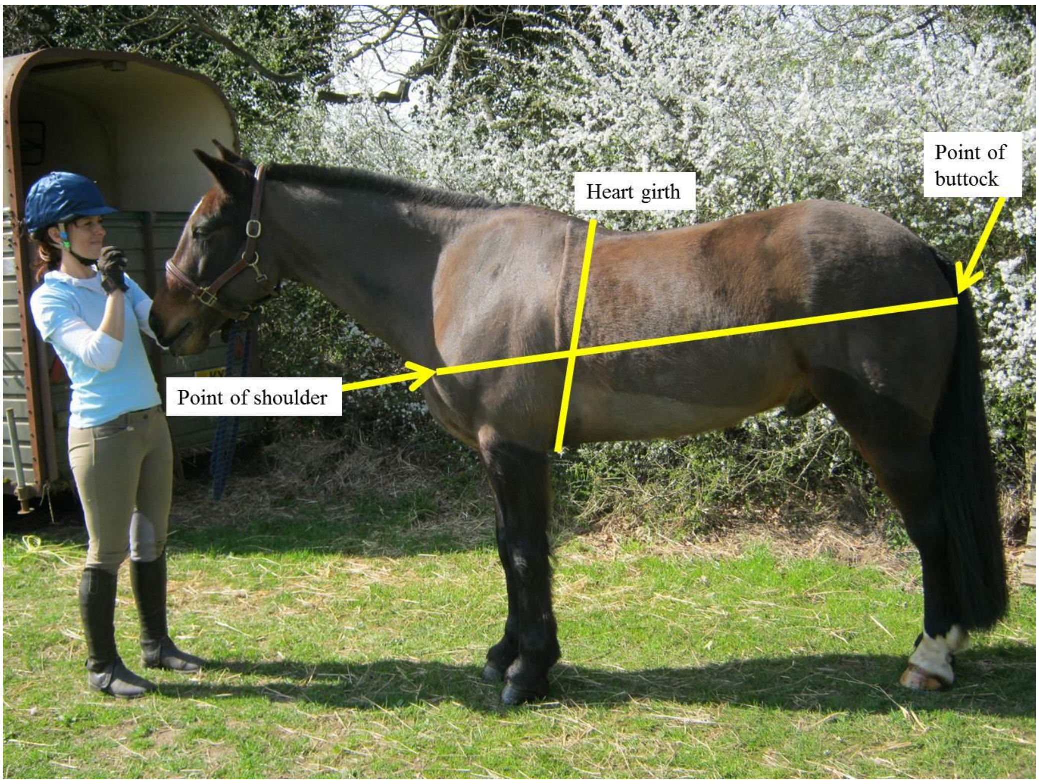

Now that you have your body length and heart girth measurements, you can enter them into your weight tracker online at www.careaboutlaminitis.org.uk, which will use a formula to calculate the estimated weight of your horse/pony! Do this on a monthly basis and watch what happens to the weight over time!

References

Wagner, E. L. & Tyler, P. J. 2011. A comparison of weight estimation methods in adult horses. *Journal of Equine Veterinary Science*, **31**: 706-710.

A special thanks to Murphy, owner Lyndsay and helper, Harriett.

How to take your horse/pony's neck measurements

Apart from doing Body Condition Scoring and Cresty Neck Scoring (which are both subjective measurements), taking neck length and thickness measurements will provide us with more objective information and will indicate the presence of any regional fat deposits on the neck. Cresty necks have been linked with metabolic disorders such as insulin resistance and are thought to be a risk factor for laminitis (Carter *et al*., 2009).

Steps 1 & 2 - Follow the steps on **pg 3**. Also review the **general instructions** and rules.

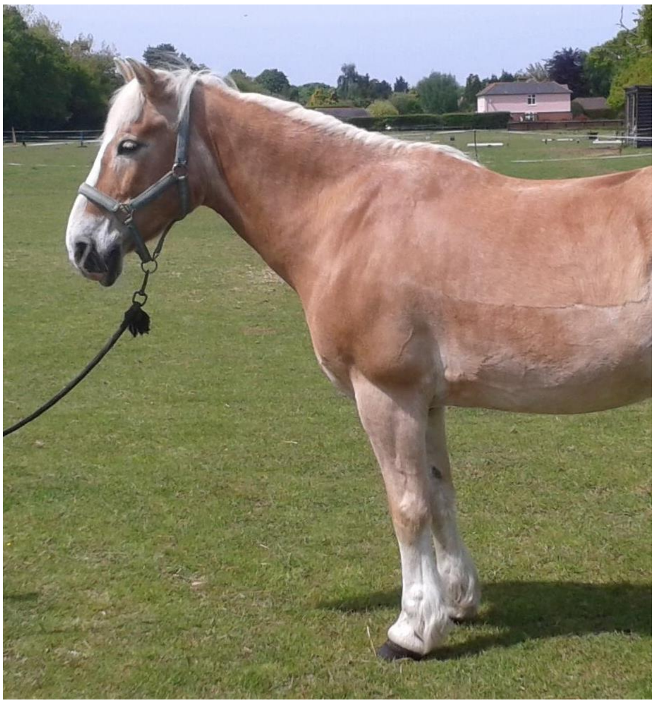

Left: *Canny models the correct neck position for taking the neck measurements. The neck should be relaxed (not tense and upright or too low and droopy!).*

Step 3 - First take the **neck length** measurement. If you have a horse/pony with an abundant mane, avoid the side on which most of the mane falls. Ask your helper to secure the tape at the poll (between the ears) and extend the tape measure to the highest point of the withers. Ensure the tape measure is flat against the neck and that it follows the actual shape of the neck (not the mane!). Record your measurement.

Step 4 - Next take the approximate halfway point of the neck length and measure the **neck thickness** at this point (referred to as the **0.50 point**). Place the tape measure all the way around the neck, parting the mane underneath, and record the measurement. This is where the regional fat deposit, or cresty neck, will be found (if present).

Step 5 - You will also need to take neck measurements at the **base of the head** and the **base of the shoulders**. These will be referred to as the **0.25** (halfway between the poll and the 0.50 neck measurement) and **0.75** (halfway between the 0.50 measurement and the highest point of the withers) points. Take both of these measurements as in Step 4 and record them.

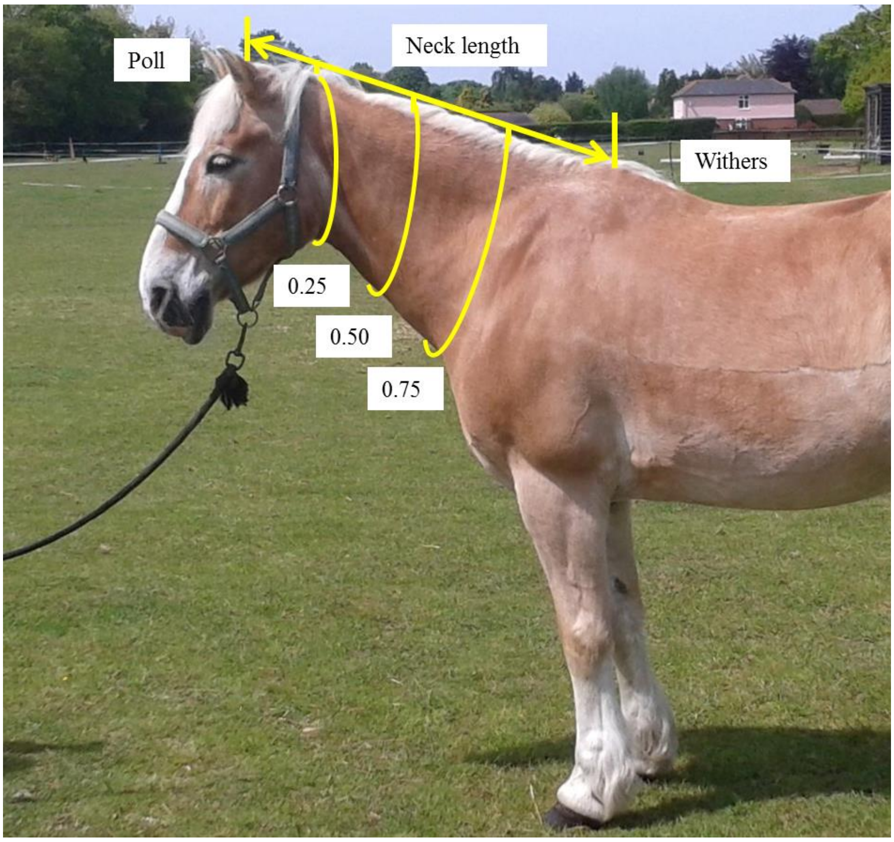

Left: *A handy diagram of where the neck measurements should be taken. Use the chart at the back of this booklet (pg 14) to keep a record.*

Just to recap!

- The **neck length** measurement (taken **from the poll** to the **highest point of the withers** – remember to measure flat against the neck and following its shape).
- The **neck thickness** measurements are taken at the **0.50** point (around halfway point of the neck length), **0.25** point (about halfway between the poll and the 0.50 point) and **0.75** point (about halfway between the 0.50 point and the highest point of the withers).

Now that you have the neck length and thickness measurements, you can enter them into your weight tracker online at www.careaboutlaminitis.org.uk. Do this on a monthly basis and watch what happens to the measurements over time!

References

Carter, R. A., Geor, R. J., Burton Staniar, W., Cubitt, T. A. and Harris, P. A. 2009. Apparent adiposity assessed by standardised scoring systems and morphometric measurements in horses and ponies. *The Veterinary Journal*, 179: 204 – 210.

Recording chart

You can input your monthly records in the chart below. Choose cm or inches as appropriate.

Horse/pony name: ....

Year & month	Condition scores					Body (cm or inches)		Neck (cm or inches)			
**2014**	BCS section:				CNS	Body length	Heart girth	Neck length	Neck 0.5	Neck 0.25	Neck 0.75
	1	2	3	Average							
**Jul**											
**Aug**											
**Sep**											
**Oct**											
**Nov**											
**Dec**											
**2015**											
**Jan**											
**Feb**											
**Mar**											
**Apr**											
**May**											
**Jun**											
**Jul**											
**Aug**											
**Sep**											
**Oct**											
**Nov**											
**Dec**											
**2016**											
**Jan**											
**Feb**											
**Mar**											
**Apr**											
**May**											
**Jun**											
**Jul**											

If you have enrolled more than 1 horse/pony, you can use the additional recording chart below. Extra recording charts can be printed off the Weight Tracker page of the website.

Horse/pony name: ....

Year & month	Condition scores					Body (cm or inches)		Neck (cm or inches)			
**2014**	BCS section:				CNS	Body length	Heart girth	Neck length	Neck 0.5	Neck 0.25	Neck 0.75
	1	2	3	Average							
**Jul**											
**Aug**											
**Sep**											
**Oct**											
**Nov**											
**Dec**											
**2015**											
**Jan**											
**Feb**											
**Mar**											
**Apr**											
**May**											
**Jun**											
**Jul**											
**Aug**											
**Sep**											
**Oct**											
**Nov**											
**Dec**											
**2016**											
**Jan**											
**Feb**											
**Mar**											
**Apr**											
**May**											
**Jun**											
**Jul**											
