# Supplementary material for: Identification of modifiable factors associated with owner-reported equine laminitis in Britain using a web-based cohort study approach
Source: BMC Vet Res. 2019 Feb 12;15:59. doi: 10.1186/s12917-019-1798-8 (PMC6373032; doi:10.1186/s12917-019-1798-8)
Supplement: Supplementary file 3 — Online owner laminitis reporting form used by participants in the cohort study of equine laminitis in Great Britain. (PDF 331 kb) [file 12917_2019_1798_MOESM3_ESM.pdf]

CARE about Laminitis

Owner Laminitis Reporting Form

Please complete the form in the event that your horse has an episode of laminitis at any time throughout the study period. If the episode is diagnosed by a vet/farrier/trimmer please complete with their consultation where possible. Thank you.

1. Name of horse/pony: ....

2. Date when clinical signs were first noted: DD/ MM/ YYYY

3. Who was this episode of laminitis confirmed by? *Please indicate all that apply.*

- ☐ My own vet, please specify date: DD/ MM/ YYYY
- ☐ Other vet/vet practice (e.g. emergency or referral vet), please specify name:
  ....
- ☐ My farrier
- ☐ My barefoot trimmer
- ☐ Me
- ☐ Other, *(please specify)* ....

4. Have you had direct experience with laminitis before? *Please indicate all that apply.*

- ☐ Yes, this horse/pony has had laminitis before
- ☐ Yes, with another horse/pony I currently own or owned
- ☐ Yes, other *(please specify)* ....
- ☐ No

Please indicate the ***presence or absence*** *of these* ***clinical signs/characteristics*** *for this episode of laminitis.*

5.

**Lameness** *(please tick one option per line)*	YES	NO	NOT ASSESSED
Recumbent (lying down)*	☐	☐	☐
Refusal to move unless forced*	☐	☐	☐
Reluctance to walk	☐	☐	☐
Lame at walk	☐	☐	☐
Lame at trot	☐	☐	☐
Short, stiff gait at walk	☐	☐	☐
Short, stiff gait at trot	☐	☐	☐
Difficulty turning	☐	☐	☐

**** If your horse or pony is showing these clinical signs please contact your vet if you have not already***

6.

**Stance** *(please tick one option per line)*	YES	NO	NOT ASSESSED
Shifting weight from leg to leg	☐	☐	☐
Leg trembling	☐	☐	☐
Front feet placed in front of body	☐	☐	☐
Hind feet placed underneath body	☐	☐	☐
Reluctance for foot to be lifted	☐	☐	☐

7.

**Feet affected** *(please tick all feet that apply)*	YES	NO	Please indicate which of these were most severely affected
Right foreleg	☐	☐	☐
Left foreleg	☐	☐	☐
Right hindleg	☐	☐	☐
Left hindleg	☐	☐	☐

8.

**Characteristics (clinical signs) of the <u>most severely</u> <u>affected foot/feet</u>** *(please tick one option per line)*		YES	NO	NOT ASSESSED
Strong/bounding pulse at the back of fetlock	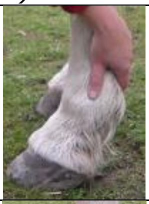	☐	☐	☐
Hoof or coronet band unusually warm to the touch	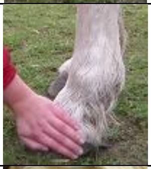	☐	☐	☐
Painful sole, especially in front of frog	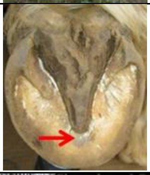	☐	☐	☐
General pain at the front of the hoof wall	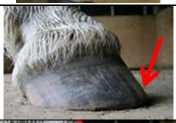	☐	☐	☐
Painful swelling at the coronary band	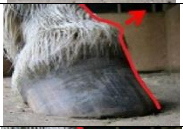	☐	☐	☐
Painful depression at the coronary band	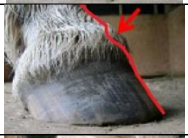	☐	☐	☐
Visible growth rings on the hoof wall (narrow at the toe and wider at the heel)	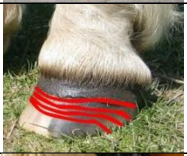	☐	☐	☐
Change in the angle of the hoof wall	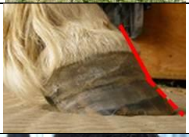	☐	☐	☐
Visible separation of the hoof wall at the coronary band* *Image courtesy of Dr L. Wells-Smith	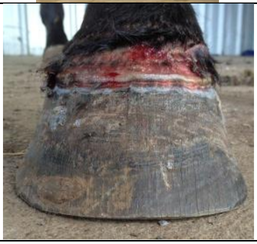	☐	☐	☐

8. **Characteristics (clinical signs) of the <u>most severely affected foot/feet</u>** *(please tick one option per line)*		YES	NO	NOT ASSESSED
Change in sole shape - becoming flat (red line) as opposed to concave/dished (blue line)	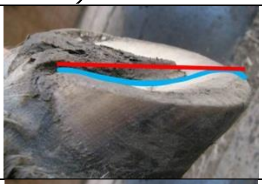	☐	☐	☐
Change in sole shape - becoming convex/bulging outwards (red line) as opposed to concave/dished (blue line) *	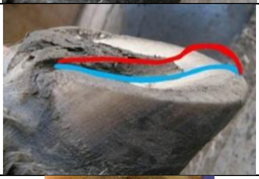	☐	☐	☐
White line abnormally stretched and wide	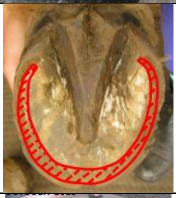	☐	☐	☐
Visible bruising in front of frog e.g. half-moon/crescent bruise*	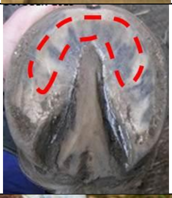	☐	☐	☐
Protrusion of the pedal bone through the bottom of the hoof (prolapsed sole)* *Image courtesy of Dr S. N. Collins	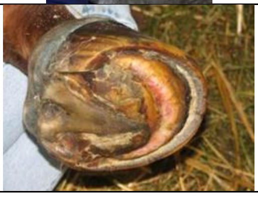	☐	☐	☐

**** If your horse or pony is showing these clinical signs please contact your vet if you have not already***

9. What techniques were used to confirm the most recent episode of laminitis in your horse?

Please indicate all that apply.

☐ Clinical signs

☐ X-ray of hooves

☐ Other *(please specify)* ....

CARE about Laminitis

10. Have any of these additional factors helped confirm laminitis in your horse?

Please indicate one option per line.

	YES	NO	DON'T KNOW
Breed type	☐	☐	☐
Age	☐	☐	☐
Body condition score (overweight)	☐	☐	☐
Body condition score (underweight)	☐	☐	☐
Previous history of laminitis	☐	☐	☐
Quality of grazing or pasture available	☐	☐	☐
Accidental carbohydrate/concentrates overload	☐	☐	☐
Season/Weather conditions	☐	☐	☐
Specify any additional factors that helped confirm laminitis in our horse?			

11. Any other comments relevant to this laminitis episode?

....

....

RVC Royal Veterinary College University of London

WorldHorseWelfare

AHT Animal Health Trust
