# Supplementary material for: Identification of modifiable factors associated with owner-reported equine laminitis in Britain using a web-based cohort study approach
Source: BMC Vet Res. 2019 Feb 12;15:59. doi: 10.1186/s12917-019-1798-8 (PMC6373032; doi:10.1186/s12917-019-1798-8)
Supplement: Supplementary file 4 — A table showing the demographics of the cohort population of horses and ponies at baseline presented in descending order of frequency, unless the variable was ordinal in nature. (DOCX 23 kb) [file 12917_2019_1798_MOESM4_ESM.docx]

Table 3

Demographics of the cohort population at baseline presented in descending order of frequency, unless the variable was ordinal in nature.

| Descriptive variable | Frequency | Percentage | 95% CI limit |
| --- | --- | --- | --- |
| *Country (n=1,070)* |  |  |  |
| England | 899 | 84.0 | 81.8, 86.2 |
| Scotland | 101 | 9.4 | 7.7, 11.2 |
| Wales | 70 | 6.5 | 5.1, 8.0 |
|  |  |  |  |
| *Ownership (n=1,070)* |  |  |  |
| Privately owned | 912 | 85.2 | 83.1, 87.4 |
| Loan/care agreement | 158 | 14.8 | 12.6, 16.9 |
|  |  |  |  |
| *Sex (n=1,070)* |  |  |  |
| Gelding | 615 | 57.5 | 54.5, 60.4 |
| Mare/filly | 448 | 41.9 | 38.9, 41.9 |
| Stallion/colt | 7 | 0.7 | 0.2, 1.1 |
|  |  |  |  |
| *Breed (n=1,070)* |  |  |  |
| Pure breed | 529 | 49.4 | 46.4, 52.4 |
| Cross-breed | 494 | 46.2 | 43.2, 49.2 |
| Don’t know | 47 | 4.4 | 3.2, 5.6 |
|  |  |  |  |
| *Breed types and their crosses (n=1,070)* |  |  |  |
| Native pony | 436 | 40.7 | 37.8, 43.7 |
| Welsh | 175 | 40.1 | 35.5, 44.7 |
| Shetland | 60 | 13.8 | 10.5, 17.0 |
| New Forest | 57 | 13.1 | 9.9, 16.2 |
| Connemara | 47 | 10.8 | 7.9, 13.7 |
| Other (including Dartmoor, Exmoor, Dales, Fell, Highland) | 97 | 22.2 | 18.3, 26.2 |
| Thoroughbred | 111 | 10.4 | 8.5, 12.2 |
| Other horse breed^1^ | 96 | 9.0 | 7.3, 10.7 |
| Other pony breed^2^ | 93 | 8.7 | 7.0, 10.4 |
| Cob | 88 | 8.2 | 6.6, 9.9 |
| Welsh horse | 66 | 6.2 | 4.7, 7.6 |
| Draught^3^ | 64 | 6.0 | 4.6, 7.4 |
| Warmblood | 60 | 5.6 | 4.2, 7.0 |
| Arabian | 56 | 5.2 | 3.9, 6.6 |
|  |  |  |  |
| *Body condition score (n=1,068)* |  |  |  |
| 0 | 0 | 0.0 | - |
| 1 | 3 | 0.3 | 0.0, 0.6 |
| 2 | 76 | 7.1 | 5.6, 8.7 |
| 3 | 630 | 59.0 | 56.0, 61.9 |
| 4 | 340 | 31.8 | 29.0, 34.6 |
| 5 | 19 | 1.8 | 1.0, 2.6 |
|  |  |  |  |
| *Cresty neck score (n=1,057)* |  |  |  |
| 0 | 25 | 2.4 | 1.4, 3.3 |
| 1 | 221 | 20.9 | 18.5, 23.4 |
| 2 | 523 | 49.5 | 46.5, 52.5 |
| 3 | 253 | 23.9 | 21.4, 26.5 |
| 4 | 32 | 3.0 | 2.0, 4.1 |
| 5 | 3 | 0.3 | 0.0, 0.6 |
|  |  |  |  |
| *Weight estimation method (n=1,053)* |  |  |  |
| Study weight tracker | 482 | 45.8 | 42.8, 48.8 |
| Commercial weight tape | 315 | 29.9 | 27.1, 32.7 |
| Estimated by eye | 140 | 13.3 | 11.2, 15.3 |
| Weighbridge | 116 | 11.0 | 9.1, 12.9 |
|  |  |  |  |

^1^-Includes other horse breeds not already represented, ^2^-Includes other pony breeds not already represented (other horse and pony breeds were split by height where a breed was not specified), ^3^-Includes Irish Draughts and heavy horse breeds.
