# Supplementary material for: Identification of modifiable factors associated with owner-reported equine laminitis in Britain using a web-based cohort study approach
Source: BMC Vet Res. 2019 Feb 12;15:59. doi: 10.1186/s12917-019-1798-8 (PMC6373032; doi:10.1186/s12917-019-1798-8)
Supplement: Supplementary file 5 — A table showing the univariable Cox regression results of variables associated with laminitis (P < 0.25) in a cohort study of horses and ponies in Great Britain. (DOCX 108 kb) [file 12917_2019_1798_MOESM5_ESM.docx]

**Table 4 :** Univariable Cox regression results of variables associated with laminitis (P<0.25) in a cohort study of horses and ponies in Great Britain.

| **Variable** | **Laminitis episodes** | **HYAR/ 100** | **Hazard ratio** | **95% CI** | **Wald P-value** | **LRS^#^ P-value** |
| --- | --- | --- | --- | --- | --- | --- |
| **Animal-level factors** |  |  |  |  |  |  |
| *Type of ownership* |  |  |  |  |  | *0.009* |
| Own | 101 | 9.1 | Ref. |  |  |  |
| Full loan | 20 | 1.0 | 1.8 | 1.1, 2.9 | 0.02 |  |
| Other type of ownership | 2 | 0.6 | 0.3 | 0.1, 1.3 | 0.12 |  |
|  |  |  |  |  |  |  |
| *Duration of ownership (quartiles)* | |  |  |  |  | *0.02* |
| <=3.6yrs | 18 | 2.6 | Ref. |  |  |  |
| >3.6-8.0yrs | 33 | 2.6 | 1.8 | 1.0, 3.2 | 0.04 |  |
| >8.0-12.9yrs | 40 | 2.4 | 2.4 | 1.4, 4.2 | 0.002 |  |
| >12.9yrs | 32 | 2.7 | 1.7 | 1.0, 3.1 | 0.06 |  |
|  |  |  |  |  |  |  |
| *Duration of ownership* | |  |  |  |  | *0.01* |
| <=1yr | 3 | 0.7 | 0.5 | 0.2, 1.8 | 0.30 |  |
| >1-5yrs | 21 | 2.7 | Ref. |  |  |  |
| >5-10yrs | 45 | 3.0 | 1.9 | 1.2, 3.3 | 0.01 |  |
| >10-15yrs | 31 | 2.1 | 1.8 | 1.1, 3.2 | 0.03 |  |
| >15yrs | 23 | 1.8 | 1.6 | 0.9, 2.9 | 0.11 |  |
|  |  |  |  |  |  |  |
| *Number of horses enrolled* | |  |  |  |  | *0.004* |
| 1 horse | 62 | 4.8 | Ref. |  |  |  |
| 2 horses | 41 | 2.8 | 1.1 | 0.7, 1.6 | 0.63 |  |
| >2 horses | 20 | 3.1 | 0.5 | 0.3, 0.8 | 0.006 |  |
|  |  |  |  |  |  |  |
| *Country of residence* |  |  |  |  |  | *0.12* |
| England | 104 | 9.1 | Ref. |  |  |  |
| Scotland | 8 | 1.0 | 0.7 | 0.3, 1.4 | 0.28 |  |
| Wales | 11 | 0.6 | 1.7 | 0.9, 3.3 | 0.08 |  |
|  |  |  |  |  |  |  |
| *Sex category* |  |  |  |  |  | *0.15* |
| Male | 64 | 6.2 | Ref. |  |  |  |
| Female | 59 | 4.4 | 1.3 | 0.9, 1.8 | 0.15 |  |
|  |  |  |  |  |  |  |
| *Breed type 1 (including breed crosses)* | |  |  |  |  | *<0.001* |
| Arab | 4 | 0.5 | 1.0 | 0.3, 3.1 | 0.98 |  |
| Cob | 4 | 1.0 | 0.5 | 0.1, 1.5 | 0.20 |  |
| Connemara | 14 | 0.5 | 3.6 | 1.6, 8.0 | 0.002 |  |
| New Forest | 13 | 0.5 | 3.1 | 1.4, 7.2 | 0.006 |  |
| Shetland | 12 | 0.6 | 2.4 | 1.0, 5.4 | 0.05 |  |
| Welsh pony | 24 | 1.6 | 1.8 | 0.8, 3.7 | 0.14 |  |
| Other native pony breed | 16 | 1.2 | 1.6 | 0.7, 3.6 | 0.23 |  |
| Other horse | 5 | 0.9 | 0.7 | 0.2, 2.0 | 0.48 |  |
| Other pony | 8 | 0.9 | 1.1 | 0.4, 2.8 | 0.81 |  |
| Draught | 3 | 0.6 | 0.6 | 0.2, 2.1 | 0.40 |  |
| Thoroughbred | 10 | 1.2 | Ref. |  |  |  |
| Warmblood | 5 | 0.5 | 1.2 | 0.4, 3.5 | 0.76 |  |
| Welsh horse | 5 | 0.7 | 0.8 | 0.3, 2.3 | 0.65 |  |
|  |  |  |  |  |  |  |
| *Breed type 2 (including breed crosses)* | |  |  |  |  | *<0.001* |
| Arab | 4 | 0.5 | 1.0 | 0.3, 3.1 | 0.98 |  |
| Cob | 4 | 1.0 | 0.5 | 0.1, 1.5 | 0.20 |  |
| Native pony | 79 | 4.3 | 2.2 | 1.1, 4.2 | 0.02 |  |
| Welsh horse | 5 | 0.7 | 0.8 | 0.3, 2.3 | 0.65 |  |
| Other horse | 5 | 0.9 | 0.7 | 0.2, 2.0 | 0.48 |  |
| Other pony | 8 | 0.9 | 1.1 | 0.4, 2.8 | 0.82 |  |
| Draught | 3 | 0.6 | 0.6 | 0.2, 2.1 | 0.40 |  |
| Thoroughbred | 10 | 1.2 | Ref. |  |  |  |
| Warmblood | 5 | 0.5 | 1.2 | 0.4, 3.5 | 0.76 |  |
|  |  |  |  |  |  |  |
| *Breed category 1 (including breed crosses)* | |  |  |  |  | *<0.001* |
| Native pony | 79 | 4.3 | 2.6 | 1.8, 3.8 | <0.001 |  |
| Other breed | 44 | 6.3 | Ref. |  |  |  |
|  |  |  |  |  |  |  |
| *Age category (quartiles)* |  |  |  |  |  | *0.03* |
| <=11yrs | 25 | 3.1 | Ref. |  |  |  |
| 12-16yrs | 29 | 2.5 | 1.5 | 0.9, 2.5 | 0.16 |  |
| 17-20yrs | 40 | 2.4 | 2.1 | 1.3, 3.5 | 0.003 |  |
| >=21yrs | 29 | 2.7 | 1.3 | 0.8, 2.3 | 0.30 |  |
|  |  |  |  |  |  |  |
| *Age category* |  |  |  |  |  | *0.05* |
| <5yrs | 3 | 0.4 | Ref. |  |  |  |
| 5-10yrs | 19 | 2.3 | 1.2 | 0.3, 4.0 | 0.80 |  |
| 11-15yrs | 24 | 2.3 | 1.5 | 0.4, 4.9 | 0.52 |  |
| 16-20yrs | 48 | 2.9 | 2.4 | 0.7, 7.7 | 0.14 |  |
| 21-25yrs | 23 | 1.9 | 1.7 | 0.5, 5.7 | 0.38 |  |
| >25yrs | 6 | 0.8 | 1.1 | 0.3, 4.3 | 0.94 |  |
|  |  |  |  |  |  |  |
| *Height* |  |  |  |  |  | *0.002* |
| Height in cm (continuous) |  |  | 0.99 | 0.98, 0.995 | 0.001 |  |
|  |  |  |  |  |  |  |
| *Height (binary)* |  |  |  |  |  | *0.001* |
| <=147.32cm (pony) | 79 | 5.3 | 1.8 | 1.3, 2.6 | 0.001 |  |
| >147.32cm (horse) | 44 | 5.4 | Ref. |  |  |  |
|  |  |  |  |  |  |  |
| *Height category 1 (quartiles)* | |  |  |  |  | *0.004* |
| <=137.2cm | 46 | 3.0 | 2.6 | 1.4, 4.9 | 0.002 |  |
| >137.2-147.3cm | 33 | 2.3 | 2.5 | 1.3, 4.7 | 0.006 |  |
| >147.3-157.5cm | 31 | 3.1 | 1.7 | 0.9, 3.3 | 0.11 |  |
| >157.5cm | 13 | 2.3 | Ref. |  |  |  |
|  |  |  |  |  |  |  |
| *Height category 2* |  |  |  |  |  | *0.08* |
| <=102cm | 10 | 0.6 | 2.9 | 1.1, 7.3 | 0.03 |  |
| >102-122cm | 15 | 1.0 | 2.4 | 1.0, 5.6 | 0.05 |  |
| >122-142cm | 24 | 1.6 | 2.4 | 1.1, 5.4 | 0.03 |  |
| >142-163cm | 66 | 6.2 | 1.7 | 0.8, 3.5 | 0.16 |  |
| >163cm | 8 | 1.3 | Ref. |  |  |  |
|  |  |  |  |  |  |  |
| *Coat colour* |  |  |  |  |  | *0.11* |
| Bay | 36 | 3.2 | Ref. |  |  |  |
| Black | 18 | 1.1 | 1.5 | 0.8, 2.6 | 0.18 |  |
| Chestnut | 19 | 2.0 | 0.8 | 0.5, 1.4 | 0.50 |  |
| Coloured | 10 | 1.4 | 0.6 | 0.3, 1.3 | 0.19 |  |
| Grey | 27 | 1.7 | 1.4 | 0.9, 2.4 | 0.15 |  |
| Other | 13 | 1.3 | 0.9 | 0.5, 1.7 | 0.78 |  |
|  |  |  |  |  |  |  |
| *Body weight* |  |  |  |  |  | *0.005* |
| Estimated weight in kg (continuous) |  |  | 0.998 | 0.997, 0.999 | 0.005 |  |
|  |  |  |  |  |  |  |
| *Body weight category 1 (quartiles)* | |  |  |  |  | *0.004* |
| <=370kg | 45 | 2.8 | 2.3 | 1.3, 3.9 | 0.003 |  |
| >370-464kg | 35 | 2.5 | 2.0 | 1.1, 3.6 | 0.02 |  |
| >464-530kg | 18 | 2.5 | Ref. |  |  |  |
| >530kg | 23 | 2.7 | 1.2 | 0.7, 2.2 | 0.54 |  |
|  |  |  |  |  |  |  |
| *Weight change since last submission* | |  |  |  |  | *0.09* |
| Lost | 25 | 2.6 | 0.98 | 0.6, 1.6 | 0.94 |  |
| Maintained | 60 | 5.9 | Ref. |  |  |  |
| Gained | 35 | 2.1 | 1.7 | 1.1, 2.6 | 0.03 |  |
| Not specified | 3 | 0.1 | 2.0 | 0.6, 6.4 | 0.24 |  |
|  |  |  |  |  |  |  |
| *Weight change since last submission (re-categorised)* |  |  |  |  |  | *0.04* |
| Maintained/lost | 85 | 8.4 | Ref. |  |  |  |
| Gained | 35 | 2.1 | 1.7 | 1.1, 2.5 | 0.02 |  |
| Not specified | 3 | 0.1 | 2.0 | 0.6, 6.4 | 0.23 |  |
|  |  |  |  |  |  |  |
| *Cresty neck score (CNS) category* | |  |  |  |  | *0.20* |
| Slight to no crest  (CNS 0-2) | 82 | 7.7 | Ref. |  |  |  |
| Notable crest (CNS 3-5) | 40 | 3.0 | 1.3 | 0.9, 1.9 | 0.20 |  |
|  |  |  |  |  |  |  |
| **Turnout and management of grazing** | |  |  |  |  |  |
| Turnout routine |  |  |  |  |  |  |
| *Turnout* |  |  |  |  |  | *<0.001* |
| Partly outdoors/partly stabled | 82 | 5.5 | Ref. |  |  |  |
| Entirely outdoors | 35 | 4.9 | 0.5 | 0.3, 0.7 | <0.001 |  |
| Entirely stabled | 6 | 0.3 | 1.4 | 0.6, 3.2 | 0.43 |  |
|  |  |  |  |  |  |  |
| *Turnout herd size* |  |  |  |  |  | *0.10* |
| Individual/no turnout | 34 | 2.5 | Ref. |  |  |  |
| 1 other horse | 43 | 3.9 | 0.8 | 0.5, 1.3 | 0.38 |  |
| 2-5 other horses | 42 | 3.3 | 0.9 | 0.6, 1.5 | 0.76 |  |
| >5 other horses | 4 | 0.9 | 0.3 | 0.1, 0.9 | 0.04 |  |
|  |  |  |  |  |  |  |
| Type of turnout horse has access to |  |  |  |  |  |  |
| *Access to grass turnout* |  |  |  |  |  | *0.10* |
| Yes | 101 | 9.5 | 0.6 | 0.4, 1.1 | 0.09 |  |
| No | 18 | 1.1 | Ref. |  |  |  |
|  |  |  |  |  |  |  |
| *Turnout type most time spent on* |  |  |  |  |  | *0.05* |
| Grass | 90 | 8.9 | Ref. |  |  |  |
| Bare/dirt | 8 | 0.6 | 1.3 | 0.6, 2.8 | 0.44 |  |
| Soft surface | 10 | 0.4 | 2.4 | 1.2, 4.6 | 0.009 |  |
| Hard surface | 3 | 0.2 | 1.3 | 0.4, 4.2 | 0.63 |  |
| Other/no turnout | 12 | 0.6 | 2.0 | 1.1, 3.7 | 0.02 |  |
|  |  |  |  |  |  |  |
| *Turnout type most time spent on (binary)* | |  |  |  |  | *0.006* |
| Grass | 90 | 8.9 | 0.6 | 0.4, 0.8 | 0.004 |  |
| Non-grass/no turnout | 33 | 1.8 | Ref. |  |  |  |
|  |  |  |  |  |  |  |
| Type of land bordering turnout area |  |  |  |  |  |  |
| *Grazing pasture* |  |  |  |  |  | *0.20* |
| Yes | 90 | 8.6 | 0.8 | 0.5, 1.2 | 0.19 |  |
| No/not turned out | 29 | 2.1 | Ref. |  |  |  |
|  |  |  |  |  |  |  |
| *Organic livestock farm* |  |  |  |  |  | *0.16* |
| Yes | 8 | 0.4 | 1.7 | 0.8, 3.6 | 0.13 |  |
| No/not turned out | 111 | 10.2 | Ref. |  |  |  |
|  |  |  |  |  |  |  |
| *Agricultural crop* |  |  |  |  |  | *0.01* |
| Yes | 31 | 3.9 | 0.6 | 0.4, 0.9 | 0.02 |  |
| No/not turned out | 88 | 6.7 | Ref. |  |  |  |
|  |  |  |  |  |  |  |
| *Woodland* |  |  |  |  |  | *0.02* |
| Yes | 21 | 2.8 | 0.6 | 0.4, 0.9 | 0.03 |  |
| No/not turned out | 98 | 7.8 | Ref. |  |  |  |
|  |  |  |  |  |  |  |
| Recent reintroduction to grass |  |  |  |  |  |  |
| *Reintroduction to grass within past month* | |  |  |  |  | *<0.001* |
| Yes | 18 | 0.5 | 3.4 | 2.1, 5.6 | <0.001 |  |
| No/no grass access | 105 | 10.2 | Ref. |  |  |  |
|  |  |  |  |  |  |  |
| *Time access to grass was unavailable* |  |  |  |  |  | *<0.001* |
| Up to 3 weeks | 6 | 0.1 | 5.2 | 2.3, 12.0 | <0.001 |  |
| More than 3 weeks | 9 | 0.3 | 2.6 | 1.3, 5.2 | 0.006 |  |
| Grass always/never available | 105 | 10.2 | Ref. |  |  |  |
|  |  |  |  |  |  |  |
| *Initial hours of grass reintroduction* |  |  |  |  |  | *<0.001* |
| Up to 1 hour | 12 | 0.2 | 5.3 | 2.9, 9.6 | <0.001 |  |
| More than 1 hour | 6 | 0.3 | 2.1 | 0.9, 4.9 | 0.07 |  |
| Grass always/never available | 102 | 10.2 | Ref. |  |  |  |
|  |  |  |  |  |  |  |
| Access to grass |  |  |  |  |  |  |
| *Average time spent on grass* |  |  |  |  |  | *0.002* |
| 0 hours | 11 | 0.8 | 1.0 | 0.5, 1.9 | 0.97 |  |
| Up to 1 hour | 6 | 0.2 | 2.2 | 0.9, 5.1 | 0.08 |  |
| >1-3 hours | 9 | 0.4 | 1.7 | 0.8, 3.4 | 0.16 |  |
| >3-6 hours | 12 | 1.0 | 0.8 | 0.4, 1.4 | 0.4 |  |
| >6-12 hours | 43 | 2.9 | Ref. |  |  |  |
| >12-23 hours | 18 | 1.8 | 0.7 | 0.4, 1.1 | 0.14 |  |
| 24 hours | 24 | 3.6 | 0.4 | 0.3, 0.7 | 0.001 |  |
|  |  |  |  |  |  |  |
| *Average time spent on grass (re-categorised)* | |  |  |  |  | *0.003* |
| 0 hours | 11 | 0.8 | 1.9 | 1.0, 3.7 | 0.06 |  |
| >0-3 hours | 15 | 0.5 | 3.6 | 2.0, 6.4 | <0.001 |  |
| >3-12 hours | 55 | 3.9 | 1.8 | 1.2, 2.7 | 0.004 |  |
| >12 hours | 42 | 5.5 | Ref. |  |  |  |
|  |  |  |  |  |  |  |
| *Time of day access to grass available* |  |  |  |  |  | *0.003* |
| Morning only | 11 | 0.4 | 1.8 | 0.9, 3.5 | 0.08 |  |
| Afternoon only | 2 | 0.2 | 0.8 | 0.2, 3.5 | 0.81 |  |
| Daylight hours only | 51 | 3.5 | Ref. |  |  |  |
| Night-time hours only | 12 | 0.8 | 1.0 | 0.6, 2.0 | 0.90 |  |
| Day and night | 36 | 5.0 | 0.5 | 0.3, 0.8 | 0.002 |  |
| Grass not available | 11 | 0.8 | 1.0 | 0.5, 2.0 | 0.93 |  |
|  |  |  |  |  |  |  |
| *Time of day access to grass available (re-categorised)* | | |  |  |  | *<0.001* |
| Morning only | 11 | 0.4 | 3.6 | 1.8, 7.0 | <0.001 |  |
| Day and night | 36 | 5.0 | Ref. |  |  |  |
| Other/grass not available | 76 | 5.3 | 2.0 | 1.3, 3.0 | 0.001 |  |
|  |  |  |  |  |  |  |
| Grass restriction during turnout |  |  |  |  |  |  |
| *Access to grass restricted* |  |  |  |  |  | *<0.001* |
| Yes | 77 | 5.0 | 1.9 | 1.3, 2.7 | 0.001 |  |
| No/grass not available | 46 | 5.6 | Ref. |  |  |  |
|  |  |  |  |  |  |  |
| *Grazing muzzle used* |  |  |  |  |  | *<0.001* |
| Yes | 22 | 0.8 | 3.0 | 1.9, 4.7 | <0.001 |  |
| No/grass not available | 97 | 9.9 | Ref. |  |  |  |
|  |  |  |  |  |  |  |
| *Wearing of grazing muzzle* |  |  |  |  |  | *<0.001* |
| Part of the time while grazing | 12 | 0.3 | 3.5 | 1.9, 6.4 | <0.001 |  |
| All of the time while grazing /not worn | 107 | 10.3 | Ref. |  |  |  |
|  |  |  |  |  |  |  |
| *Turnout restricted to restrict grass intake* |  |  |  |  |  | *<0.001* |
| Yes | 36 | 1.6 | 2.4 | 1.6, 3.6 | <0.001 |  |
| No/grass not available | 83 | 9.0 | Ref. |  |  |  |
|  |  |  |  |  |  |  |
| Grazing type and management |  |  |  |  |  |  |
| *Type of grazing* |  |  |  |  |  | *0.18* |
| Meadow pasture | 34 | 3.9 | 0.7 | 0.4, 1.0 | 0.05 |  |
| Mature grass paddock (seeded >5years ago) | 70 | 5.4 | Ref. |  |  |  |
| New grass paddock (seeded within 5 years) | 2 | 0.2 | 0.7 | 0.2, 2.7 | 0.57 |  |
| Other/don’t know | 15 | 1.1 | 1.1 | 0.6, 1.9 | 0.71 |  |
|  |  |  |  |  |  |  |
| *Grass length* |  |  |  |  |  | *0.12* |
| <5cm | 95 | 7.6 | Ref. |  |  |  |
| 5-15cm | 23 | 2.5 | 0.7 | 0.5, 1.1 | 0.16 |  |
| >15-30cm | 2 | 0.4 | 0.4 | 0.1, 1.5 | 0.17 |  |
| >30cm | 3 | 0.1 | 2.0 | 0.6, 6.4 | 0.23 |  |
|  |  |  |  |  |  |  |
| *Pasture fertilisation* |  |  |  |  |  | *0.18* |
| Yes | 43 | 4.1 | 1.0 | 0.7, 1.5 | 0.98 |  |
| No/grass not available | 50 | 4.7 | Ref. |  |  |  |
| Don’t know | 30 | 1.9 | 1.5 | 0.9, 2.3 | 0.08 |  |
|  |  |  |  |  |  |  |
| *Time of fertilisation* |  |  |  |  |  | *0.03* |
| Never | 50 | 4.7 | Ref. |  |  |  |
| <1 month ago | 2 | 0.1 | 1.4 | 0.3, 5.7 | 0.65 |  |
| >1-4 months ago | 1 | 0.6 | 0.2 | 0.02, 1.2 | 0.08 |  |
| >4 months ago | 40 | 3.4 | 1.1 | 0.7, 1.7 | 0.60 |  |
| Don’t know | 30 | 1.9 | 1.5 | 0.9, 2.3 | 0.08 |  |
|  |  |  |  |  |  |  |
| *Domestic animals sharing pasture* |  |  |  |  |  | *0.15* |
| Yes | 25 | 1.7 | 1.4 | 0.9, 2.2 | 0.14 |  |
| No | 98 | 9.0 |  |  |  |  |
|  |  |  |  |  |  |  |
| **Stabling and indoor environment** |  |  |  |  |  |  |
| Stabling routine |  |  |  |  |  |  |
| *Stabled for any part of the day* |  |  |  |  |  | *<0.001* |
| Yes | 86 | 5.8 | 1.9 | 1.3, 2.9 | 0.001 |  |
| No | 37 | 4.9 | Ref. |  |  |  |
|  |  |  |  |  |  |  |
| *Average time spent stabled* |  |  |  |  |  | *<0.001* |
| 0-3 hours | 37 | 5.3 | Ref. |  |  |  |
| >3-12 hours | 53 | 2.6 | 2.9 | 1.9, 4.5 | <0.001 |  |
| >12-23 hours | 19 | 2.3 | 1.2 | 0.7, 2.0 | 0.57 |  |
| >23 hours | 5 | 0.2 | 3.8 | 1.5, 9.6 | 0.005 |  |
| Free access | 6 | 0.3 | 2.7 | 1.1, 6.3 | 0.03 |  |
|  |  |  |  |  |  |  |
| *Average time spent stabled (re-categorised)* | |  |  |  |  | *<0.001* |
| Not stabled/free access | 40 | 5.2 | 0.8 | 0.5, 1.3 | 0.40 |  |
| Up to 12 hours | 56 | 3.0 | 1.9 | 1.2, 3.1 | 0.007 |  |
| More than 12 hours | 24 | 2.4 | Ref. |  |  |  |
|  |  |  |  |  |  |  |
| *Time of day stabled* |  |  |  |  |  | *0.005* |
| Day | 16 | 1.2 | 1.0 | 0.5, 1.7 | 0.88 |  |
| Night | 53 | 3.8 | Ref. |  |  |  |
| Day and night (24 hours) | 11 | 0.5 | 1.6 | 0.8, 3.0 | 0.18 |  |
| Other/not stabled | 40 | 5.2 | 0.6 | 0.4, 0.8 | 0.005 |  |
|  |  |  |  |  |  |  |
| Reason for stabling |  |  |  |  |  |  |
| *Adverse weather* |  |  |  |  |  | *0.20* |
| Yes | 30 | 2.2 | 1.3 | 0.9, 2.0 | 0.19 |  |
| No/not stabled | 88 | 8.5 | Ref. |  |  |  |
|  |  |  |  |  |  |  |
| *To reduce grass intake* |  |  |  |  |  | *<0.001* |
| Yes | 63 | 3.1 | 2.8 | 2.0, 4.0 | <0.001 |  |
| No/not stabled | 55 | 7.6 | Ref. |  |  |  |
|  |  |  |  |  |  |  |
| *To prevent illness/injury* |  |  |  |  |  | *0.16* |
| Yes | 5 | 0.2 | 2.0 | 0.8, 5.0 | 0.12 |  |
| No/not stabled | 113 | 10.4 | Ref. |  |  |  |
|  |  |  |  |  |  |  |
| *Owner’s preference* |  |  |  |  |  | *0.07* |
| Yes | 27 | 1.7 | 1.5 | 1.0, 2.3 | 0.06 |  |
| No/not stabled | 91 | 8.9 | Ref. |  |  |  |
|  |  |  |  |  |  |  |
| *Yard rules* |  |  |  |  |  | *0.04* |
| Yes | 4 | 0.8 | 0.4 | 0.2, 1.1 | 0.08 |  |
| No/not stabled | 114 | 9.8 | Ref. |  |  |  |
|  |  |  |  |  |  |  |
| Stabling environment |  |  |  |  |  |  |
| *Stable size categories* |  |  |  |  |  | *0.01* |
| <140 ft^2^ | 26 | 1.4 | 2.0 | 1.3, 3.3 | 0.003 |  |
| More than 140 to 144 ft^2^ | 34 | 2.3 | 1.6 | 1.1, 2.5 | 0.02 |  |
| More than 144 to 172 ft^2^ | 5 | 0.6 | 0.9 | 0.4, 2.3 | 0.85 |  |
| More than 172 ft^2^/not stabled | 57 | 6.2 | Ref. |  |  |  |
|  |  |  |  |  |  |  |
| *Type of stable* |  |  |  |  |  | *0.005* |
| Not stabled/free access | 38 | 5.0 | Ref. |  |  |  |
| Individual stable not within a barn | 56 | 3.5 | 2.1 | 1.4, 3.1 | 0.001 |  |
| Individual stable within a barn | 24 | 1.9 | 1.7 | 1.0, 2.8 | 0.05 |  |
| Communal space/barn | 2 | 0.3 | 0.9 | 0.2, 3.6 | 0.86 |  |
|  |  |  |  |  |  |  |
| *Stable base surface* |  |  |  |  |  | *<0.001* |
| Hard | 75 | 5.3 | 2.0 | 1.3, 3.0 | 0.001 |  |
| Soft | 11 | 0.5 | 3.4 | 1.7, 6.7 | <0.001 |  |
| Not stabled | 34 | 4.9 | Ref. |  |  |  |
|  |  |  |  |  |  |  |
| *Rubber matting used* |  |  |  |  |  | *0.05* |
| Yes | 58 | 4.2 | 1.4 | 1.0, 2.0 | 0.05 |  |
| No/not stabled | 62 | 6.4 | Ref. |  |  |  |
|  |  |  |  |  |  |  |
| *Bedding used in stable* |  |  |  |  |  | *<0.001* |
| Yes | 86 | 5.6 | 2.2 | 1.5, 3.3 | <0.001 |  |
| No/not stabled | 34 | 5.0 | Ref. |  |  |  |
|  |  |  |  |  |  |  |
| Type of bedding used |  |  |  |  |  |  |
| *Shavings* |  |  |  |  |  | *0.01* |
| Yes | 43 | 2.7 | 1.6 | 1.1, 2.4 | 0.009 |  |
| No/not stabled | 75 | 7.9 | Ref. |  |  |  |
|  |  |  |  |  |  |  |
| *Paper* |  |  |  |  |  | *0.08* |
| Yes | 3 | 0.1 | 3.5 | 1.1, 10.9 | 0.03 |  |
| No/not stabled | 115 | 10.5 | Ref. |  |  |  |
|  |  |  |  |  |  |  |
| **Supplementary feeding** |  |  |  |  |  |  |
| *Any feed given (including forage and concentrates)* | |  |  |  |  | *0.21* |
| Yes | 118 | 9.9 | 1.7 | 0.7, 4.2 | 0.25 |  |
| No | 5 | 0.7 | Ref. |  |  |  |
|  |  |  |  |  |  |  |
| Supplementary forage |  |  |  |  |  |  |
| *Supplementary forage fed* |  |  |  |  |  | *0.01* |
| Yes | 112 | 8.8 | 2.1 | 1.1, 3.9 | 0.02 |  |
| No | 11 | 1.9 | Ref. |  |  |  |
|  |  |  |  |  |  |  |
| Type of forage fed |  |  |  |  |  |  |
| *Grass hay* |  |  |  |  |  | *0.02* |
| Yes | 93 | 7.3 | 1.7 | 1.1, 2.6 | 0.02 |  |
| No | 25 | 3.4 | Ref. |  |  |  |
|  |  |  |  |  |  |  |
| *Straw* |  |  |  |  |  | *0.15* |
| Yes | 7 | 0.4 | 1.8 | 0.9, 4.0 | 0.12 |  |
| No | 111 | 10.3 | Ref. |  |  |  |
|  |  |  |  |  |  |  |
| *Ryegrass forage fed (hay and haylage combined)* |  |  |  |  |  | *0.15* |
| Yes | 9 | 0.5 | 1.7 | 0.9, 3.4 | 0.12 |  |
| No | 109 | 10.2 | Ref. |  |  |  |
|  |  |  |  |  |  |  |
| *Frequency of hay feeding* |  |  |  |  |  | *0.10* |
| None | 25 | 3.4 | Ref. |  |  |  |
| Once/day | 25 | 2.2 | 1.5 | 0.8, 2.6 | 0.17 |  |
| Twice/day | 28 | 2.3 | 1.6 | 0.9, 2.8 | 0.08 |  |
| >twice/day | 12 | 1.0 | 1.6 | 0.8, 3.1 | 0.21 |  |
| Ad lib | 24 | 1.5 | 2.2 | 1.2, 3.8 | 0.007 |  |
|  |  |  |  |  |  |  |
| Hay soaking |  |  |  |  |  |  |
| *Hay soaked* |  |  |  |  |  | *0.01* |
| Yes | 46 | 3.0 | 1.6 | 1.1, 2.3 | 0.01 |  |
| No/not fed | 72 | 7.6 | Ref. |  |  |  |
|  |  |  |  |  |  |  |
| *Length of time hay soaked* |  |  |  |  |  | *0.12* |
| Not soaked/fed | 72 | 7.6 | Ref. |  |  |  |
| <1hr | 10 | 0.9 | 1.1 | 0.6, 2.2 | 0.74 |  |
| >1-6hrs | 16 | 0.9 | 1.9 | 1.1, 3.2 | 0.02 |  |
| >6 hrs | 17 | 1.2 | 1.5 | 0.9, 2.6 | 0.12 |  |
|  |  |  |  |  |  |  |
| Method of offering forage |  |  |  |  |  |  |
| *Free from ground* |  |  |  |  |  | *0.01* |
| Yes | 26 | 3.5 | 0.6 | 0.4, 0.9 | 0.01 |  |
| No/not fed | 92 | 7.2 | Ref. |  |  |  |
|  |  |  |  |  |  |  |
| *Small-holed haynet* |  |  |  |  |  | *<0.001* |
| Yes | 64 | 3.9 | 2.0 | 1.4, 2.9 | <0.001 |  |
| No/not fed | 54 | 6.7 | Ref. |  |  |  |
|  |  |  |  |  |  |  |
| *Manger, haybar or crate* |  |  |  |  |  | *0.20* |
| Yes | 12 | 1.5 | 0.7 | 0.4, 1.3 | 0.22 |  |
| No/not fed | 106 | 9.2 | Ref. |  |  |  |
|  |  |  |  |  |  |  |
| Hard feed/concentrates |  |  |  |  |  |  |
| *Change in quantity of hard feed in previous month* | |  |  |  |  | *0.15* |
| No change | 89 | 7.4 | Ref. |  |  |  |
| Increase | 6 | 1.0 | 0.5 | 0.2, 1.2 | 0.12 |  |
| Decrease | 22 | 1.6 | 1.2 | 0.7, 1.9 | 0.54 |  |
|  |  |  |  |  |  |  |
| Additional supplements |  |  |  |  |  |  |
| *Supplements fed* |  |  |  |  |  | *0.20* |
| Yes | 93 | 7.5 | 1.3 | 0.9, 2.0 | 0.21 |  |
| No | 30 | 3.2 | Ref. |  |  |  |
|  |  |  |  |  |  |  |
| Types of supplements |  |  |  |  |  |  |
| *Hormone* |  |  |  |  |  | *0.01* |
| Yes | 10 | 0.4 | 2.6 | 1.3, 4.9 | 0.004 |  |
| No | 108 | 10.3 | Ref. |  |  |  |
|  |  |  |  |  |  |  |
| *Anti-laminitic* |  |  |  |  |  | *<0.001* |
| Yes | 20 | 0.7 | 3.0 | 1.9, 4.9 | <0.001 |  |
| No | 98 | 10.0 | Ref. |  |  |  |
|  |  |  |  |  |  |  |
| *Herbal supplement* |  |  |  |  |  | *0.12* |
| Yes | 39 | 2.9 | 1.4 | 0.9, 2.0 | 0.11 |  |
| No | 79 | 7.8 | Ref. |  |  |  |
|  |  |  |  |  |  |  |
| **Ridden exercise (inc. driving)** |  |  |  |  |  |  |
| *Currently ridden* |  |  |  |  |  | *0.04* |
| Yes | 64 | 6.6 | 0.7 | 0.5, 0.98 | 0.04 |  |
| No | 59 | 4.1 | Ref. |  |  |  |
|  |  |  |  |  |  |  |
| Ridden horse’s usual use |  |  |  |  |  |  |
| *Hacking* |  |  |  |  |  | *0.009* |
| Yes | 54 | 6.0 | 0.6 | 0.4, 0.9 | 0.01 |  |
| No | 69 | 4.7 | Ref. |  |  |  |
|  |  |  |  |  |  |  |
| *Schooling* |  |  |  |  |  | *0.009* |
| Yes | 28 | 3.6 | 0.6 | 0.4, 0.9 | 0.01 |  |
| No | 95 | 7.1 | Ref. |  |  |  |
|  |  |  |  |  |  |  |
| *Unaffiliated competition* |  |  |  |  |  | *0.02* |
| Yes | 11 | 1.7 | 0.5 | 0.3, 0.97 | 0.04 |  |
| No | 112 | 9.0 | Ref. |  |  |  |
|  |  |  |  |  |  |  |
| *Professional showing* |  |  |  |  |  | *0.21* |
| Yes | 2 | 0.1 | 2.9 | 0.7, 11.6 | 0.14 |  |
| No | 121 | 10.6 | Ref. |  |  |  |
|  |  |  |  |  |  |  |
| *Stud/breeding* |  |  |  |  |  | *0.12* |
| Yes | 2 | 0.05 | 3.8 | 0.9, 15.4 | 0.06 |  |
| No | 121 | 10.6 | Ref. |  |  |  |
|  |  |  |  |  |  |  |
| *Fitness* |  |  |  |  |  | *0.07* |
| Unfit/not ridden | 98 | 7.7 | 1.5 | 1.0, 2.3 | 0.08 |  |
| Fit | 24 | 2.9 | Ref. |  |  |  |
|  |  |  |  |  |  |  |
| *Current level of work* |  |  |  |  |  | *0.004* |
| More than/same as usual | 29 | 3.8 | Ref. |  |  |  |
| Less than usual/not ridden | 93 | 6.7 | 1.8 | 1.2, 2.7 | 0.007 |  |
|  |  |  |  |  |  |  |
| *Main schooling surface* |  |  |  |  |  | *0.007* |
| Grass | 27 | 1.8 | 2.5 | 1.4, 4.5 | 0.003 |  |
| Rubber | 7 | 0.4 | 3.2 | 1.3, 7.6 | 0.01 |  |
| Sand based | 18 | 2.9 | Ref. |  |  |  |
| Other/not schooled | 71 | 5.5 | 2.1 | 1.3, 3.5 | 0.005 |  |
|  |  |  |  |  |  |  |
| *Main hacking surface* |  |  |  |  |  | *0.001* |
| Off-road tracks and grass | 26 | 1.7 | 2.8 | 1.5, 5.2 | 0.001 |  |
| Roads | 17 | 1.5 | 2.1 | 1.03, 4.1 | 0.04 |  |
| Equal combination of tracks and roads | 16 | 2.9 | Ref. |  |  |  |
| Not hacked | 64 | 4.5 | 2.6 | 1.5, 4.4 | 0.001 |  |
|  |  |  |  |  |  |  |
| Non-ridden horses |  |  |  |  |  |  |
| *Not ridden due to lack of rider/time* |  |  |  |  |  | *0.16* |
| Yes | 17 | 1.0 | 1.5 | 0.9, 2.5 | 0.14 |  |
| No | 106 | 9.6 | Ref. |  |  |  |
|  |  |  |  |  |  |  |
| *Age-related retirement* |  |  |  |  |  | *0.01* |
| Yes | 6 | 1.2 | 0.4 | 0.2, 0.9 | 0.03 |  |
| No | 117 | 9.5 | Ref. |  |  |  |
|  |  |  |  |  |  |  |
| *Recurrent injury/illness-related retirement* | |  |  |  |  | *0.001* |
| Yes | 26 | 1.1 | 2.2 | 1.4, 3.5 | <0.001 |  |
| No | 97 | 9.5 | Ref. |  |  |  |
|  |  |  |  |  |  |  |
| **Transport in the previous year** |  |  |  |  |  |  |
| Main reason for transport in the previous year | |  |  |  |  | *0.06* |
| *Lesson/clinic* |  |  |  |  |  |  |
| Yes | 14 | 1.9 | 0.6 | 0.3, 1.1 | 0.08 |  |
| No | 104 | 8.7 | Ref. |  |  |  |
|  |  |  |  |  |  |  |
| *Long distance/pleasure rides* |  |  |  |  |  | *0.10* |
| Yes | 6 | 1.0 | 0.5 | 0.2, 1.2 | 0.13 |  |
| No | 112 | 9.7 | Ref. |  |  |  |
|  |  |  |  |  |  |  |
| *Moving yards* |  |  |  |  |  | *0.06* |
| Yes | 19 | 1.1 | 1.7 | 1.0, 2.7 | 0.04 |  |
| No | 99 | 9.6 | Ref. |  |  |  |
|  |  |  |  |  |  |  |
| *Time since last transport* |  |  |  |  |  | *0.14* |
| Previous week | 9 | 0.7 | 1.0 | 0.5, 1.9 | 0.93 |  |
| Previous month | 5 | 1.0 | 0.4 | 0.2, 1.0 | 0.05 |  |
| Previous 2 months | 9 | 0.6 | 1.3 | 0.6, 2.5 | 0.51 |  |
| Previous 6 months | 12 | 1.4 | 0.7 | 0.4, 1.3 | 0.24 |  |
| More than 6 months ago/never | 86 | 6.9 | Ref. |  |  |  |
|  |  |  |  |  |  |  |
| *Time since last transport* |  |  |  |  |  | *0.17* |
| Within 6 months | 35 | 3.7 | Ref. |  |  |  |
| More than 6 months ago | 86 | 6.9 | 1.3 | 0.9, 2.0 | 0.17 |  |
|  |  |  |  |  |  |  |
| *Transport time (quartiles)* |  |  |  |  |  | *0.22* |
| 0-25 min | 83 | 7.1 | 1.4 | 0.8, 2.7 | 0.25 |  |
| >25-35 min | 17 | 1.1 | Ref. |  |  |  |
| >35-60 min | 11 | 1.4 | 2.0 | 0.9, 4.2 | 0.08 |  |
| >60 min | 7 | 0.9 | 1.0 | 0.4, 2.5 | 0.93 |  |
|  |  |  |  |  |  |  |
| *Transport distance (quartiles)* |  |  |  |  |  | *0.14* |
| 0-7.5 miles | 83 | 6.9 | Ref. |  |  |  |
| >7.5-15 miles | 15 | 1.3 | 1.0 | 0.6, 1.8 | 0.96 |  |
| >15-30 miles | 14 | 1.1 | 1.1 | 0.4, 1.9 | 0.85 |  |
| > 30 miles | 5 | 1.1 | 0.4 | 0.2, .098 | 0.05 |  |
|  |  |  |  |  |  |  |
| *Transport distance (recategorised)* |  |  |  |  |  | *0.02* |
| 0-30 miles | 112 | 9.3 | Ref. |  |  |  |
| >30 miles | 5 | 1.1 | 0.4 | 0.2, 0.97 | 0.04 |  |
|  |  |  |  |  |  |  |
| **Routine hoof care** |  |  |  |  |  |  |
| *Hoof quality* |  |  |  |  |  | *0.04* |
| Good | 71 | 7.1 | Ref. |  |  |  |
| Average to poor | 52 | 3.5 | 1.5 | 1.03, 2.1 | 0.03 |  |
|  |  |  |  |  |  |  |
| Hoof shape and angle |  |  |  |  |  |  |
| *Fore feet* |  |  |  |  |  | *0.17* |
| Sloping | 10 | 0.5 | 1.9 | 1.0, 3.7 | 0.05 |  |
| Normal | 93 | 8.6 | Ref. |  |  |  |
| Upright | 17 | 1.2 | 1.3 | 0.8, 2.2 | 0.27 |  |
| Other | 2 | 0.3 | 0.6 | 0.1, 2.3 | 0.42 |  |
|  |  |  |  |  |  |  |
| Hoof conditions |  |  |  |  |  |  |
| *White line disease/seedy toe* |  |  |  |  |  | *0.08* |
| Yes | 14 | 0.8 | 1.7 | 1.0, 3.0 | 0.06 |  |
| No | 0.2 | 9.8 | Ref. |  |  |  |
|  |  |  |  |  |  |  |
| *Thrush* |  |  |  |  |  | *0.12* |
| Yes | 4 | 0.7 | 0.5 | 0.2, 1.3 | 0.17 |  |
| No | 113 | 9.9 | Ref. |  |  |  |
|  |  |  |  |  |  |  |
| Hoof care products used |  |  |  |  |  |  |
| *Antibacterial/antifungal/herbal* |  |  |  |  |  | *0.12* |
| Yes | 4 | 0.7 | 0.5 | 0.2, 1.3 | 0.16 |  |
| No | 114 | 9.9 | Ref. |  |  |  |
|  |  |  |  |  |  |  |
| Shoeing |  |  |  |  |  |  |
| *Feet shod* |  |  |  |  |  | *0.20* |
| Full set | 26 | 3.1 | 0.7 | 0.5, 1.1 | 0.15 |  |
| Half set | 24 | 1.8 | 1.1 | 0.7, 1.8 | 0.57 |  |
| Not shod | 69 | 5.8 | Ref. |  |  |  |
|  |  |  |  |  |  |  |
| *Lameness/ soreness after shoeing* |  |  |  |  |  | *0.12* |
| Yes | 5 | 0.2 | 2.2 | 0.9, 5.5 | 0.08 |  |
| No | 114 | 10.4 | Ref. |  |  |  |
|  |  |  |  |  |  |  |
| *Type of shoes on front feet* |  |  |  |  |  | *<0.001* |
| Regular shoes/not shod | 102 | 10.0 | Ref. |  |  |  |
| Heart bar shoes | 13 | 0.3 | 4.0 | 2.3, 7.2 | <0.001 |  |
| Other remedial shoes | 3 | 0.2 | 1.9 | 0.5, 4.6 | 0.52 |  |
|  |  |  |  |  |  |  |
| *Shoes worn on hind feet* |  |  |  |  |  | *0.09* |
| Yes | 27 | 3.1 | 0.7 | 0.5, 1.1 | 0.10 |  |
| No | 96 | 7.5 | Ref. |  |  |  |
|  |  |  |  |  |  |  |
| *Shoe retention* |  |  |  |  |  | *0.07* |
| Very Well | 38 | 3.0 | 1.1 | 0.7, 1.6 | 0.67 |  |
| Well | 10 | 1.7 | 0.5 | 0.2, 0.9 | 0.03 |  |
| Poorly/very poorly | 2 | 0.1 | 1.5 | 0.4, 6.1 | 0.58 |  |
| Not shod | 69 | 5.8 |  |  |  |  |
|  |  |  |  |  |  |  |
| *Lameness/ soreness after trimming* |  |  |  |  |  | *<0.001* |
| Yes | 13 | 0.4 | 3.4 | 1.9, 6.1 | <0.001 |  |
| No | 109 | 10.2 | Ref. |  |  |  |
|  |  |  |  |  |  |  |
| Combined shoeing and trimming variables | |  |  |  |  |  |
| *Shoeing/trimming frequency* |  |  |  |  |  | *0.06* |
| Up to 6 weeks | 64 | 5.0 | 1.5 | 1.0, 2.3 | 0.06 |  |
| 6-8 weeks | 34 | 4.0 | Ref. |  |  |  |
| More than 8 weeks | 23 | 1.5 | 1.8 | 1.1, 3.0 | 0.03 |  |
|  |  |  |  |  |  |  |
| *Shoeing/trimming frequency (re-categorised)* | |  |  |  |  | *0.16* |
| Up to 8 weeks | 98 | 9.0 | Ref. |  |  |  |
| More than 8 weeks | 23 | 1.5 | 1.4 | 0.9, 2.2 | 0.14 |  |
|  |  |  |  |  |  |  |
| *Lameness/soreness after routine foot care* | |  |  |  |  | *<0.001* |
| Yes | 18 | 0.6 | 3.1 | 1.9, 5.1 | <0.001 |  |
| No | 105 | 10.1 | Ref. |  |  |  |
|  |  |  |  |  |  |  |
| **Routine healthcare and current health** | |  |  |  |  |  |
| Routine health care |  |  |  |  |  |  |
| *Dental care frequency* |  |  |  |  |  | *0.17* |
| Every 6 months | 25 | 2.3 | Ref. |  |  |  |
| Once/year | 90 | 7.1 | 1.2 | 0.8, 1.8 | 0.47 |  |
| Less than once/year | 4 | 0.3 | 1.4 | 0.5, 4.0 | 0.54 |  |
| Other/never | 4 | 0.8 | 0.5 | 0.2, 1.3 | 0.15 |  |
|  |  |  |  |  |  |  |
| *Worming care routine* |  |  |  |  |  | *0.08* |
| Set yard schedule | 17 | 1.4 | 1.3 | 0.7, 2.2 | 0.42 |  |
| According to faecal egg count (FEC) | 56 | 5.8 | Ref. |  |  |  |
| Own seasonal worming schedule | 48 | 3.1 | 1.6 | 1.1, 2.4 | 0.01 |  |
| When horse considered to have worms/other/never | 2 | 0.3 | 0.7 | 0.2, 2.9 | 0.64 |  |
|  |  |  |  |  |  |  |
| *Time since last worming* |  |  |  |  |  | *0.06* |
| In the previous month | 27 | 1.7 | Ref. |  |  |  |
| Between 1-6 months | 63 | 5.4 | 0.7 | 0.5, 1.1 | 0.16 |  |
| Between 6 months to 1 year | 18 | 2.5 | 0.5 | 0.2, 0.8 | 0.01 |  |
| More than a year ago | 7 | 0.7 | 0.6 | 0.2, 1.3 | 0.19 |  |
|  |  |  |  |  |  |  |
| *Wormer brand last used* |  |  |  |  |  | *0.005* |
| Equest | 16 | 1.8 | Ref. |  |  |  |
| Equest pramox | 54 | 4.0 | 1.5 | 0.9, 2.6 | 0.15 |  |
| Equimax | 10 | 1.1 | 1.1 | 0.5, 2.3 | 0.89 |  |
| Equitape | 3 | 1.0 | 0.3 | 0.1, 1.2 | 0.09 |  |
| Eqvalan | 1 | 0.4 | 0.3 | 0.04, 2.2 | 0.24 |  |
| Eqvalan duo | 7 | 0.4 | 1.9 | 0.8, 4.8 | 0.14 |  |
| Eraquell | 4 | 0.2 | 2.5 | 0.8, 7.4 | 0.11 |  |
| Panacur | 14 | 0.6 | 2.5 | 1.2, 5.0 | 0.01 |  |
| Strongid-P | 5 | 0.3 | 1.8 | 0.7, 5.0 | 0.24 |  |
| Other/none | 7 | 0.9 | 0.9 | 0.4, 2.2 | 0.83 |  |
|  |  |  |  |  |  |  |
| *Active wormer ingredients in last wormer given* | |  |  |  |  | *0.002* |
| Benzi-midazoles | 15 | 0.6 | 2.9 | 1.5, 5.6 | 0.001 |  |
| Macrocyclic lactones | 23 | 2.6 | Ref. |  |  |  |
| Macrocyclic lactones and praziquantels | 72 | 5.5 | 1.5 | 0.9, 2.4 | 0.11 |  |
| Praziquantels | 3 | 1.0 | 0.3 | 0.1, 1.2 | 0.09 |  |
| Tetrahydro-pirimidines | 5 | 0.5 | 1.1 | 0.4, 2.9 | 0.85 |  |
| Other/none | 3 | 0.4 | 0.9 | 0.3, 3.1 | 0.91 |  |
|  |  |  |  |  |  |  |
| *Active wormer ingredients in last wormer given (re-categorised)* | | |  |  |  | *0.009* |
| Benzimidazoles | 15 | 0.6 | 2.9 | 1.5, 5.6 | 0.001 |  |
| Macrocyclic lactones | 23 | 2.6 | Ref. |  |  |  |
| Other/none | 83 | 7.3 | 1.3 | 0.8, 2.0 | 0.31 |  |
|  |  |  |  |  |  |  |
| *Faecal egg count (FEC) use* |  |  |  |  |  | *0.15* |
| Yes | 74 | 7.0 | 0.8 | 0.5, 1.1 | 0.15 |  |
| No | 49 | 3.6 | Ref. |  |  |  |
|  |  |  |  |  |  |  |
| *FEC frequency* |  |  |  |  |  | *0.06* |
| Once per year or less | 64 | 5.0 | Ref. |  |  |  |
| Twice/year | 24 | 2.3 | 0.8 | 0.5, 1.3 | 0.34 |  |
| Three times per year | 9 | 1.6 | 0.4 | 0.2, 0.9 | 0.02 |  |
| Four times per year | 18 | 1.4 | 1.0 | 0.6, 1.7 | 0.96 |  |
| Five times per year or more | 5 | 0.2 | 1.7 | 0.7, 4.1 | 0.27 |  |
|  |  |  |  |  |  |  |
| Foaling |  |  |  |  |  |  |
| *Mare in foal previously* |  |  |  |  |  | *0.09* |
| Yes | 22 | 1.2 | 1.4 | 0.8, 2.4 | 0.20 |  |
| No | 33 | 2.7 | Ref. |  |  |  |
| Don’t know | 4 | 0.7 | 0.5 | 0.2, 1.4 | 0.19 |  |
|  |  |  |  |  |  |  |
| *Mare currently in foal* |  |  |  |  |  | *0.10* |
| Yes | 1 | 0.01 | 9.4 | 1.2, 71.9 | 0.03 |  |
| No | 58 | 4.5 | Ref. |  |  |  |
|  |  |  |  |  |  |  |
| Non-laminitic lameness |  |  |  |  |  |  |
| *Current lameness* |  |  |  |  |  | *0.23* |
| Yes | 22 | 1.5 | 1.3 | 0.8, 2.1 | 0.21 |  |
| No | 101 | 9.1 | Ref. |  |  |  |
|  |  |  |  |  |  |  |
| Type of lameness |  |  |  |  |  |  |
| *Soft tissue injury (tendon/ligament)* |  |  |  |  |  | *0.006* |
| Yes | 7 | 0.2 | 3.6 | 1.7, 7.8 | 0.001 |  |
| No | 112 | 10.4 | Ref. |  |  |  |
|  |  |  |  |  |  |  |
| *Bone fracture* |  |  |  |  |  | *0.13* |
| Yes | 1 | 0.01 | 7.4 | 1.0, 53.9 | 0.05 |  |
| No | 118 | 10.6 | Ref. |  |  |  |
|  |  |  |  |  |  |  |
| *Undetermined lameness* |  |  |  |  |  | *0.05* |
| Yes | 6 | 0.2 | 2.6 | 1.2, 6.0 | 0.02 |  |
| No | 113 | 10.4 | Ref. |  |  |  |
|  |  |  |  |  |  |  |
| Leg associated with lameness |  |  |  |  |  |  |
| *Right foreleg* |  |  |  |  |  | *0.14* |
| Yes | 11 | 0.6 | 1.6 | 0.9, 3.1 | 0.12 |  |
| No | 108 | 10.0 | Ref. |  |  |  |
|  |  |  |  |  |  |  |
| *Left foreleg* |  |  |  |  |  | *0.17* |
| Yes | 10 | 0.6 | 1.6 | 0.8, 3.1 | 0.15 |  |
| No | 109 | 10.0 | Ref. |  |  |  |
|  |  |  |  |  |  |  |
| *Left hindleg* |  |  |  |  |  | *0.16* |
| Yes | 10 | 0.6 | 1.7 | 0.9, 3.2 | 0.13 |  |
| No | 109 | 10.0 | Ref. |  |  |  |
|  |  |  |  |  |  |  |
| Disease management |  |  |  |  |  |  |
| *Current box rest* |  |  |  |  |  | *0.04* |
| Yes | 6 | 0.2 | 2.7 | 1.2, 6.2 | 0.02 |  |
| No | 117 | 10.5 | Ref. |  |  |  |
|  |  |  |  |  |  |  |
| *Days spent on box rest* |  |  |  |  |  | *0.05* |
| 0 days | 117 | 10.5 | Ref. |  |  |  |
| 1 to 7 days | 3 | 0.1 | 5.3 | 1.7, 16.8 | 0.004 |  |
| More than 7 days | 3 | 0.2 | 1.8 | 0.6, 5.8 | 0.30 |  |
|  |  |  |  |  |  |  |
| *Behaviour change while on box rest* |  |  |  |  |  | *0.05* |
| Yes | 3 | 0.1 | 4.1 | 1.3, 13.0 | 0.02 |  |
| No/not on box rest | 120 | 10.6 | Ref. |  |  |  |
|  |  |  |  |  |  |  |
| *Ability to see/interact with other horses while on box rest* | | |  |  |  | *0.01* |
| Not on box rest | 117 | 10.5 | Ref. |  |  |  |
| Most of the time while on box rest | 1 | 0.1 | 0.8 | 0.1, 5.6 | 0.80 |  |
| Some/none of the time while on box rest | 5 | 0.1 | 5.5 | 2.2, 13.4 | <0.001 |  |
|  |  |  |  |  |  |  |
| Current health |  |  |  |  |  |  |
| *Any existing health conditions* |  |  |  |  |  | *0.05* |
| Yes | 70 | 5.1 | 1.4 | 1.0, 2.0 | 0.05 |  |
| No | 53 | 5.6 | Ref. |  |  |  |
|  |  |  |  |  |  |  |
| Type of health condition |  |  |  |  |  |  |
| *Abnormal oestrous cycles* |  |  |  |  |  | *0.05* |
| Yes | 2 | 0.03 | 6.3 | 1.5, 25.6 | 0.01 |  |
| No | 121 | 10.6 | Ref. |  |  |  |
|  |  |  |  |  |  |  |
| *Pituitary pars intermedia dysfunction (PPID)* | |  |  |  |  | *0.04* |
| Yes | 33 | 2.0 | 1.5 | 1.0, 2.3 | 0.03 |  |
| No | 90 | 8.7 | Ref. |  |  |  |
|  |  |  |  |  |  |  |
| *Equine metabolic syndrome (EMS)/Insulin resistance(IR)* | | |  |  |  | *<0.001* |
| Yes | 39 | 1.5 | 2.9 | 1.9, 4.2 | <0.001 |  |
| No | 84 | 9.2 | Ref. |  |  |  |
|  |  |  |  |  |  |  |
| *Combined PPID and/or EMS/IR* |  |  |  |  |  | *<0.001* |
| Yes | 56 | 2.9 | 2.2 | 1.5, 3.1 | <0.001 |  |
| No | 67 | 7.8 | Ref. |  |  |  |
|  |  |  |  |  |  |  |
| Prescription medication |  |  |  |  |  |  |
| *Currently receiving medication* |  |  |  |  |  | *<0.001* |
| Yes | 52 | 2.6 | 2.2 | 1.6, 3.2 | <0.001 |  |
| No | 71 | 8.0 | Ref. |  |  |  |
|  |  |  |  |  |  |  |
| Type of medication currently receiving | |  |  |  |  |  |
| *Oral anti-inflammatory* |  |  |  |  |  | *<0.001* |
| Yes | 30 | 1.1 | 2.9 | 1.9, 4.4 | <0.001 |  |
| No | 88 | 9.5 | Ref. |  |  |  |
|  |  |  |  |  |  |  |
| *Injected anti-inflammatory* |  |  |  |  |  |  |
| Yes | 3 | 0.1 | 4.0 | 1.3, 12.7 | 0.02 | *0.05* |
| No | 115 | 10.6 | Ref. |  |  |  |
|  |  |  |  |  |  |  |
| *Combined injected and/or oral anti-inflammatories* | |  |  |  |  | *<0.001* |
| Yes | 31 | 1.2 | 2.8 | 1.9, 4.2 | <0.001 |  |
| No | 92 | 9.5 | Ref. |  |  |  |
|  |  |  |  |  |  |  |
| *Oral hormone regulator* |  |  |  |  |  | *0.005* |
| Yes | 31 | 1.7 | 1.9 | 1.2, 2.8 | 0.003 |  |
| No | 87 | 9.0 | Ref. |  |  |  |
|  |  |  |  |  |  |  |
| *Injected antibiotic* |  |  |  |  |  | *0.13* |
| Yes | 1 | 0.01 | 7.8 | 1.1, 56.1 | 0.04 |  |
| No | 117 | 10.7 | Ref. |  |  |  |
|  |  |  |  |  |  |  |
| *Non-steroidal anti-inflammatories (NSAIDs)* | |  |  |  |  | *<0.001* |
| Yes | 28 | 1.1 | 2.8 | 1.8, 4.2 | <0.001 |  |
| No | 90 | 9.6 | Ref. |  |  |  |
|  |  |  |  |  |  |  |
| *Steroidal anti-inflammatories* |  |  |  |  |  | *0.008* |
| Yes | 5 | 0.1 | 4.5 | 1.8, 11.1 | 0.001 |  |
| No | 113 | 10.6 | Ref. |  |  |  |
|  |  |  |  |  |  |  |
| *Pergolide mesylate* |  |  |  |  |  | *0.06* |
| Yes | 25 | 1.5 | 1.6 | 1.0, 2.5 | 0.05 |  |
| No | 93 | 9.1 | Ref. |  |  |  |
|  |  |  |  |  |  |  |
| **Laminitis history** |  |  |  |  |  |  |
| *Previous history of laminitis* |  |  |  |  |  | *<0.001* |
| Yes | 100 | 4.4 | 6.0 | 3.8, 9.5 | <0.001 |  |
| No | 23 | 6.2 | Ref. |  |  |  |
|  |  |  |  |  |  |  |
| *Previous laminitis before current owner took over care* | | |  |  |  | *0.22* |
| Yes | 14 | 0.9 | 1.4 | 0.8, 2.5 | 0.20 |  |
| No | 109 | 9.8 | Ref. |  |  |  |
|  |  |  |  |  |  |  |
| *Previous laminitis while with current owner* | |  |  |  |  | *<0.001* |
| Yes | 99 | 4.1 | 6.5 | 4.1, 10.1 | <0.001 |  |
| No | 24 | 6.5 | Ref. |  |  |  |
|  |  |  |  |  |  |  |
| *Previous laminitis while with current owner (combined with veterinary-diagnosis)* | | | |  |  | *<0.001* |
| Yes, veterinary-diagnosed | 53 | 2.9 | 4.9 | 3.0, 7.9 | <0.001 |  |
| Yes, not veterinary-diagnosed | 46 | 1.2 | 10.4 | 6.4, 17.1 | <0.001 |  |
| No | 24 | 6.5 | Ref. |  |  |  |
|  |  |  |  |  |  |  |
| *Return to soundness following most recent episode* | |  |  |  |  | *<0.001* |
| Yes/no previous laminitis | 109 | 10.2 | Ref. |  |  |  |
| No | 14 | 0.4 | 3.4 | 2.0, 6.0 | <0.001 |  |
|  |  |  |  |  |  |  |
| *Time taken to return to soundness following most recent episode* | | |  |  |  | *<0.001* |
| Less than 2 weeks | 41 | 7.7 | Ref. |  |  |  |
| Between 2 weeks to 2 months | 36 | 1.4 | 4.8 | 3.0, 7.5 | <0.001 |  |
| More than 2 months | 30 | 1.0 | 5.5 | 3.4, 8.8 | <0.001 |  |
|  |  |  |  |  |  |  |
| *Return to same level of prior work following most recent episode* | | |  |  |  |  |
| Yes/ no previous laminitis | 68 | 8.7 | Ref. |  |  | *<0.001* |
| No/horse not in prior work | 54 | 1.9 | 3.7 | 2.6, 5.3 | <0.001 |  |
|  |  |  |  |  |  |  |

HYAR – Horse-years at risk; LRS – Likelihood ratio statistic; Ref. – Referent group in which the Hazard Ratio = 1.0; 95% CI – 95% Confidence Interval
