# Supplementary material for: Identification of modifiable factors associated with owner-reported equine laminitis in Britain using a web-based cohort study approach
Source: BMC Vet Res. 2019 Feb 12;15:59. doi: 10.1186/s12917-019-1798-8 (PMC6373032; doi:10.1186/s12917-019-1798-8)
Supplement: Supplementary file 6 — A table showing the multivariable Cox regression interim models of variables associated with laminitis (n = 123 episodes) in a cohort study of horses and ponies in Great Britain. (DOCX 37 kb) [file 12917_2019_1798_MOESM6_ESM.docx]

**Table 3:** Multivariable Cox regression interim models of variables associated with laminitis (n=123 episodes) in a cohort study of horses and ponies in Great Britain.

| **Variable** | **No. laminitis episodes** | **HYAR/100** | **Hazard ratio** | **95% CI** | **Wald**  **P-value** | **LRS^#^**  **P-value** |
| --- | --- | --- | --- | --- | --- | --- |
| **Animal level factors model** | |  |  |  |  |  |
| *Breed category (including breed crosses)* | |  |  |  |  | *<0.001* |
| Native pony | 79 | 4.3 | 2.6 | 1.8, 3.8 | <0.001 |  |
| Other breed | 44 | 6.3 | Ref. |  |  |  |
|  |  |  |  |  |  |  |
| *Number of horses enrolled* |  |  |  |  |  | *0.005* |
| One horse | 62 | 4.7 | Ref. |  |  |  |
| Two horses | 41 | 2.8 | 1.1 | 0.7, 1.6 | 0.71 |  |
| More than two horses | 20 | 3.1 | 0.5 | 0.3, 0.8 | 0.007 |  |
|  |  |  |  |  |  |  |
| *Age category* |  |  |  |  |  | *0.03* |
| Less than 12 years | 25 | 3.1 | Ref. |  |  |  |
| 12 to 16 years | 29 | 2.5 | 1.5 | 0.9, 2.5 | 0.15 |  |
| 17 to 20 years | 40 | 2.4 | 2.1 | 1.3, 3.5 | 0.003 |  |
| More than 20 years | 29 | 2.7 | 1.4 | 0.8, 2.3 | 0.26 |  |
|  |  |  |  |  |  |  |
| *Weight change since last submission* | |  |  |  |  | *0.04* |
| Maintained/lost | 85 | 8.4 | Ref. |  |  |  |
| Gained | 35 | 2.1 | 1.7 | 1.1, 2.6 | 0.01 |  |
| Not specified | 3 | 0.1 | 1.4 | 0.4, 4.5 | 0.57 |  |
|  |  |  |  |  |  |  |
| **Turnout and management of grazing model** | |  |  |  |  |  |
| *Wearing of grazing muzzle* |  |  |  |  |  | *0.003* |
| Part of the time while grazing | 12 | 0.3 | 2.9 | 1.6, 5.3 | 0.001 |  |
| All of the time while grazing /not worn | 107 | 10.3 | Ref. |  |  |  |
|  |  |  |  |  |  |  |
| *Reintroduction to grass within past month* | |  |  |  |  | *0.004* |
| Yes | 17 | 0.5 | 2.3 | 1.4, 4.0 | 0.002 |  |
| No/no grass access | 102 | 10.1 | Ref. |  |  |  |
|  |  |  |  |  |  |  |
| *Agricultural crop land bordering turnout area* | |  |  |  |  | *0.01* |
| Yes | 31 | 3.9 | 0.6 | 0.4, 0.9 | 0.02 |  |
| No/not turned out | 88 | 6.7 | Ref. |  |  |  |
|  |  |  |  |  |  |  |
| *Time of day access to grass available* | |  |  |  |  | *0.02* |
| Morning only | 11 | 0.4 | 2.6 | 1.2-5.5 | 0.01 |  |
| Other/grass not available | 75 | 5.3 | 1.7 | 1.1-2.6 | 0.02 |  |
| Day and night | 33 | 4.9 | Ref. |  |  |  |
|  |  |  |  |  |  |  |
| *Woodland bordering turnout area* |  |  |  |  |  | *0.02* |
| Yes | 21 | 2.8 | 0.6 | 0.4, 0.9 | 0.03 |  |
| No/not turned out | 98 | 7.8 | Ref. |  |  |  |
|  |  |  |  |  |  |  |
| *Turnout restricted to restrict grass intake* | |  |  |  |  | *0.02* |
| Yes | 36 | 1.6 | 1.7 | 1.1, 2.6 | 0.01 |  |
| No/grass not available | 83 | 9.0 | Ref. |  |  |  |
|  |  |  |  |  |  |  |
| **Stabling and indoor environment model** | |  |  |  |  |  |
| *Stabling to reduce grass intake* |  |  |  |  |  | *<0.001* |
| Yes | 63 | 3.0 | 2.6 | 1.6, 4.3 | <0.001 |  |
| No/not stabled | 55 | 7.6 | Ref. |  |  |  |
|  |  |  |  |  |  |  |
| *Average time spent stabled* |  |  |  |  |  | *0.006* |
| Not stabled/free access | 40 | 5.2 | 3.2 | 1.4, 7.5 | 0.007 |  |
| Up to 12 hours | 55 | 3.0 | 1.9 | 1.1, 3.0 | 0.01 |  |
| More than 12 hours | 23 | 2.4 | Ref. |  |  |  |
|  |  |  |  |  |  |  |
| *Bedding used in stable* |  |  |  |  |  | *0.04* |
| Yes | 84 | 5.6 | 2.4 | 1.1, 5.5 | 0.03 |  |
| No/not stabled | 34 | 5.0 | Ref. |  |  |  |
|  |  |  |  |  |  |  |
| **Supplementary feeding model** |  |  |  |  |  |  |
| *Anti-laminitic supplements* |  |  |  |  |  | *0.002* |
| Yes | 20 | 0.7 | 2.4 | 1.4, 3.9 | 0.001 |  |
| No | 98 | 9.9 | Ref. |  |  |  |
|  |  |  |  |  |  |  |
| *Forage offered in a small-holed net* | |  |  |  |  | *0.01* |
| Yes | 64 | 3.9 | 1.6 | 1.1, 2.4 | 0.01 |  |
| No/not fed | 54 | 6.7 | Ref. |  |  |  |
|  |  |  |  |  |  |  |
| *Hormone supplements* |  |  |  |  |  | *0.03* |
| Yes | 10 | 0.3 | 2.3 | 1.2, 4.5 | 0.02 |  |
| No | 108 | 10.2 | Ref. |  |  |  |
|  |  |  |  |  |  |  |
| *Forage offered free from the ground* | |  |  |  |  | *0.03* |
| Yes | 26 | 3.4 | 0.6 | 0.4, 0.97 | 0.04 |  |
| No/not fed | 92 | 7.1 | Ref. |  |  |  |
|  |  |  |  |  |  |  |
| **Exercise model** |  |  |  |  |  |  |
| *Age-related retirement* |  |  |  |  |  | *<0.001* |
| Yes | 6 | 1.2 | 0.3 | 0.1, 0.6 | 0.002 |  |
| No | 112 | 9.4 | Ref. |  |  |  |
|  |  |  |  |  |  |  |
| *Recurrent injury/illness-related retirement* | |  |  |  |  | *0.006* |
| Yes | 26 | 1.1 | 2.1 | 1.3, 3.5 | 0.004 |  |
| No | 92 | 9.5 | Ref. |  |  |  |
|  |  |  |  |  |  |  |
| *Main schooling surface* |  |  |  |  |  | *0.01* |
| Grass | 25 | 1.8 | 2.5 | 1.4, 4.6 | 0.003 |  |
| Rubber | 6 | 0.4 | 2.9 | 1.2, 7.1 | 0.02 |  |
| Sand based | 17 | 2.9 | Ref. |  |  |  |
| Other/not schooled | 70 | 5.5 | 1.6 | 0.8, 3.2 | 0.18 |  |
|  |  |  |  |  |  |  |
| *Main hacking surface* |  |  |  |  |  | *0.02* |
| Off-road | 22 | 1.6 | 2.6 | 1.4, 4.8 | 0.003 |  |
| Roads | 17 | 1.5 | 2.2 | 1.1, 4.3 | 0.03 |  |
| Off-road and roads | 16 | 2.9 | Ref. |  |  |  |
| Not hacked/not ridden | 63 | 4.5 | 2.0 | 1.0, 4.1 | 0.06 |  |
|  |  |  |  |  |  |  |
| *Current level of work* |  |  |  |  |  | *0.04* |
| More than/same as usual | 26 | 3.8 | Ref. |  |  |  |
| Less than usual/not ridden | 91 | 6.7 | 1.6 | 1.01, 2.7 | 0.04 |  |
|  |  |  |  |  |  |  |
| **Transport model** |  |  |  |  |  |  |
| *Transport distance during most recent journey* | |  |  |  |  | *0.008* |
| 0-30 miles | 107 | 9.3 | Ref. |  |  |  |
| >30 miles | 5 | 1.1 | 0.3 | 0.1, 0.9 | 0.02 |  |
|  |  |  |  |  |  |  |
| *Main reason for transport in the previous year* | |  |  |  |  | *0.03* |
| Moving yards | 19 | 1.1 | 1.9 | 1.1, 2.3 | 0.02 |  |
| Other reason/not transported | 99 | 9.5 | Ref. |  |  |  |
|  |  |  |  |  |  |  |
| **Hoof care model** |  |  |  |  |  |  |
| *Type of shoes on front feet* |  |  |  |  |  | *0.001* |
| Regular shoes/not shod | 102 | 10.0 | Ref. |  |  |  |
| Heart bar shoes | 12 | 0.3 | 4.2 | 2.1, 8.3 | <0.001 |  |
| Other remedial shoes | 3 | 0.2 | 1.7 | 0.5, 5.6 | 0.37 |  |
|  |  |  |  |  |  |  |
| *Shoe retention* |  |  |  |  |  | *0.001* |
| Very Well | 38 | 2.9 | 0.9 | 0.6, 1.5 | 0.75 |  |
| Well | 9 | 1.7 | 0.3 | 0.1, 0.6 | 0.001 |  |
| Poorly/very poorly | 2 | 0.1 | 0.9 | 0.2, 3.7 | 0.86 |  |
| Not shod | 69 | 5.8 | Ref. |  |  |  |
|  |  |  |  |  |  |  |
| *Lameness/soreness after routine foot care* | |  |  |  |  | *0.002* |
| Yes | 18 | 10.1 | 2.6 | 1.5, 4.5 | 0.001 |  |
| No | 100 | 0.5 | Ref. |  |  |  |
|  |  |  |  |  |  |  |
| *Shoeing/trimming frequency* |  |  |  |  |  | *0.02* |
| Up to 6 weeks | 62 | 5.0 | 1.6 | 1.03, 2.5 | 0.04 |  |
| 6-8 weeks | 32 | 4.0 | Ref. |  |  |  |
| More than 8 weeks | 23 | 1.5 | 2.0 | 1.2, 3.5 | 0.1 |  |
|  |  |  |  |  |  |  |
| *Hoof quality* |  |  |  |  |  | *0.02* |
| Good | 68 | 7.1 | Ref. |  |  |  |
| Average to poor | 50 | 3.5 | 1.6 | 1.1, 2.4 | 0.02 |  |
|  |  |  |  |  |  |  |
| *Thrush* |  |  |  |  |  | *0.02* |
| Yes | 4 | 0.7 | 0.4 | 0.1, 0.99 | 0.05 |  |
| No | 113 | 9.8 | Ref. |  |  |  |
|  |  |  |  |  |  |  |
| **Health management and recent health history model** | | |  |  |  |  |
| *Current equine metabolic syndrome/insulin resistance* | | |  |  |  | *<0.001* |
| Yes | 38 | 1.5 | 2.8 | 1.9, 4.2 | <0.001 |  |
| No | 80 | 9.1 | Ref. |  |  |  |
|  |  |  |  |  |  |  |
| *Currently on non-steroidal anti-inflammatories* | |  |  |  |  | *<0.001* |
| Yes | 28 | 1.1 | 2.4 | 1.6, 3.8 | <0.001 |  |
| No | 90 | 9.5 | Ref. |  |  |  |
|  |  |  |  |  |  |  |
| *Currently on steroidal anti-inflammatories* | |  |  |  |  | *0.009* |
| Yes | 5 | 0.1 | 4.4 | 1.8, 11.1 | 0.002 |  |
| No | 113 | 10.8 | Ref. |  |  |  |
|  |  |  |  |  |  |  |
| *Soft tissue lameness (e.g. tendon/ligament)* | |  |  |  |  | *0.01* |
| Yes | 7 | 0.2 | 3.3 | 1.5, 7.2 | 0.003 |  |
| No | 111 | 10.4 | Ref. |  |  |  |
|  |  |  |  |  |  |  |
| *Active wormer ingredients in last wormer given* | |  |  |  |  | *0.04* |
| Benzimidazoles | 15 | 0.6 | 2.4 | 1.2, 4.7 | 0.009 |  |
| Macrocyclic lactones | 23 | 2.6 | Ref. |  |  |  |
| Other/none | 78 | 7.3 | 1.2 | 0.7, 1.9 | 0.54 |  |
|  |  |  |  |  |  |  |
| *Time since last worming* |  |  |  |  |  | *0.05* |
| In the previous month | 27 | 1.6 | Ref. |  |  |  |
| Between 1-6 months | 63 | 5.4 | 0.7 | 0.4, 1.1 | 0.10 |  |
| Between 6 months to 1 year | 18 | 2.5 | 0.4 | 0.2, 0.8 | 0.007 |  |
| More than a year ago | 7 | 0.7 | 0.5 | 0.2, 1.2 | 0.15 |  |
|  |  |  |  |  |  |  |
| **Previous laminitis history model** | |  |  |  |  |  |
| *Previous laminitis while with current owner* | |  |  |  |  | *<0.001* |
| Yes, veterinary-diagnosed | 52 | 2.9 | 2.5 | 1.3, 4.9 | 0.009 |  |
| Yes, not veterinary-diagnosed | 42 | 1.2 | 6.6 | 3.5, 12.7 | <0.001 |  |
| No | 24 | 6.5 | Ref. |  |  |  |
|  |  |  |  |  |  |  |
| *Time taken to return to soundness following most recent episode* | | | |  |  | *0.006* |
| Less than 2 weeks | 39 | 7.6 | Ref. |  |  |  |
| Between 2 weeks to 2 months | 34 | 1.4 | 1.9 | 1.1, 3.4 | 0.03 |  |
| More than 2 months | 30 | 1.0 | 2.6 | 1.4, 4.7 | 0.002 |  |
|  |  |  |  |  |  |  |

HYAR – Horse-years at risk; LRS – Likelihood ratio statistic; Ref. – Referent group in which the Hazard Ratio = 1.0; 95% CI – 95% Confidence Interval
